# Less can be more for predicting properties with large language models

**DOI:** 10.1038/s43588-026-01045-2

**Published:** 2026-10-12

**Authors:** Nawaf Alampara, Santiago Miret, Kevin Maik Jablonka

**Affiliations:** 1 Laboratory of Organic and Macromolecular Chemistry, Friedrich Schiller University Jena, Jena, Germany; 2 Intel Labs, Santa Clara, CA USA; 3 Center for Energy and Environmental Chemistry Jena, Friedrich Schiller University Jena, Jena, Germany; 4 Helmholtz Institute for Polymers in Energy Applications Jena, Jena, Germany; 5 Jena Center for Soft Matter, Friedrich Schiller University Jena, Jena, Germany; 6 Present Address: Lila Sciences, Cambridge, MA USA

**Keywords:** Materials science, Computational science

## Abstract

Predicting properties from coordinate–category data—sets of vectors paired with categorical information—is fundamental to computational science, manifesting in materials science as predicting formation energies or elastic moduli from crystal structures. While large language models (LLMs) have increasingly been applied to such tasks, optimal strategies for achieving reliable predictions remain elusive. Here we report fundamental limitations in LLM’s ability to learn from coordinate information in coordinate–category data. Through systematic experiments using synthetic datasets with tunable coordinate and category contributions, and a comprehensive benchmarking framework (MatText), we find that LLMs consistently fail to capture coordinate information while excelling at category patterns. This geometric blindness persists regardless of model size (up to 70 billion parameters), dataset scale (up to 2 × 10^6^ structures) or text representation strategy. For material property prediction tasks dominated by structural effects, specialized geometric architectures consistently outperform LLMs, as evidenced by a clear ‘Graph Neural Network Language Model (GNN-LM) wall’ in performance benchmarks. Our analysis provides concrete guidelines for architecture selection in scientific machine learning.

## Abstract (web summary)

Researchers show that large language models cannot learn from spatial coordinates regardless of model or data scale. Specialized geometric neural networks consistently outperform language models for material property prediction.

## Main

Large language models (LLMs) have matched or exceeded human expert performance across diverse scientific domains^1,2^. This success has prompted researchers to apply LLMs to property prediction tasks in chemistry and materials science, with several studies reporting encouraging results^3–6^. Yet, these achievements are difficult to reconcile with documented failure modes—some as basic as counting letters in a word^7–10^.

**Fig. 1:**
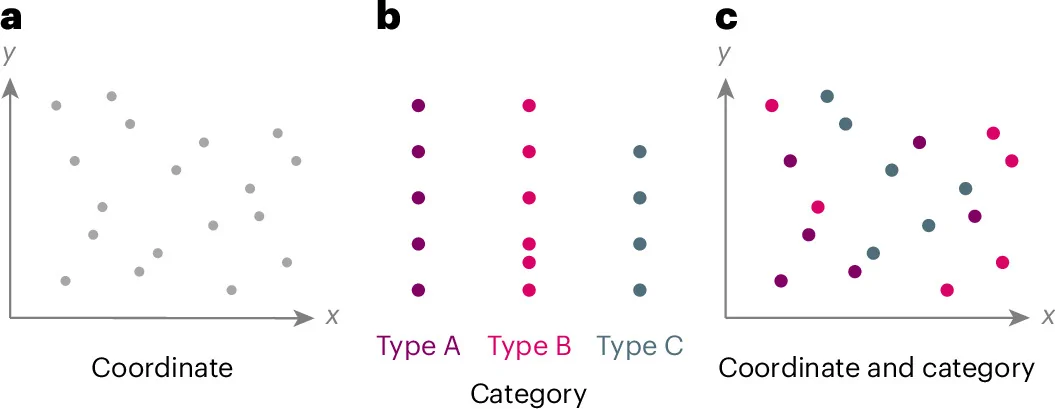
Illustration of a data point at various levels of information. **a**, Coordinate information consists of continuous positional coordinates. **b**, Category information consists of discrete categorical labels. **c**, Coordinate–category data require joint modeling of both positional coordinates and discrete categorical labels.

Machine learning models in scientific applications encounter data that contain both coordinate information (vectors) and category information (discrete categorical labels or types), illustrated in Fig. 1.

Property prediction from coordinate–category data (data combining vectors with discrete categorical information) is an important example of a scientific prediction task. It represents a fundamental challenge across the physical sciences. The task requires predicting properties (such as energies, decay rates and elastic moduli) from data where vector information (for example, atomic positions and momentum vectors) is paired with category information (for example, atom types, molecular labels and particle classifications). Materials science, for example, has long relied on domain-specific models with hand-crafted features or Graph Neural Networks (GNNs) for these predictions^11,12^. Now, researchers increasingly turn to LLMs as a general-purpose alternative. This shift raises a critical question: can LLMs effectively learn the coordinate relationships encoded in such coordinate–category data? Understanding the capabilities and limitations of an LLM’s abilities has profound implications for materials design and discovery, where accurate property prediction accelerates the search for novel materials.

In this Article, we show that transformer-based models, including both causal and masked language models, systematically fail to learn from coordinate information on coordinate–category data. Through controlled experiments using synthetic datasets where we can precisely tune the importance of the coordinate information (for example, atom position vectors) versus category information (for example, atom types), we discover that LLMs are drastically less sample-efficient than conventional approaches when learning from coordinate data.

While LLMs excel at learning from the category information (which elements are present), they struggle with coordinate relationships (how atoms are arranged in space). This modeling gap persists across model architectures, scales and training strategies. Systematically understanding when and why LLMs succeed or fail at scientific prediction tasks is essential for their reliable deployment. Our results provide a framework for understanding when to deploy LLMs versus specialized models for scientific tasks. Rather than viewing LLMs as universal replacements for domain-specific approaches, our work delineates their capabilities and limitations, guiding researchers toward more effective modeling strategies for diverse property-prediction tasks.

## Results

### Probing LLMs with coordinate–category information mixtures

To understand how LLMs perform in predicting data containing both coordinate and category information, we created synthetic datasets. Each data point consists of 3D coordinates (coordinate information, analogous to position of atoms) and an associated categorical label (analogous to atom types). These datasets are inspired by materials or molecular property-prediction tasks, where the objective is to predict the property from coordinates and atom types.

We computed scalar target values for these coordinate–category data points using a physics-inspired hypothetical potential function (which can be thought of as an energy function). To that end, we construct the hypothetical potential such that we can continuously tune the impact of categories (that is, types) versus coordinate information (equation (1)). We give a more detailed explanation of equation (1) in ‘The physics-inspired hypothetical potential’ in the Methods.

1 $$E=\alpha {E}_{\mathrm{category}}+(1-\alpha ){E}_{\mathrm{coordinate}}\,\alpha \in [0,1].$$

In the extreme of *α* = 0, the target values depend solely on *E*_coordinate_ (the energy contribution from the coordinate information, that is, the positions of the points in space), while for *α* = 1, the labels depend only on *E*_category_ (the energy contribution from the category information, that is, the types of points). On the basis of equation (1) and our goal of elucidating effects from coordinate and category data types, we generated synthetic datasets with diverse coordinate and category configurations. Some datasets have points arranged in regular patterns (for instance, cube corners), while others feature different coordinate configurations, and vary in their number of category types. For each dataset, we generate multiple versions by varying *α* (for example *α* = 0, 0.2, ⋯, 1) that span the spectrum from purely coordinate-dependent to purely category-dependent energy landscapes. This controlled *α*-sweep framework allows us to isolate and study how LLMs learn from coordinate versus category information, while ensuring our findings generalize across various coordinate geometries and category distributions.

**Fig. 2:**
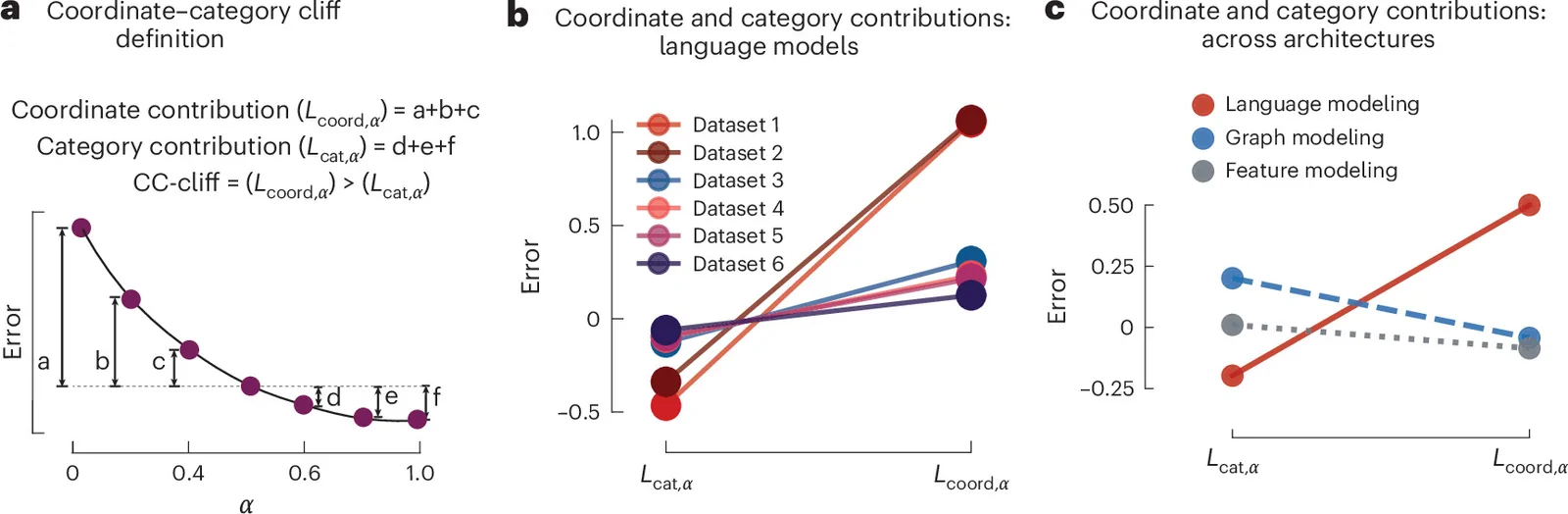
Differential ability of model architectures to learn from categorical versus coordinate data. **a**, A schematic of the coordinate contribution (*L*_coord,*α*_) and category contribution (*L*_cat,*α*_) metrics. The mixing parameter *α* ∈ [0,1] controls the balance between coordinate and category information in the input; *α* = 0 is purely coordinate and *α* = 1 is purely categorical. *L*_coord,*α*_ and *L*_cat,*α*_ are defined as $${L}_{\mathrm{coord},\alpha }={\sum }_{\alpha \in {\alpha }_{g}}\,\mathrm{loss}\,(\alpha )3\times \mathrm{loss}\,(0.5)$$ and $${L}_{\mathrm{cat},\alpha }={\sum }_{\alpha \in {\alpha }_{c}}\,\mathrm{loss}\,(\alpha )$$$$-3\times \mathrm{loss}\,(0.5)$$, where *α*_*g*_ = {0, 0.2, 0.4} and *α*_*c*_ = {0.6, 0.8, 1.0}. **b**, *L*_coord,*α*_ and *L*_cat,*α*_ for a language model across six datasets. A positive CC-cliff (that is, *L*_coord,*α*_ > *L*_cat,*α*_) indicates a gap in learning from coordinate information relative to category information. **c**, The mean *L*_coord,*α*_ and *L*_cat,*α*_ across all datasets for transformer-based (red), GNN-based (COGN^15^, blue) and feature-based (MODNet^16^, gray) architectures. A positive slope indicates a larger CC-cliff. Source data

Using these datasets, we follow established property prediction procedures^5,13^ and fine-tune BERT^14^ models to predict the target values for all versions of the dataset (each with a different *α* value). To quantify how well the models learn from each type of information, we compute two aggregate error metrics: the coordinate contribution (*L*_coord,*α*_) and the category contribution (*L*_cat,*α*_). As illustrated in Fig. 2a, *L*_coord,*α*_ measures the model’s error on tasks dominated by coordinate information (that is, for *α* values close to 0), while *L*_cat,*α*_ measures the error on tasks dominated by category information (that is, for *α* values close to 1). We give more examples on how to interpret the curves in ‘CC-cliff’ in the Methods. If *L*_coord,*α*_ > *L*_cat,*α*_, we call this phenomenon the coordinate–category cliff (CC-cliff)—the systematic underperformance on tasks requiring coordinate information.

Our key finding, shown in Fig. 2b, is that, for language models, *L*_coord,*α*_ is consistently larger than *L*_cat,*α*_ across all datasets. We find that language models consistently perform much worse in learning from coordinate information than from category information. This is consistent across different datasets (which are samples from different distributions of coordinate–category configurations). For the purpose of quantifying this metric, we can compute CC-cliff as *L*_coord,*α*_ − *L*_cat,*α*_. A CC-cliff of zero would indicate equal performance, while a positive gap reveals a bias against learning from coordinate data. These findings indicate that language models might not be the best choice for all problem settings.

To contextualize these findings across model classes, Fig. 2c shows the coordinate and category contributions averaged across all datasets for three architectures. The language modeling approach exhibits a large positive slope—low category contribution and high coordinate contribution. A graph modeling approach based on COGN^15^ shows the opposite pattern: a negative slope, indicating that the GNN handles coordinate-dominated tasks comparatively easier than category-dominated ones, reflecting its geometric inductive bias. The descriptor-based model MODNet^16^ lies near zero, exhibiting balanced sensitivity to both information types. This comparison directly illustrates that the CC-cliff is not an inherent property of the task, but a consequence of model architecture and inductive bias (see Supplementary Section 6.4 for more discussion).

### Comparison with n-gram models

To better understand the source of those problems, we performed the same experiments with n-gram models. This experiment is based on the fact that it has been shown that some behaviors of LLMs, such as how they use their context, can be explained by thinking of them as n-gram models^17^.

In addition, we changed the complexity of the learning task^18^ by binning the pairwise distance in coordinate contribution to energy (*E*_coordinate_)—modeling the task as an increasingly complex multiclass classification problem (with the large number of bins approaching the regression problem; see ‘n-gram comparison and task complexity variation’ in the Methods for more methodological details).

**Fig. 3:**
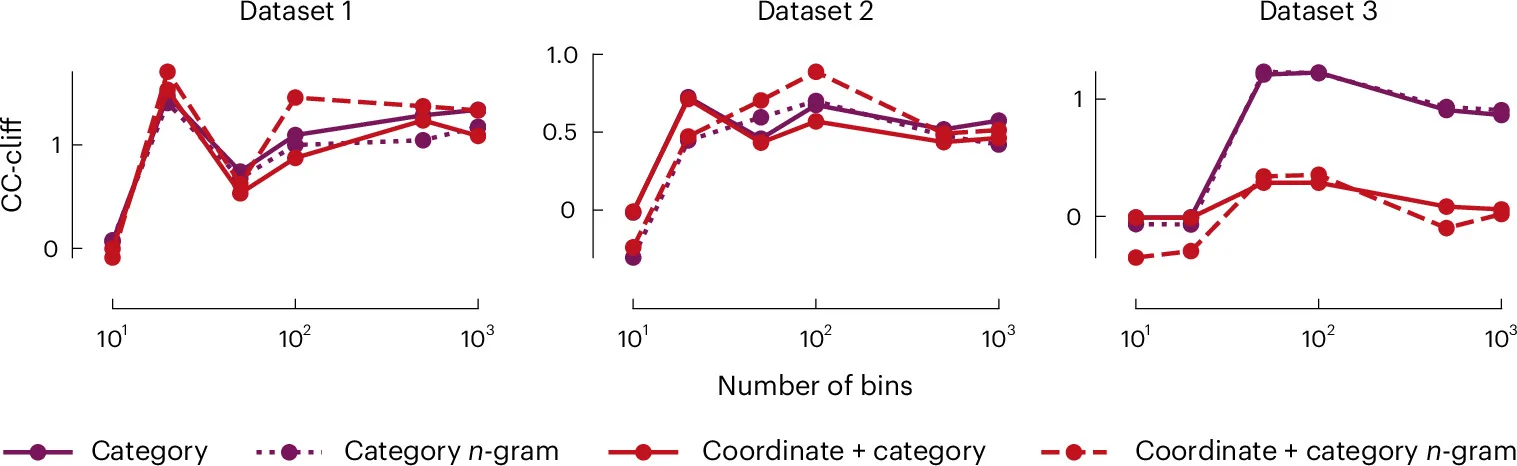
CC-cliff in hypothetical potential energy prediction as a function of binning the potential across different datasets. An illustration of the CC-cliff for models tasked with predicting hypothetical potential energy. The analysis is performed across three distinct datasets. The CC-cliff is plotted against the number of bins used to discretize the pairwise distance (logarithmic scale). Fewer bins correspond to a coarser, generally easier potential energy prediction task for the structures within that dataset, while a higher number of bins represents a finer-grained, more challenging prediction task. Each line corresponds to a different text input (with different levels of information) used to describe the data points in the dataset: categorical (purple), as well as coordinate and categorical (red). Solid lines indicate transformer-based models, and dotted lines represent their n-gram counterparts. The plots show an almost identical behavior of language models to that of n-gram models suggesting that LLMs inherit the properties of n-gram models for tasks involving coordinate and category data. Source data

In Fig. 3, we find that for all datasets we analyzed, the behavior of n-gram models closely follows that of the LLMs fine-tuned on only category or full category and coordinate information. This showcases that, for this task, the behavior we observe can be approximated with n-grams. This understanding is very instructive because for n-gram models we do not expect to be able to predict properties that depend on positional information, as we would need combinatorially many samples to describe such data with n-gram models. For datasets 1 and 2, the two solid lines remain close across all bin resolutions, indicating that the LLM effectively ignores coordinate information regardless of its presence in the input. Dataset 3, which is a more structurally constrained dataset compared with 1 and 2, shows the expected behavior: at low bin resolution, composition alone approximates the coordinate-derived target, but as bins increase and finer geometric resolution is required, this approximation breaks down and the CC-cliff rises for the category-only model. We also note that the binning process affects only the target labels; the text inputs to the LLMs remain unchanged across all bin resolutions.

Our findings from the attention analysis reveal that coordinate information is not leveraged by the models while making predictions (more details are shown in Supplementary Section 4). Taken together, this provides compelling evidence for LLM’s failure to properly account for coordinate information.

### Implications for material property predictions

**Fig. 4:**
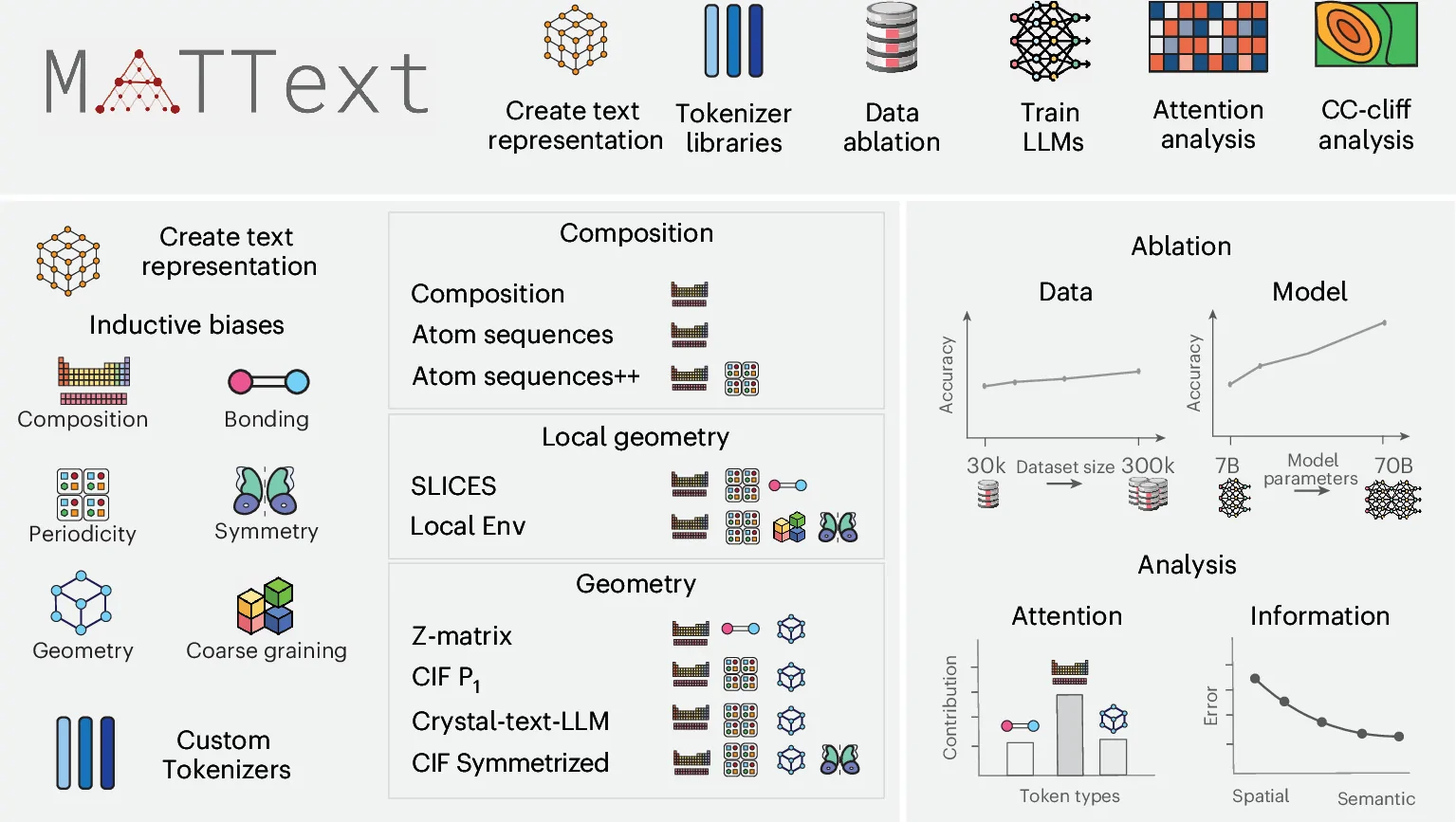
MatText: a holistic platform for end-to-end language modeling of materials, encompassing representation creation, model training and streamlined analysis of results. MatText enables the creation of text representations of crystal structures, offering nine different representations, each with distinct inductive biases. These inductive biases explicitly encode diverse types of information, such as bonding, periodicity, symmetry and others. The framework also supports various tokenization methods, such as atom-level and representation-specific tokenization, as well as different ways to tokenize numbers. MatText facilitates pretraining and fine-tuning of both causal and masked language models, and features modules for scaling-up model and data sizes. In addition, MatText provides tools for analyzing attention mechanisms, assessing the contribution of tokens in predictions on the basis of attention scores, and performing CC-cliff analysis using hypothetical potentials.

Those findings have concrete practical implications for material property prediction and various other machine learning tasks. To investigate these implications, we built an open source framework, MatText, with which we can represent materials in various text forms and seamlessly test the performance of different language models on different materials tasks (Fig. 4). To avoid results being confounded by a special representation, model architecture, tokenizer or dataset choice, we designed targeted experiments to understand the effects of all of those elements.

#### Representation

One of the most important factors in determining the performance of models in materials science is the data representation. The development of text-based representations for materials is an active area of research, and there exists no universally accepted ‘best’ text-based representation for materials (more details on representations are described in Supplementary Section 1.1). With the aim of providing a broad collection in MatText, we implemented many previously proposed text representations, and developed several additional representations to probe a comprehensive set of inductive biases. Thus, the representations in MatText differ not only in the way they present the information in text but also in the information they contain (Fig. 4). Some, such as SLICES ^19^, only contain information about the composition and bonds. Others, such as CIF Symmetrized, contain information about the position of all atoms, in addition to unit cell and symmetry information. A full description of all representations we implemented can be found in Supplementary Section 2.

**Fig. 5:**
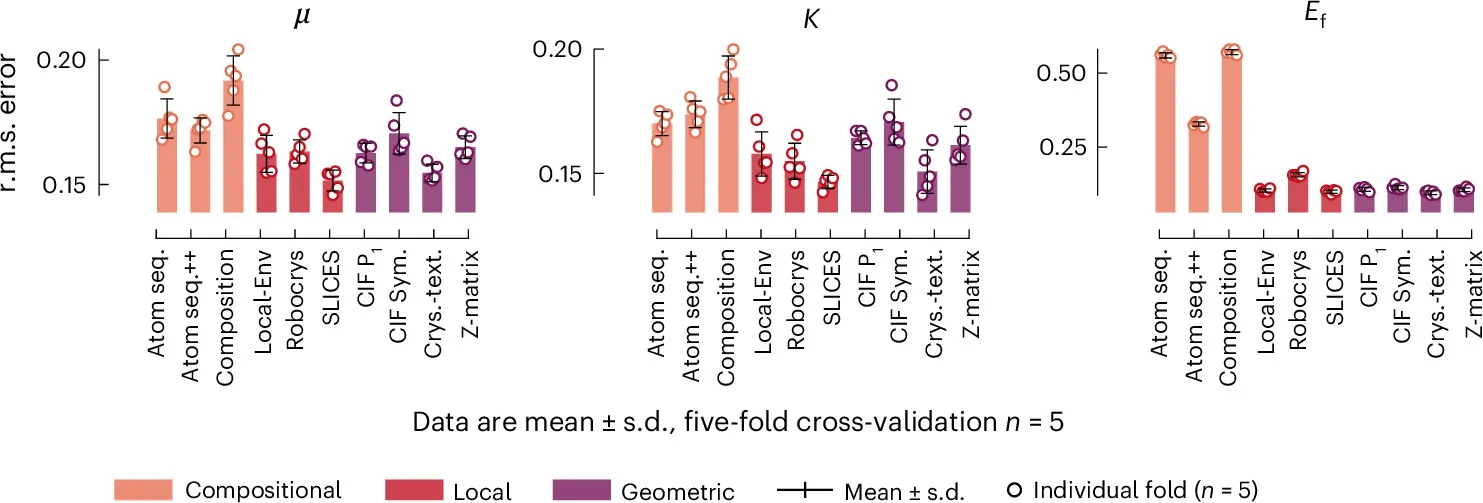
Predictive performance of LLMs in material property prediction tasks (shear modulus (*μ*), bulk modulus (*K*) and perovskite formation energy (*E*_*f*_)) for different representations. Performance is measured by r.m.s. error, with lower values indicating better performance. Representations are grouped by type: compositional-based (orange), local environment-based (red) and geometry-based (purple). Across all three properties, local environment-based representations generally achieve the best performance, while explicit geometric representations show limited improvement or even degraded performance. Notably, the SLICES representation, which lacks explicit coordinate information, performs comparably to geometry-aware representations such as CIF P_1_, suggesting that current LLMs do not effectively leverage explicit coordinate information for material property prediction. Robocrystallographer, which encodes local coordination environments in natural language rather than explicit coordinates, performs comparably to SLICES and Local-Env. Bars show mean r.m.s. error; error bars indicate ±1 s.d. Individual data points from each of the five cross-validation folds are overlaid as circles (data are mean ± s.d., *n* = 5, fivefold cross-validation). A notable exception is the perovskites dataset, where there is a big difference between representations. This dataset has few unique chemical environments compared with the shear and bulk modulus datasets (Supplementary Fig. 6b). Therefore, most of the variance cannot be explained using composition information alone. Atom seq., atom sequence; Atom seq.++, Atom sequence++; CIF Sym., CIF Symmetrized; Cyrs. -text., Crystal-Text-LLM. Source data

In Fig. 5 we show the predictive performance on material property prediction tasks as a function of the input representation. We can make an interesting observation: in many cases, the addition of information does not improve the model performance. For instance, we observe SLICES performing better than CIF P_1_ for all properties, while Local-Env shows comparable performance in the prediction of bulk modulus and shear modulus. These results support our observation that coordinate information cannot be effectively used by LLMs. Supplementary Section 5 shows that these findings are not specific to the choice of tokenizers or how we tokenize numbers.

#### Scale

**Fig. 6:**
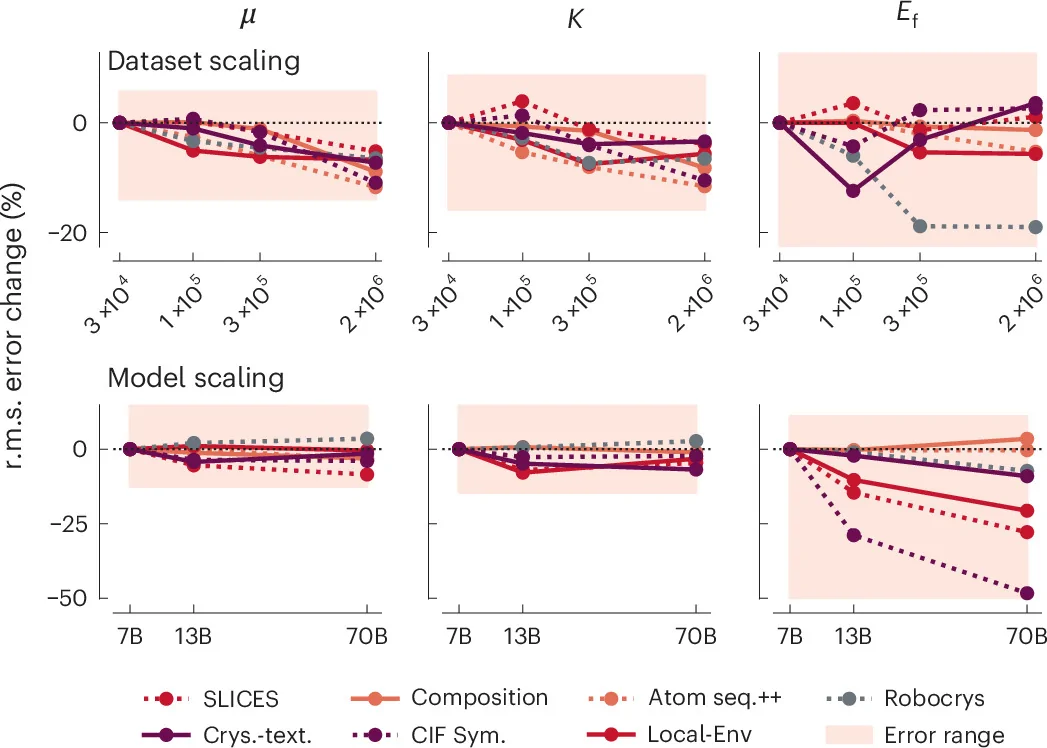
Impact of dataset and model scaling on predictive performance of material representations. The percentage change in r.m.s. error relative to a baseline for various material representations when scaling the pretraining dataset sizes for BERT^14^ models (top row, 3 × 10^4^, 1 × 10^5^, 3 × 10^5^ and 2 × 10^6^ structures) and the language model parameter count (7 billion (B), 13B and 70B parameters) for the instruction-tuned Llama^39^ model (bottom row). Each column corresponds to a different material property: shear modulus (*μ*), bulk modulus (*K*) and formation energy (*E*_*f*_). Each colored line tracks the mean r.m.s. error percentage change for a specific material representation as either dataset size or model size increases. The baseline for dataset scaling is the mean r.m.s. error achieved with 30,000 structures for that representation; for model scaling, the baseline is the mean r.m.s. error of the 7B parameter model. Negative percentage changes indicate improved predictive performance compared with the respective baseline. The light-red shaded region in each subplot defines the global range of r.m.s. error percentage changes across all plotted representations and scaling points. Within each color group (compositional, orange; local, red; and geometric, purple), different line styles (solid and dotted) distinguish individual representations. Robocrystallographer (Robocrys) is highlighted in gray, in addition to being a local representation, it encodes structural information as natural language descriptions rather than explicit coordinates. The dotted black line is a reference to ‘no change’. Source data

One of the most widely established ways of improving model performance is by scaling-up—meaning to train larger models on more data^20,21^—which has been proposed in general language modeling, as well as in scientific domains including chemistry^22^. While there have been some inconsistencies^23^, the general observation on many tasks has been that scale reliably improves performance—a pattern that has been formalized with empirical scaling laws^21^. To analyze if the performance in predicting material properties can be improved by scaling, we ablated dataset size and model parameter count. We evaluated all our experiments, including those with larger model and data scales, in a fivefold cross-validation setting to measure variance. We present a comprehensive benchmarking analysis leveraging more than 2,000 language model training jobs. Figure 6 showcases our findings: we show the impact of dataset scaling in the top row and model size scaling in the bottom row. In all plots, we measure the change in root mean square error (r.m.s. error) with respect to the starting configuration. We indicate the range between the biggest and smallest change in r.m.s. error with a colored rectangle—any point within this range is not distinguishable from a random fluctuation within our experimental setting.

**Extended Data Fig. 1:**
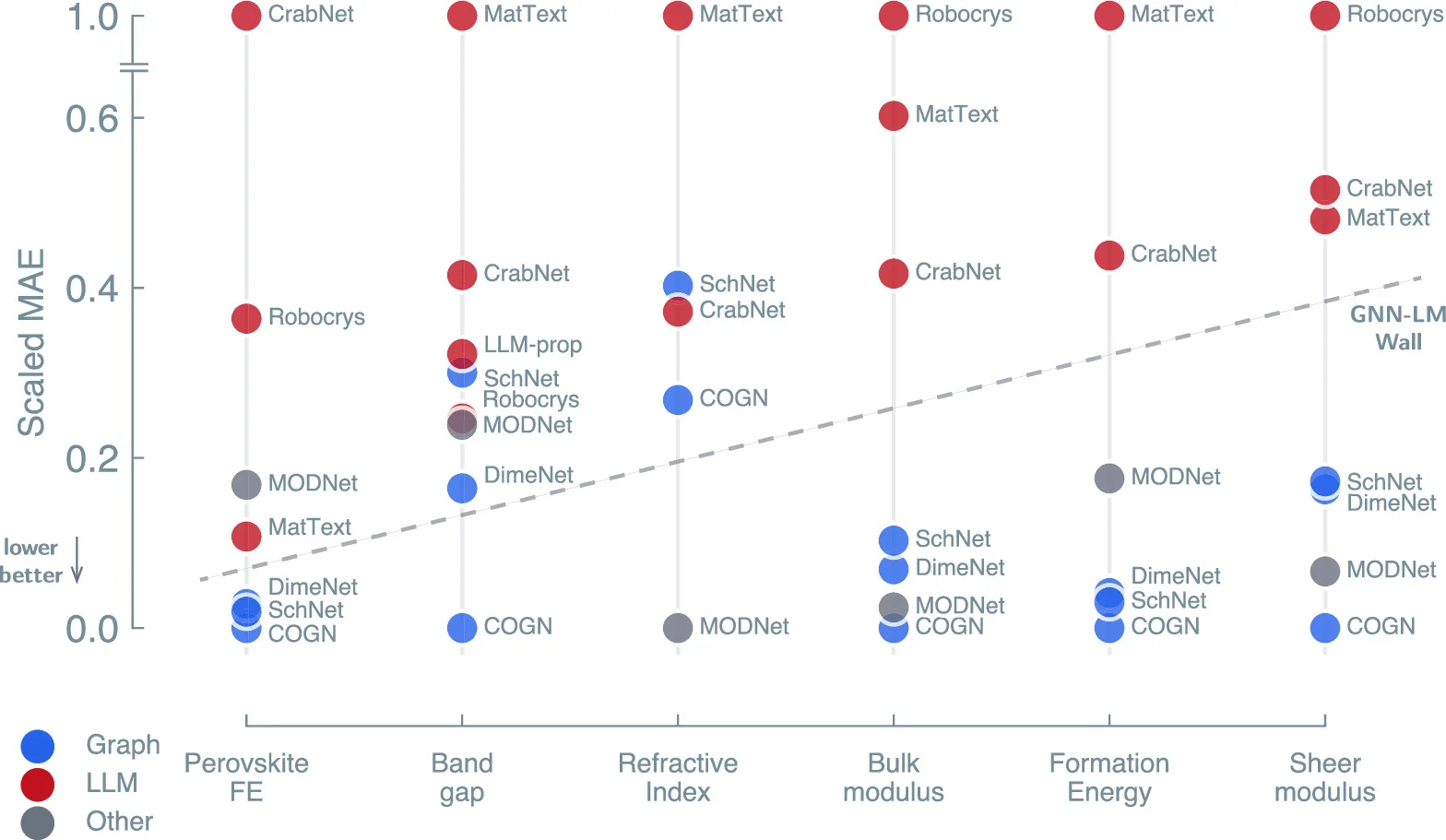
Comparison of the performance on language and graph-based models on materials property prediction tasks. The figure presents a comparative performance analysis, measured by scaled MAE, between graph-based models (bluecircles) and language-based models (redcircles) across six different material property prediction tasks. The dashed gray line, labeled “GNN-LM wall,” serves as a visual guide to generally demarcate the performance difference between the two classes of models. GNN models (shown in blue) consistently achieve better performance across all properties, while LLM models (shown in red) generally exhibit higher errors. This systematic performance gap suggests that current GNN architectures are more effective at learning and predicting materials properties compared to LLM-based approaches. Shown in blue are GNN-based approaches (COGN, SchNet, DimeNet) and in red are approaches based on language models (LLM-prop, CrabNet, Robocrys). MatText corresponds to the best-performing BERT-based model pretrained on 2M structures with the representation selected separately for each property. Additionally, MODNet is included, which is a descriptor-based approach distinct from both GNNs and LLMs. Source data

We find that, for all representations and prediction targets we investigated, dataset scaling (Fig. 6, top row) yields minimal changes in r.m.s. error (less than 20%, for example, an absolute shift of <0.04 for a baseline r.m.s. error of 0.2). For model scaling (Fig. 6, bottom row), changes are similarly modest for *μ* and *K*. For both scaling approaches, *E*_*f*_ shows larger reductions, around ~20% with Robocrystallographer representation in data scaling, and up to ~50% for some geometric representations in model scaling, although these are not consistent across representations. Importantly, even accounting for these improvements, the absolute performance of LLMs remains far below GNN-based approaches (Extended Data Fig. 1). Our findings indicate that increasing data scale or model size is not sufficient to substantially improve the modeling of material properties using LLMs.

#### The GNN-LM wall

Given our findings and the recent surge of attempts of using LLMs for (material) property predictions, we analyzed the commonly used MatBench leaderboard^24^ to further understand relevant consistencies and differences. In Extended Data Fig. 1, we show the property-wise min–max scaled Mean Absolute Error (MAE) across the selected approaches, where 0 represents the best performance (lowest MAE), 1 the worst performance (highest MAE), and intermediate values indicate relative performance within the observed range. We distinguish different modeling paradigms with colors: blue showing approaches relying on GNNs (COGN^15^, SchNet^25^ and DimeNet^26^) and red showing approaches based on language models (LLM-prop-^6^, CrabNet^27^ and Robocrys^28^).

Notably, we consistently find language modeling-based approaches at the top of the plot, indicating the highest error and lowest performance. In addition, we find a striking segregation, where one can almost draw a line separating GNN-based approaches from language modeling-based ones. This observation underscores our findings that certain properties—especially if they depend on coordinate information (for example, mechanical properties)—cannot efficiently be modeled with current language modeling approaches: independent of representation, model size and dataset size. In practice, more fundamental changes to the architecture of language models are needed to make it possible to model coordinate information with greater effectiveness. Alternatively, researchers can already rely on model architectures that are optimized to deal with such data.

## Discussion

Predicting properties from coordinate–category data—whether atomic positions in materials, molecular coordinates in chemistry, or feature vectors in machine learning—represents a fundamental challenge across the physical sciences. The promise of LLMs has captivated researchers with their apparent universality: a single architecture that learns from text alone, yet seemingly grasps complex patterns across domains. This vision has driven a surge of attempts to deploy LLMs for scientific property prediction, with researchers racing to encode crystal structures, molecules and other vectorial data into text, hoping to unlock the same transformative capabilities witnessed in natural language processing.

Our systematic investigation reveals a more sobering reality. Through controlled experiments with synthetic datasets where we can precisely tune the balance between category and coordinate information, we uncover a fundamental limitation: LLMs systematically fail to learn from coordinate information in coordinate–category data. This failure persists across architectures, scales and training strategies. Even massive increases in data and model parameters—the traditional levers of improvement in language modeling—yield no meaningful gains. LLMs behave remarkably like n-gram statistics, capturing categorical patterns while remaining blind to the spatial arrangements that often determine physical properties.

Yet, this apparent failure points to a path forward. By delineating where LLMs excel (learning from categorical information such as element types) and where they struggle (extracting geometric relationships), we provide a framework for making informed modeling choices. Our findings suggest that rather than pursuing LLMs as universal replacements for specialized architectures, the field should embrace a more nuanced approach: deploying LLMs for tasks dominated by compositional patterns while reserving geometric neural networks and other purpose-built architectures for problems requiring spatial reasoning.

In an era rushing toward universal models, we demonstrate that understanding the inductive biases of our tools remains as crucial as ever. The future of scientific machine learning might therefore not lie in finding one universal model to rule them all, but in matching the right architecture to the right problem.

### Limitations

The experimental scope covers three properties (shear modulus, bulk modulus and perovskite formation energy) from a limited set of benchmark datasets, which may not be representative of the full diversity of material property prediction tasks. Only encoder-based (BERT) and decoder-based (Llama-2) transformer architectures were evaluated; multimodal models that fuse text with explicit coordinate embeddings, or architectures with geometric priors built into the tokenization, were not investigated. The scaling study was limited to models up to 70B parameters using parameter-efficient fine-tuning; whether full fine-tuning or pretraining from scratch on coordinate-rich corpora would alter the observed scaling behavior remains an open question.

### Future directions

Applying the CC-cliff framework to molecular property prediction—for example, predicting quantum chemical properties from representations with and without explicit 3D coordinates—and to biological sequence–structure tasks would test whether the coordinate–category gap generalizes across physical sciences domains beyond crystalline materials. Coordinate-aware tokenization schemes, in which atomic positions are encoded as learned continuous embeddings, rather than discretized text tokens, would directly test whether the limitation is intrinsic to transformer architectures or specific to their text-based interface. Causal intervention studies—for example, activation patching to identify which layers suppress coordinate information—would provide a mechanistic complement to the statistical observations reported here.

## Methods

### Modeling coordinate information with transformers

Benchmarking with real-world systems can be confounded by inherent correlations within the training data. For example, certain compositions might predominantly form specific structural motifs or crystallize in particular space groups in materials. A model could leverage these statistical correlations to predict a structure-dependent property accurately, even with a limited understanding of the actual geometric arrangements, by primarily relying on compositional cues. To illustrate, if a specific elemental combination almost exclusively forms a known ground state structure, a model might appear to ‘understand’ the geometry leading to this ground state by simply recognizing the composition. This makes it challenging to isolate and quantify a model’s proficiency in processing coordinate information. To address this, we designed hypothetical potentials, where the ‘ground truth’ target values can be continuously tuned to depend more or less on categorical versus positional features. This allows for a systematic probing of a model’s learning capabilities across this spectrum.

#### The physics-inspired hypothetical potential

We consider a system characterized by a collection of *N* entities, where each entity *k* possesses a category type (for example, discrete type) and a continuous coordinate attribute **r**_*k*_ (for example, position). We then define a scalar target value, *E*, analogous to an energy, for each system configuration. This label is a linear interpolation between a purely category term (*E*_category_) and a purely coordinate term (*E*_coordinate_), controlled by a mixing parameter *α* ∈ [0,1]

2 $$E(\alpha) = \alpha E_{\mathrm{category}} + (1 - \alpha) E_{\mathrm{coordinate}}$$

The contribution from category related information, *E*_category_, is defined as a sum over the counts of each entity type

3 $$E_{\mathrm{category}} = \sum_{k=1}^{N_t} w_k n_k$$

where *N*_*t*_ is the number of unique entity types, *n*_*k*_ is the count of entities of type *k*, and *w*_*k*_ is a weight associated with particles of type *k* (in our calculations, we map chemical elements to their energy parameters from the Universal Force Field^29^). This term represents the contribution of the intrinsic properties of each type of particle. The *E*_category_ term captures contributions based solely on the ‘what’ and ‘how many’ of the entities.

The contribution of coordinate-related information, *E*_coordinate_, is designed to depend on the vectors and their relative arrangements

4 $$E_{\mathrm{coordinate}} = \sum_{i=1}^{N} \sum_{j \in \mathcal{N}(i)} V \left( \left| \mathbf{r}_i - \mathbf{r}_j \right| \right)$$

where *V*(∣**r**_*i*_ − **r**_*j*_∣) represents a pairwise interaction potential (for instance, Lennard–Jones in our experiments) dependent on the distance between entity *i* and its neighbors *j* within a defined neighborhood ${\mathscr{ \mathcal N }}(i)$. This term captures contributions on the basis of the ‘where’ and ‘how arranged’ of the entities.

By varying the mixing parameter *α* ∈ [0,1], we can continuously tune the nature of the generated target values. In one extreme, at *α* = 0, the target value *E* becomes solely dependent on coordinate information (*E* = *E*_coordinate_). Conversely, at the other extreme, *α* = 1, the target value depends exclusively on category information (*E* = *E*_category_). Intermediate values of *α*, such as *α* = 0.5, yield labels that represent a balanced contribution from both positional and category terms, while other values within the (0, 1) interval enable a spectrum of relative influences between these two components. Before mixing, both *E*_category_ and *E*_coordinate_ are independently standardized to zero mean and unit variance across the dataset, ensuring that *α* controls the relative information contribution rather than differences in raw energy scales. This setup, while a simplification of complex real-world interactions, provides a controlled environment to assess how well a model learns from these distinct information modalities.

#### CC-cliff

To quantify the model’s relative ability to learn from coordinate versus category information, we define two aggregate metrics: the coordinate contribution (*L*_coord,*α*_) and the category contribution (*L*_cat,*α*_). These scores quantify the model’s performance drop in regimes dominated by either coordinate or category information, relative to its performance in a balanced scenario (*α* = 0.5).

The coordinate contribution (*L*_coord,*α*_) is calculated by summing the losses for *α* values where coordinate information dominates, and subtracting a baseline derived from the loss at *α* = 0.5

5 $${L}_{\mathrm{coord},\alpha }=\left(\sum _{\alpha \in {\alpha }_{g}}\,\mathrm{loss}(\alpha )\right)-| {\alpha }_{g}|\times\mathrm{loss}\,(0.5),$$

where *α*_*g*_ = {0, 0.2, 0.4} is the set of *α* values biased toward coordinate information, and ∣*α*_*g*_∣ = 3 is the number of points in this set.

Similarly, the category contribution (*L*_cat,*α*_) is calculated for *α* values where category information dominates

6 $${L}_{\mathrm{cat},\alpha }=\left(\sum _{\alpha \in {\alpha }_{c}}\,\mathrm{loss}(\alpha )\right)-| {\alpha }_{c}|\times\mathrm{loss}(0.5),$$

where *α*_*c*_ = {0.6, 0.8, 1.0} is the set of *α* values biased toward category information, and ∣*α*_*c*_∣ = 3.

A higher contribution (for example, a large positive *L*_coord,*α*_) indicates that the model struggles more in that respective regime (coordinate-dominant for *L*_coord,*α*_) compared with the balanced *α* = 0.5 case. Conversely, a score near zero suggests performance comparable to the balanced case for that regime. The CC-cliff is then analyzed by comparing *L*_coord,*α*_ and *L*_cat,*α*_. For instance, in Fig. 2, *L*_coord,*α*_ and *L*_cat,*α*_ are plotted against each other.

- If *L*_coord,*α*_ ≫ *L*_cat,*α*_, the model finds it substantially harder to learn from coordinate information than from category information, relative to the *α* = 0.5 baseline. If *L*_cat,*α*_ ≫ *L*_coord,*α*_, the converse is true.
- If *L*_coord,*α*_ ≈ *L*_cat,*α*_, the model exhibits a similar level of difficulty (or ease) in both regimes relative to the baseline.

This quantitative framework, based on systematically varying the nature of the learning task, enables a more nuanced assessment of an LLM’s capabilities beyond performance on a single, fixed task. It specifically probes the model’s ability for processing and integrating different fundamental types of information present in vector-based descriptions of systems.

### n-gram comparison and task complexity variation

Given that our task involves predicting target values on the basis of textual inputs, comparing LLM performance to that of n-gram models provides an informative baseline.^17^ Intuitively, n-gram models are not expected to capture complex, nonlocal geometric dependencies or perform intricate calculations (such as those in equation (4)) without an exponentially large number of samples to cover the combinatorial space of token sequences representing these structures. For instance, to accurately model pairwise interactions using only local n-grams, a purely compositional vocabulary might contain around 128 tokens (for elements and digits). However, to also encode atomic positions with a modest 0.1 Å resolution across a 20 Å cell, each atom’s position adds 8 million (8 × 10^6^) possible location identifiers. This inflates the vocabulary of ‘atom-at-a-position’ tokens to over a billion (>10^9^). Learning a simple pairwise interaction (a 2-gram) would require sampling from a space of possibilities that is ${(8\times 1{0}^{6})}^{2}\approx 6.4\times 1{0}^{13}$ times larger than in the compositional-only case. Learning physical laws this way is thus exceptionally sample-inefficient, as it would require an astronomical amount of data to observe a meaningful fraction of relevant atomic pairings.

If LLMs show similar performance trends to n-gram models on this task, it might suggest they are relying on more superficial textual patterns rather than developing a deeper, generalizable understanding of the underlying relationships.

Concurrently, we investigate how the models’ learning capabilities are affected by the ‘resolution of the geometric information’. The calculation of target values, even in our simplified potential (equation (2)), involves operations on continuous vector attributes, such as the pairwise distances ∣**r**_*i*_ − **r**_*j*_∣ used in *E*_coordinate_. These operations might not align perfectly with a transformer’s natural computational style (cf. RASP conjecture^18^), potentially leading the model to learn shortcuts or struggle with precise generalization, especially when the input data (for example, textual representation of crystal structures) requires complex parsing algorithms to extract these from representations of varying length.

#### Task complexity variation via pairwise distance binning

To probe this, we modify the generation of the target labels *E*(*α*) by discretizing the pairwise distances ∣**r**_*i*_ − **r**_*j*_∣ into a varying number of bins (*M*) before they are used to calculate *E*_coordinate_ (equation (4)). This effectively changes the ‘physical’ model whose properties the LLM must learn:

- When *M* is small (coarse distance binning), the *E*_coordinate_ term, and consequently *E*(*α*), is derived from a simplified geometric landscape where only gross differences in inter-entity distances matter. The learning task involves predicting target values from a system with inherently lower geometric resolution.
- When *M* is large (fine distance binning), *E*_coordinate_ and *E*(*α*) are derived from a system that is sensitive to subtle variations in distances, more closely approximating a potential based on continuous geometric inputs.

This allows us to assess whether the models are capable of learning fine-grained geometric dependencies when the target values are sensitive to them, or if their performance degrades, potentially indicating an inability to extract or utilize high-resolution continuous information from the textual input, or an over-reliance on coarser patterns. This approach helps to distinguish between a model’s ability to learn a complex function versus its ability to learn a function defined by high-resolution input features.

#### n-gram model implementation

For the n-gram model experiments, the input system descriptions (for example, CIF files), similar to that used for the LLMs, were used to generate features. Specifically, we extracted term frequency–inverse document frequency^30^ weighted features from unigrams and bigrams derived from this textual data. An XGBoost^31^ classifier was then trained on these term frequency–inverse document frequency features. The target for these n-gram models was the energy target values, consistent with the task given to the LLMs in the binned experiments described above. The model performance was evaluated using standard classification metrics appropriate for the multi-class problem.

### MatText Python package

While tools such as Robocrystallographer ^32^ and MOFid^33^ exist for specific purposes, no unified framework has been available to systematically derive and benchmark multiple text representations across a broad range of crystalline materials. MatText provides an object-oriented way to convert crystal structures into text representations. Each of the text representations has different information content (going from just composition information to information about the composition and the position of all the atoms) (Fig. 4), allowing us to analyze what information language models can use for material property predictions. In addition, the representations feature different combinations of inductive biases, thereby enabling us to identify the most meaningful ones in our analysis. For analysis purposes in this Article, we have categorized representations into broadly three categories: composition-based representations (composition, atom sequence and atom sequence++), representations that focus on local geometry (SLICES and Local-Env) and representations that capture 3D geometry (CIF P_1_, CIF Symmetrized, Z-Matrix and Crystal-Text-LLM). More details about inductive biases and various representations with examples are provided in Supplementary Sections 1.2 and 2, respectively.

### Scaling experiments

To systematically investigate the impact of scale on performance, we conducted experiments along two primary axes: dataset size and model parameter count. First, we explored data scaling by pretraining BERT models from scratch on datasets of progressively larger sizes. Second, we examined model scaling by applying parameter-efficient fine-tuning to pre-existing Llama-2 models of different scales. The specific setups for these two experimental tracks are detailed below.

#### Scaling dataset sizes

We created pretraining datasets of four different sizes for all representations shown in Fig. 4. The pretraining datasets are derived from the NOMAD^34^ database, queried via scripts from MatSciML^35^ and filtered for 3D crystal structures for which geometry optimization was performed using the PBE functional and the VASP code. Duplicate structures were removed on the basis of CIF symmetry. From the resulting pool of ~2 × 10^6^ unique structures, we randomly sampled subsets of 3 × 10^4^, 1 × 10^5^, 3 × 10^5^ and 2 × 10^6^ structures. For each subset, all nine text representations were derived using the MatText framework, yielding 36 pretraining datasets in total.

We pretrained a separate base BERT model for all these datasets, yielding 36 base BERT models, which were then fine-tuned on different properties from matminer^36^ (particularly on formation energy of perovskite structures^37^, bulk modulus^38^ and shear modulus^38^, enabling us to compare with state-of-the-art models in MatBench).

#### Scaling model size

To assess the influence of model scale, we performed parameter-efficient fine-tuning of LLMs of different sizes, specifically, Llama-2 7B, Llama-2 13B and Llama-2 70B models.^39^ We fine-tune the LLM models with low-rank adaptation (LoRA)^40^. Packing was disabled, and prompt tokens were masked in the loss so that only completion tokens contribute to training. In addition to LoRA, we use 4-bit quantization with nf4 quantization type and float16 compute data type.^41^

#### Hyperparameters

##### Pretraining

We chose a batch size and context length specific to each representation (given in Supplementary Table 2) and trained for a total of 50 epochs with a learning rate of 2 × 10^−4^, using a masked language modeling approach, with a masking probability of 0.15.

##### Fine-tuning

For fine-tuning, we employ early stopping with a patience of 10 and a threshold of 0.001 to prevent overfitting, utilizing 20% of the data for evaluation while the remaining 80% is used for training. The learning rate is set to 2 × 10^−4^. The pretrained base model layers are not frozen, and a regressor head on top of the base model is used for the regression where the embedding of the first token ([CLS] token) is used as the feature for BERT models. All fine-tuning and evaluation were done in a fivefold cross-validation setting.

Following related work^13,42^ for Llama fine-tuning, we employed a rank-size of 32 and *α*_LoRA_ = 64, with a batch size of eight for 5 epochs and a cosine-annealed learning rate, with no bias applied on a CAUSAL_LM task. We accumulated gradients over four steps and employed gradient checkpointing. The learning rate was set to 3 × 10^−4^ with a cosine scheduler, a warm-up ratio of 0.03 and a weight decay of 0.001. Optimization was performed using the paged_adamw_32bit optimizer. A maximum gradient norm of 0.3 was maintained to ensure stable fine-tuning.

Owing to computational limitations, a comprehensive hyperparameter optimization was infeasible. However, for the Llama fine-tuning, we performed some experimentation with the LoRA rank, learning rate and warm-up ratio. We found no meaningful performance difference for rank and learning rate (with results falling within the fivefold cross-validation error bars). The warm-up ratio, however, was more sensitive, as smaller warm-up steps resulted in more invalid generations. All hyperparameter initial values were informed by those calculations^13,42^, and we observed no trends that suggest that different parameters would alter the effects seen in the experiments.

## Supplementary information

Supplementary Information Supplementary Figs. 1–8 and Tables 1 and 2.

Peer Review File

Supplementary Data 1 Statistical source data for Supplementary Fig. 2. r.m.s. error percentage improvement of RT tokenizer versus standard tokenizer per representation, property and dataset size.

Supplementary Data 2 Statistical source data for Supplementary Fig. 3. Coordinate contribution and category contribution values per representation and material property dataset.

Supplementary Data 3 Statistical source data for Supplementary Fig. 5. Raw losses per bin resolution, dataset and representation for *n*-gram and transformer models across all representations.

Supplementary Data 4 Statistical source data for Supplementary Fig. 6. CC-cliff values and structural diversity metrics (convex hull volume, geometric variance) per representation and dataset.

Supplementary Data 5 Statistical source data for Supplementary Fig. 7. Per-dataset values for transformer, COGN and MODNet architectures across all synthetic datasets.

## Source data

Source Data Fig. 2 Source data for CC-cliff analysis (coordinate/category contributions per model and dataset). Per-data-point coordinate contribution and category contribution loss values across all *α* values, datasets and model types (MatText, COGN and MODNet).

Source Data Fig. 3 Source data for *n*-gram binning experiment. Raw loss values per bin resolution, dataset and representation type for *n*-gram and transformer models.

Source Data Fig. 5 Per-fold r.m.s. error values for all ten representations across three properties.

Source Data Fig. 6 r.m.s. error percentage change values for dataset and model scaling experiments (30k–2M) and model scaling (7B–70B).

Source Data Extended Data Fig. 1 Scaled MAE values for GNN-based and LLM-based models across six MatBench property prediction tasks.

## Data Availability

To facilitate the benchmarking and reproducibility of our work, the datasets used in this work are available via Hugging Face^43^. Source data are provided with this paper.

## Code availability

The code for using MatText is released under MIT license with tutorials and documentation and is available via GitHub at https://github.com/lamalab-org/MatText and via Zenodo at https://doi.org/10.5281/zenodo.21151839 (ref. ^44^). Instructions for running the code can be found at https://lamalab-org.github.io/MatText/.
