# Supplementary material for: Less can be more for predicting properties with large language models
Source: Nat Comput Sci. 2026 Oct 12;6(10):1124–32. doi: 10.1038/s43588-026-01045-2 (PMC13686937; doi:10.1038/s43588-026-01045-2)
Supplement: Supplementary file 1 [file 43588_2026_1045_MOESM1_ESM.pdf]

nature computational science

Supplementary information

https://doi.org/10.1038/s43588-026-01045-2

Less can be more for predicting properties with large language models

In the format provided by the authors and unedited

Contents

1 Related works **2**
1.1 Language models for material modeling 2
1.2 Inductive biases for material modeling 2

2 Representations **3**

3 Examples of synthetic data **9**

4 Attention analysis **10**

5 Tokenizer **11**

6 Coordinate-Category Cliff **13**
6.1 Interpretation of CC-Cliff Values 14
6.2 Comparison of Coordinate-Category Cliff (CC-Cliff) with `n-gram` models . 14
6.3 Relationship of CC-Cliff with diversity 16
6.4 CC-Cliff across model architectures 17

1 Related works

1.1 Language models for material modeling

Language modeling has emerged as a promising method for predicting protein structure and function, representing amino acid sequences as text.[1–5] While some research suggests language models capture structural information from sequence data,[6] others find their performance on many downstream tasks does not consistently scale with pretraining.[7] Similarly, text-based representations like SMILES[8] and SELFIES[9,10] have been developed for molecules,[11–16] enabling language modeling for tasks such as synthesis planning,[17,18] property prediction,[19–22] and conditional molecule generation.[23–26]

While protein and molecular text representations offer inspiration, materials science presents unique challenges due to properties depending on 3D structure and periodic repetition,[27] thereby complicating the development textual representations. Nevertheless, various approaches have been proposed, such as Robocrystallographer for human-readable crystal descriptions,[28] used in predictive models,[29–31] and specialized representations like MOFid for specific material classes.[32,33] An alternative strategy for handling coordinate information is to convert it into categorical descriptors via crystallographic symmetry, as in Goodall *et al.*[34], which encodes structures using Wyckoff positions, and De Breuck *et al.*[35], which adopts a similar approach. Such representations effectively discretize geometric information, potentially making it more accessible to language models—a hypothesis supported by the competitive performance of our Local-Env representation, which also employs Wyckoff labels (see Supplementary Section 2).

However, no comprehensive universal representation has emerged for materials, making language modeling in this field substantially more challenging.

1.2 Inductive biases for material modeling

The modeling of physical systems can often benefit from the inclusion of physical background knowledge as inductive bias. Locality, smoothness, and symmetry are the most widely used inductive biases.[36] Locality is commonly incorporated using a distance cutoff and rationalized with the nearsightedness principle of quantum mechanics.[37] Related to this is using coarse-grained molecular motifs as inductive bias.[38,39] Symmetry has been incorporated in many of the most performant models by designing invariant or equivariant features[36,40] or model architectures.[41–45] Previous work has indicated that for certain phenomena (for instance, when all structures in a dataset are in the ground state), composition might implicitly encode geometric information.[46–48]

2 Representations

MatText encompasses nine distinct text-based representations for material systems, including representations developed in this work. Each representation incorporates unique inductive biases that capture relevant information and integrate prior physical knowledge about materials. Supplementary Section 2 summarizes the inductive biases in each representation.

Supplementary Table 1: MatText Representations considered in this work and the inductive biases they encode. Representations are classified into three broader categories based on the information it encodes.

Representation	Stoichiometry	Bonding	Geometry	Symmetry	Periodicity	Coarse-Graining
**Composition Representations**						
Composition	✓					
Atom Sequence	✓					
Atom Sequence++	✓				✓	
**Local-Geometry Representations**						
SLICES	✓	✓			✓	
Local-Env	✓	✓		✓		✓
**Geometry Representations**						
Crystal-Text-LLM	✓		✓		✓	
CIF P_1	✓	✓	✓		✓	
CIF Symmetrized	✓		✓	✓	✓	

Composition represents the most basic level of material description, providing only the stoichiometric formula that indicates which elements are present and their relative ratios. Prior work has shown that in certain cases, material composition alone can be predictive for various materials properties.[46] Hence, we also consider the composition in customary Hill notation.[49]

Supplementary Box 1: Composition

- Li7La3Zr2O12

Atom Sequence To investigate the effect of the representation of compositional information, we explicitly list all the atoms present within the unit cell to eliminate any confusion that might arise from interpreting numbers as stoichiometric coefficients. Concretely, structures are represented by listing each atom symbol n times to denote repetition within

the unit cell structure. This representation is an intermediate representation between composition and representation containing information related to bonding or periodicity.

Supplementary Box 2: Atom Sequence

- Li Li Li Li Li Li Li La La La Zr Zr O O O O O O O O O O O O

Atom Sequence++ We incorporate lattice parameters sequentially into the Atom Sequence to ablate the effect of having unit cell dimensions. This representation bridges the gap between purely compositional and fully structural descriptions. Although the position of atoms is not available, it contains information related to periodicity.

Supplementary Box 3: Atom Sequence++

- Li Li Li Li Li Li Li La La La Zr Zr O O O O O O O O O O O O 11.3 11.3 11.3 108.3 108.3 111.6

SLICES In addition to composition information, SLICES encompasses the composition and bonding of atoms within and across the unit cell. It is an invariant and invertible string representation without explicit information about the atom coordinates.[50] The representation is a single-line string starting with elemental symbols within the unit cell and followed by bond descriptions in the format `uvxyz`. Here, `u` and `v` represent node indices, while `xyz` denotes the direction of the unit cell necessary to establish each bond connection across the unit cell boundaries.

Supplementary Box 4: SLICES

- Mg Ta Pt 0 2 - - o 0 2 - o - 0 2 - o o 0 2 o - - 0 2 o - o 0 2 o o - 0 1 - - o 0 1 - o - 0 1 o - - 1 2 o o o

Local-Env We also report a new text representation inspired by the frequently used inductive bias of locality and Pauling's rule of parsimony, which states that local environments tend to be redundant.[51] To derive the local environments, we perform the coordination environment analysis reported by Waroquiers *et al.*[52], derive Wyckoff labels using `spglib`,[53] and SMILES using `openbabel`.[54] We prefix the representation using the spacegroup symbol and then list the Wyckoff label and SMILES separated by line breaks for each local environment. This representation features inductive biases related to local geometry.

Supplementary Box 5: Local-Env

- R3m
  Ta (1a) [Ta]#[Pt]
  Pt (1a) [Ta]#[Pt]
  Mg (1a) [Ta][Mg][Ta].[Ta].[Pt].[Pt].[Pt]

Crystal-Text-LLM This representation is a condensed version of the CIF, which includes only the parameters necessary for building the crystal structure[55] (without additional syntax of the CIF). Given the lattice parameters of the unit cell, atom types, and their coordinates, the bulk material structure can be represented as a listing of element symbols and coordinates separated by linebreaks that are prefixed by the list of lattice parameters (cell lengths and angles). Crystal-Text-LLM is part of the representation group incorporating geometry inductive biases (spatial information) but they also have composition information (categorical information).

Supplementary Box 6: Crystal-Text-LLM

- 3.5 4.2 4.4
  90 90 90
  Ta
  0.76 0.12 0.00
  Ta
  0.00 0.12 0.18
  V
  0.00 0.00 0.00
  Ga
  0.76 0.00 0.18

CIF P_1 Crystallographic Information Files (CIFs) are a standard way to archive structural data in crystallography.[56] CIF P_1 represents the full Crystallographic Information File format using the primitive space group P_1. They have been previously used for generating materials by fine-tuning large language model (LLM)[57,58] or pretraining small GPT models.[59] In the CIF P_1 representation, the crystal structure is represented in the lowest symmetry (P_1 space group). This means that if there is any symmetry in the crystal structure, it is not explicitly defined. CIF P_1 is part of the representation group incorporating geometry inductive biases (spatial information) but they also have composition information (categorical information). Contrasting the CIF P_1 and the Crystal-Text-LLM representations allows us to obtain insights into the importance of the compactness of representations.

Supplementary Box 7: CIF P_1

- data_MgTaPt
  _symmetry_space_group_name_H-M 'P 1'
  _cell_length_a 4.32
  _cell_length_b 4.32
  _cell_length_c 4.32
  _cell_angle_alpha 60.0
  _cell_angle_beta 60.0
  _cell_angle_gamma 60.0
  _symmetry_Int_Tables_number 1
  _chemical_formula_structural MgTaPt
  _chemical_formula_sum 'Mg1 Ta1 Pt1'
  _cell_volume 56.85
  _cell_formula_units_Z 1
  loop_
  _symmetry_equiv_pos_site_id
  _symmetry_equiv_pos_as_xyz
  1 'x, y, z'
  loop_
  _atom_site_type_symbol
  _atom_site_label
  _atom_site_symmetry_multiplicity
  _atom_site_fract_x
  _atom_site_fract_y
  _atom_site_fract_z
  _atom_site_occupancy
  Mg Mg0 1 0.0 0.0 0.0 1.0
  Ta Ta2 1 0.58 0.58 0.58 1.0
  Pt Pt1 1 0.53 0.53 0.53 1.0

CIF Symmetrized This CIF representation represents the asymmetric unit and list the symmetry operations that can be applied to fill the unit cell by generating all equivalent positions. It typically contains fewer lines describing atoms' positions than the CIF in P_1 but extra text describing the symmetry operations. Thus, this representation can contain more tokens for some structures than the P_1 variant (CIF P_1). This representation allows us to elucidate the importance of explicit symmetry information alongside positional information. CIF Symmetrized is part of the representation group incorporating geometry inductive biases (spatial information) but they also have composition information (categorical information).

Supplementary Box 8: CIF Symmetrized

- data_MgTaPt
_symmetry_space_group_name_H-M R3m
_cell_length_a 4.32
_cell_length_b 4.32
_cell_length_c 10.57
_cell_angle_alpha 90.0
_cell_angle_beta 90.0
_cell_angle_gamma 120.0
_symmetry_Int_Tables_number 160
_chemical_formula_structural MgTaPt
_chemical_formula_sum 'Mg3 Ta3 Pt3'
_cell_volume 170.55
_cell_formula_units_Z 3
loop_
_symmetry_equiv_pos_site_id
_symmetry_equiv_pos_as_xyz
1 'x, y, z'
2 '-y, x-y, z'
3 '-x+y, -x, z'
4 '-y, -x, z'
5 '-x+y, y, z'
6 'x, x-y, z'
7 'x+1/3, y+2/3, z+2/3'
8 '-y+1/3, x-y+2/3, z+2/3'
9 '-x+y+1/3, -x+2/3, z+2/3'
10 '-y+1/3, -x+2/3, z+2/3'
11 '-x+y+1/3, y+2/3, z+2/3'
12 'x+1/3, x-y+2/3, z+2/3'
13 'x+2/3, y+1/3, z+1/3'
14 '-y+2/3, x-y+1/3, z+1/3'
15 '-x+y+2/3, -x+1/3, z+1/3'
16 '-y+2/3, -x+1/3, z+1/3'
17 '-x+y+2/3, y+1/3, z+1/3'
18 'x+2/3, x-y+1/3, z+1/3'
loop_
_atom_site_type_symbol
_atom_site_label
_atom_site_symmetry_multiplicity

```
_atom_site_fract_x
_atom_site_fract_y
_atom_site_fract_z
_atom_site_occupancy
Mg Mg0 3 0.0 0.0 0.0 1.0
Ta Ta1 3 0.0 0.0 0.42 1.0
Pt Pt2 3 0.0 0.0 0.47 1.0
```

Z-matrix The z-matrix is a representation widely used as input for quantum mechanical simulations of small molecules (but not materials).[60] It leverages internal coordinates and is hence invariant with respect to translation or rotation. The internal coordinates used in a z-matrix are bond distances, angles, as well as dihedral angles. As all of these internal coordinates are defined with respect to neighboring atoms, the representation implicitly also encodes bonds. Here, we define the z-matrix based on the atoms within one unit cell. Z-Matrix is part of the representation group incorporating geometry inductive biases (spatial information) but they also have composition information (categorical information).

Supplementary Box 9: Z-Matrix

- Mg
  Ta 1 6.1
  Pt 2 0.5 1 0

Each of these representations, have different range of lengths. As the information content increase the representation length and the tokens required to model these system increases. Since there is a big variation in the representation length, we have modeled them with different context lengths, but fixed amongst representations. Supplementary Table 2 indicates the context length used for modeling different representations.

Supplementary Table 2: Representations and their corresponding context lengths. Fixed context lengths used for modeling each representation in MatText; context length is held constant within a representation across all experiments.

Representation	Context Length
SLICES	512
Composition	32
Crystal-text-LLM	512
Z-Matrix	512
CIF P_1	1024
CIF Symmetrized	1024
Atom Sequence	32
Atom Sequence++	32
Local-Env	512

3 Examples of synthetic data

The synthetic datasets used for the coordinate-category experiments described in the main manuscript are constructed from materials-inspired configurations with generic type labels replacing element symbols. Below we show the same data point in two different representations, along with its target value $E(\alpha)$ at three mixing ratios for that particular example.

Supplementary Box 10: Category & Category-Coordinate based representations

- `A B C C D D D D D D  6.14 6.14 6.14 47 47 47`

- ```
  6.1 6.1 6.1
  47 47 47
  A 0.00 0.00 0.00
  B 0.50 0.50 0.50
  C 0.76 0.76 0.76
  C 0.24 0.24 0.24
  D 0.73 0.49 0.05
  D 0.05 0.73 0.49
  D 0.51 0.95 0.27
  ...
  ```
  ```

The corresponding target values are $E(\alpha{=}0) = 0.06$, $E(\alpha{=}0.5) = 0.13$, and $E(\alpha{=}1) = 0.20$.

4 Attention analysis

The amount of attention received by different tokens can be interpreted as a measure of the relevance of different tokens contained in the representations (Supplementary Section 2).

The models here attend most to atomic symbols. This observation aligns with our primary findings, which show a strong correlation between leveraging compositional information (Figure 5).

Consistently, we also observe that numbers generally receive less attention. Overall, this supports the hypothesis that current models do not effectively utilize numerical information for learning complex geometric features.

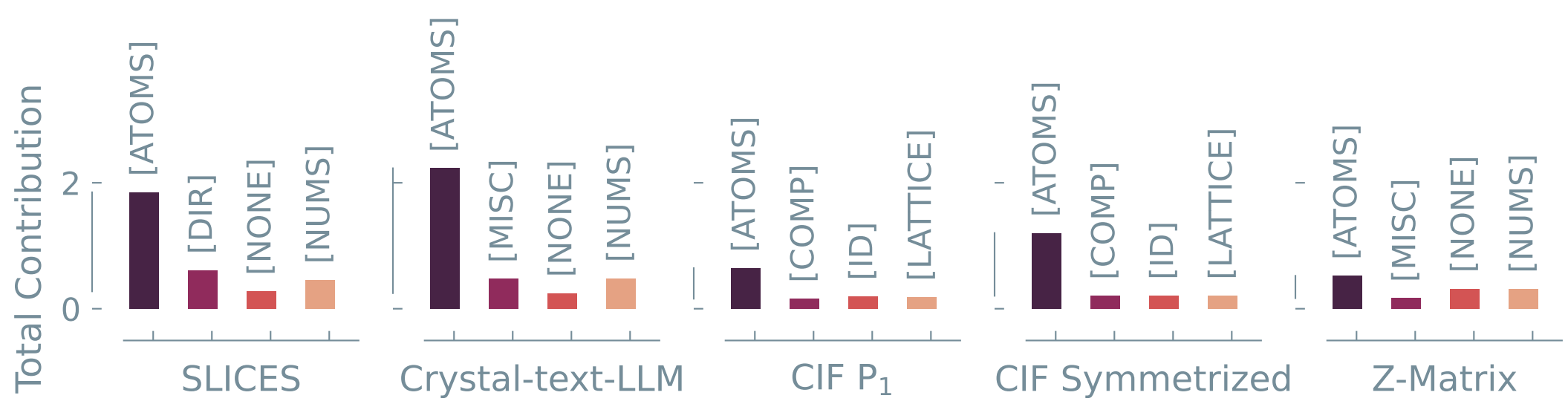

Supplementary Figure 1: Attention received by different types of tokens in different representations summed across all the heads and layers. Composition related (categorical information) tokens receives highest contributions and surprisingly numbers receive less attention leading to spatial information not being leveraged in prediction

Token attention contribution calculation To perform this analysis, we first compute the contribution per token.

The element-wise multiplication of the attention matrix $A^{(l,h)}$ and mask M_k gives the contribution of the attention scores for the token type k:

$$C_k^{(l,h)} = A^{(l,h)} \odot M_k \tag{1}$$

Here, $\odot$ denotes element-wise multiplication.

In this context, $A^{(l,h)}$ represents the attention matrix for layer l and head h, and M_k is the mask for token type k in tokenized material text representations. Tokens can be classified into different types for analytical purposes. For example, the SLICES representations can

have tokens of the type ATOMS, NUMS, and DIR. Specifically, all atoms are classified under the ATOMS token type, numbers are classified under NUM, and DIR represents tokens defining the direction of bonds.

The mask M_k is defined as:

$$M_k \in \{0, 1\}^{T \times T}, \tag{2}$$

where M_k is a binary matrix taking values 0 or 1.

The dimension of M_k matches that of the attention weight matrix $A^{(l,h)}$. Given that samples in the dataset may contain varying numbers of atoms, each sample can have different corresponding masks. To facilitate this analysis, the MatText tokenizers provide the functionality to generate a list of token types alongside the list of tokens. These token types are used dynamically to design masks for attention analysis.

Token Weight The percentage weight for token type k in layer l and head h is then calculated as:

$$W_k^{(l,h)} = \frac{\sum_{i,j}\left(C_k^{(l,h)}(i,j)\right)}{\sum_{i,j} M_k(i,j)}. \tag{3}$$

Here $W_k^{(l,h)}$ is the percentage attention recieved by a particular token type k in layer l and head h during prediction and $\sum_{i,j}$ denotes summing over all elements in the matrix.

Aggregation Across Folds We aggregate the attention weights for all the samples across multiple folds. This involves summing the weights for each token across all samples (N) and folds (f). The total contribution of token type k across all folds is given by:

$$T_k = \sum_{n=1}^{N} \sum_{f} \sum_{l,h} W_k^{(l,h,f,n)}. \tag{4}$$

Results In the attention heat maps, we observe certain heads specialized to learn features from compositions, which is not the case for numbers, where we observe rather a dispersed nature in heads (Supplementary Figure 1). Previously, with unsupervised language modeling of proteins, the formation of such heads was associated with parts of the architecture concentrating on learning certain features.[6] We observe that groups dedicated to learning numerical features do not emerge with pretraining.

5 Tokenizer

Low performance of LLMs has often been associated to tokenizers where numbers might not be processed correctly due to the tokenization method.[61,62] For instance, in many

default tokenization methods (for example, BPE, single-digit tokenization), numbers are represented with varying numbers of tokens, which might make it more difficult for models to use them effectively. To address this issue, we implemented the tokenizer proposed by Born & Manica[23], which preserves decimal order and also encodes the order of magnitude. We find that this change in tokenizer does not provide consistent improvements in modeling performance.

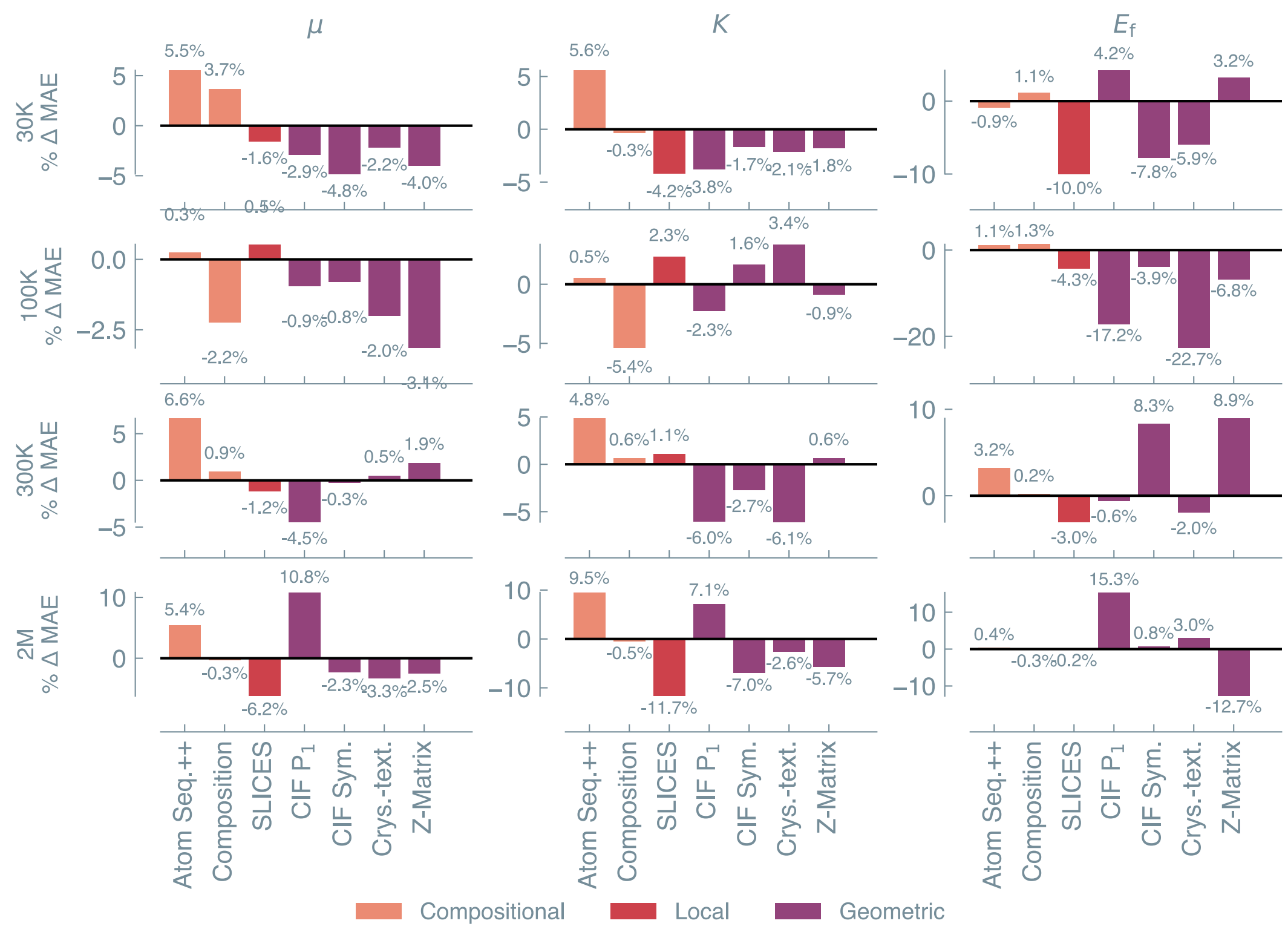

Supplementary Figure 2: Performance comparison between the RT tokenizer[23] and the regular tokenizer across different data scales and representation types. Each subplot shows the percentage improvement in Root Mean Square Error (RMSE) when using RT tokenizer compared to normal tokenizer, where positive values indicate RT tokenizer performs better. The plot is organized with four rows representing different training dataset sizes (30K, 100K, 300K, 2M) and three columns representing different material properties: shear modulus (μ), bulk modulus (κ), and formation energy of perovskites (E_f). Bars are colored by representation group: orange for compositional representations (Atom Sequence++, Composition), red for local representations (SLICES), and purple for geometric representations (Crystal-Text-LLM, CIF variants, Z-Matrix).

The RT tokenizer shows predominantly mixed effects on model performance with high variability across properties and representations. While there are minor improvements for some representations at large scales (particularly for geometric representations like Z-Matrix), the overall pattern reveals inconsistent and context-dependent performance changes. Formation Energy (E_f) exhibits particularly high variation, with some representations showing substantial improvements while others demonstrate substantial degradations. Bulk Modulus (κ) shows the most consistent behavior but still with mixed results, while Shear Modulus (μ) demonstrates largely neutral to modest effects. The amplification of variability at larger scales (300K, 2M) suggests that RT tokenizer's impact becomes more unpredictable rather than universally beneficial with increased training data.

6 Coordinate-Category Cliff

We compute CC-Cliff for different representations with different inductive biases for different material science property dataset. Again for the purpose of quantifying this metric, we can compute CC-Cliff as coordinate contribution ($L_{coord,\alpha}$) − category contribution ($L_{cat,\alpha}$). Positive and big CC-Cliff indicate that the model finds it substantially harder to learn from coordinate information than from category information. In Supplementary Figure 3 we have plotted CC-Cliff for material property prediction. Irrespective of the representations we find that for all datasets, models struggle leveraging coordinate information from the representations.

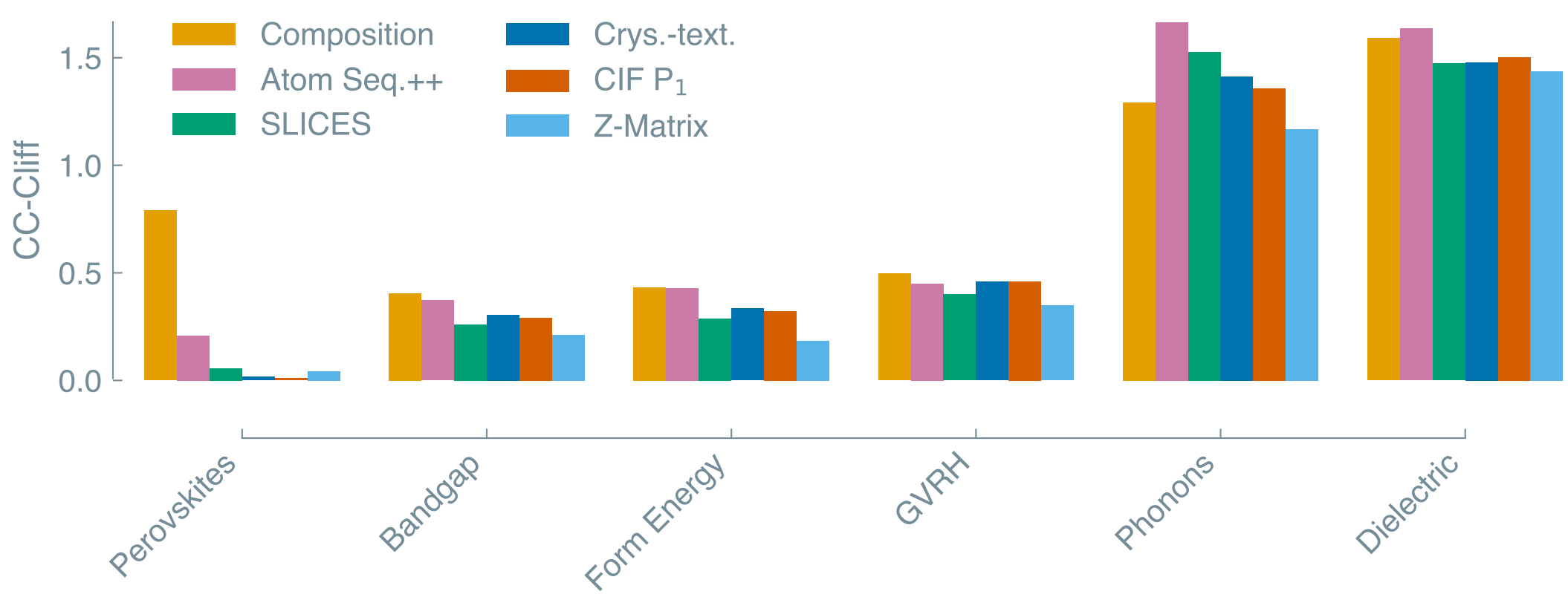

Supplementary Figure 3: CC-Cliff for different representations for different properties. Each property is a dataset with multiple crystal structures. All representations are created and modeled using MatText framework. Dataset with complex structures like dielectric materials have higher CC-Cliff values, while simpler structures like that of small perovskite structure dataset show lower CC-Cliff.

6.1 Interpretation of CC-Cliff Values

The CC-Cliff metric is defined as $\text{CC-Cliff} = L_{\text{coord},\alpha} - L_{\text{cat},\alpha}$, where both $L_{\text{coord},\alpha}$ and $L_{\text{cat},\alpha}$ measure how much the model's error deviates from the balanced ($\alpha = 0.5$) baseline in the coordinate-dominated and category-dominated regimes, respectively. Different values of the CC-Cliff correspond to qualitatively distinct learning behaviors, illustrated schematically in Supplementary Figure 4:

Positive CC-Cliff ($L_{\text{coord},\alpha} \gg L_{\text{cat},\alpha}$). The model struggles substantially more with coordinate-dominated tasks than with category-dominated ones. The underlying loss decreases monotonically as α increases from 0 to 1. The model performs poorly when the target depends heavily on positional information and well when it depends on categorical information. This is the dominant pattern we observe for LLMs across all experiments.

CC-Cliff ≈ 0: flat loss curve. Both $L_{\text{coord},\alpha} \approx 0$ and $L_{\text{cat},\alpha} \approx 0$. The model's error is approximately constant across the full range of α, indicating that the model's error is independent offwhether the ground truth depends mostly on coordinate or categorical information.

CC-Cliff ≈ 0: U- or V-shaped loss curve. This case is distinct from the flat curve above, despite yielding the same CC-Cliff value. Here, $L_{\text{coord},\alpha} > 0$ and $L_{\text{cat},\alpha} > 0$, but symmetrically, so their difference is near zero. The model performs best in the balanced regime ($\alpha = 0.5$) and degrades at both extremes, suggesting it benefits from having *both* types of information simultaneously, but struggles when either dominates exclusively. This case would indicate a model's inability to operate well when the signal is one-sided.

Negative CC-Cliff ($L_{\text{cat},\alpha} \gg L_{\text{coord},\alpha}$). The model struggles more with category-dominated tasks than coordinate-dominated ones. The loss curve increases monotonically with α, reflecting better performance when coordinates dominate. This pattern could arise in models with strong geometric inductive biases applied to tasks where categorical variation is high but geometric variation is constrained. We do not observe this regime for LLMs in our experiments. Our analysis with `COGN`[63] and `MODNet`[64] (see Section 2.1) shows that graph neural network (GNN)-based models can exhibit reduced CC-Cliff values in some datasets.

6.2 Comparison of CC-Cliff with `n-gram` models

In Supplementary Figure 5 we find that for all datasets we analyzed, the behavior of `n-gram` models closely follows that of the LLMs fine-tuned on different representations. More methodological details are given in Section 4.2.

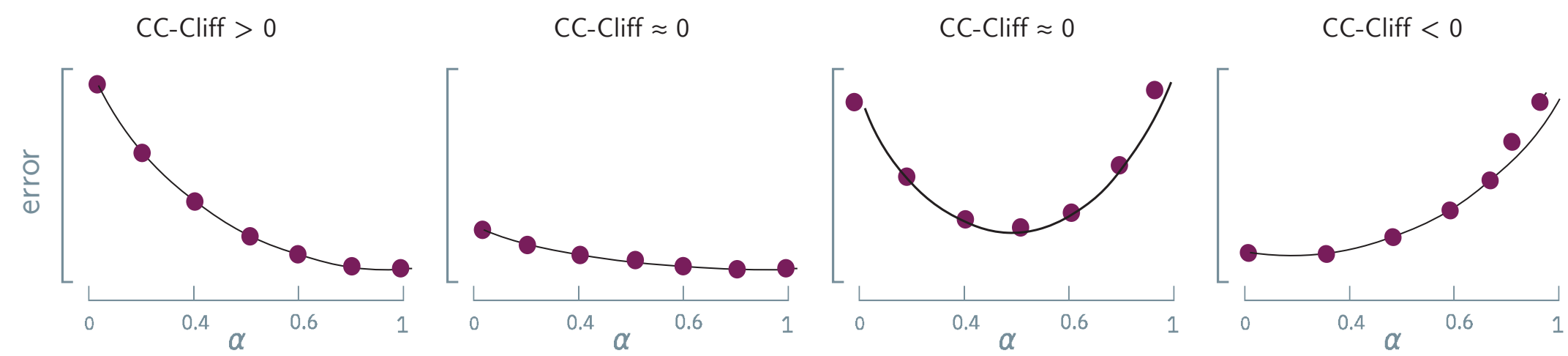

Supplementary Figure 4: Schematic illustration of qualitatively distinct CC-Cliff profiles. Each panel shows an idealized loss curve as a function of the mixing parameter α, which interpolates between coordinate-dominated ($\alpha = 0$) and category-dominated ($\alpha = 1$) targets. (*Left*) A monotonically decreasing loss curve yields a large positive CC-Cliff, indicating the model struggles with coordinate information — the dominant pattern observed for LLMs. (*Centre-left*) A flat curve yields CC-Cliff ≈ 0 with both contributions near zero, indicating the model is insensitive to the nature of the input signal. (*Centre-right*) A U-shaped curve also yields CC-Cliff ≈ 0, but here both contributions are positive and symmetric, indicating the model benefits from a balance of both signal types. (*Right*) A monotonically increasing loss curve yields a negative CC-Cliff, indicating that the models struggle more with categorical information than coordinate information

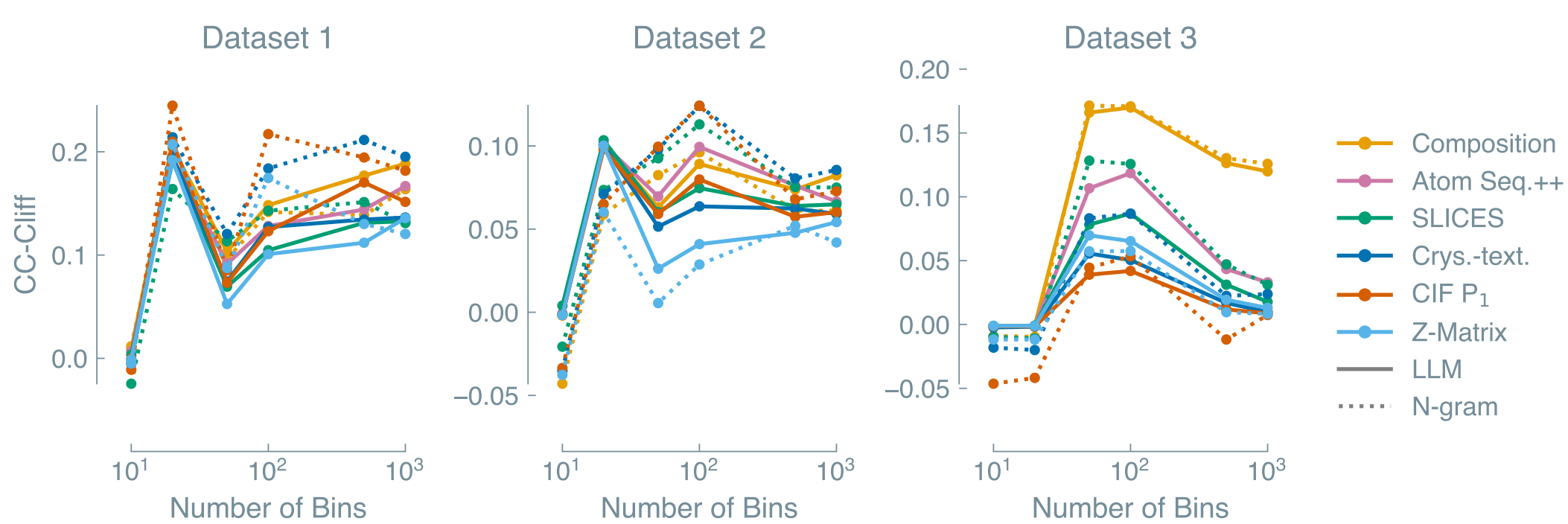

Supplementary Figure 5: CC-Cliff in hypothetical potential energy prediction as a function of binning the potential across different datasets and representations. The figure illustrates the CC-Cliff for models fine-tuned on different representations tasked with predicting hypothetical potential energy. The analysis is performed across three distinct datasets. The plot shows almost identical behavior of language models to that of `n-gram` models suggesting that LLMs inherit the properties of `n-gram` for tasks involving coordinate and category data irrespective of representation.

6.3 Relationship of CC-Cliff with diversity

From Supplementary Section 6 we see that CC-Cliff exhibits correlation with different datasets, however it is not clear why certain datasets show bigger CC-Cliff, that is, why in certain crystal structure datasets it seems difficult for LLM to learn the geometric relationship more. This could be because of the type of crystal structures, often some materials has complex coordination and hence can have complex geometric relationships. To understand the impact of the diversity of the structures in dataset, we benchmarked the diversity of the datasets by computing the coupling strength, geometric variance and diversity volume. The diversity volume in different datasets is quantified using convex hull volume calculated from the first two components of PCA projection of material embeddings. We apply the embeddings obtained using the `ChgNet`[65] checkpoint v0.3. The volume (or area in 2D) of the convex hull provides a measure of how "spread out" the structures are in the latent space. A larger volume indicates more structural diversity. Figure 6b shows convex hull for embedding space using `ChgNet` model.

Geometric variance how much the geometric properties vary among structures with the same composition. For structures with identical composition, let $\mathbf{G}_i = [g_{i,1}, g_{i,2}, \ldots, g_{i,7}]$ be the geometric fingerprint of structure i, where each $g_{i,j}$ represents a statistical measure of pairwise atomic distances (min, max, mean, median, std, Q25, Q75). The geometric variance is:

$$\text{geometric variance}_c = \frac{1}{7}\sum_{j=1}^{7} \text{Var}(g_{i,j}) \tag{5}$$

Lower geometric variance indicates that composition more strongly determines structure, while higher variance suggests greater structural diversity for the same composition. Coupling strength (Supplementary Equation 6) measures how strongly composition determines structure.

$$\text{coupling strength} = \frac{1}{1 + \text{mean\_geometric\_variance}} \tag{6}$$

We computed the diversity metrics and compared CC-Cliff for each of these metrics for different representations. We see from Figure 6a that CC-Cliff in general increases with diversity but not consistently.

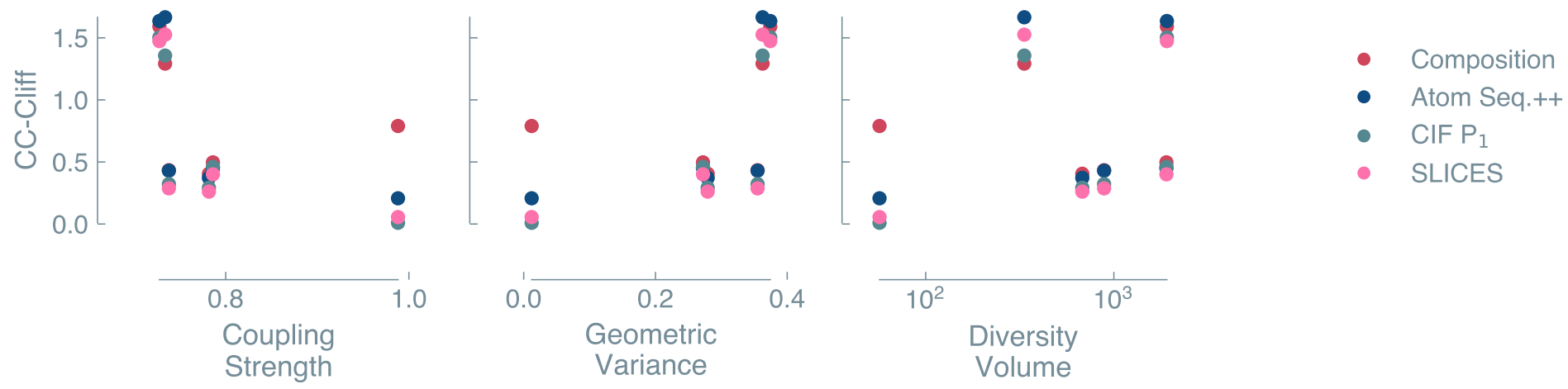

(a) Comparison between structural diversity and the CC-Cliff across different representations. Each point represents the CC-Cliff of the representations. Higher coupling strength indicates that the composition more strictly constrains possible structural arrangements. Datasets used in this analysis are, Perovskites, MP-Formation energy, MP-Bandgap, GVRH, phonons and shear modulus. Error bars show the standard deviation within each representation group.

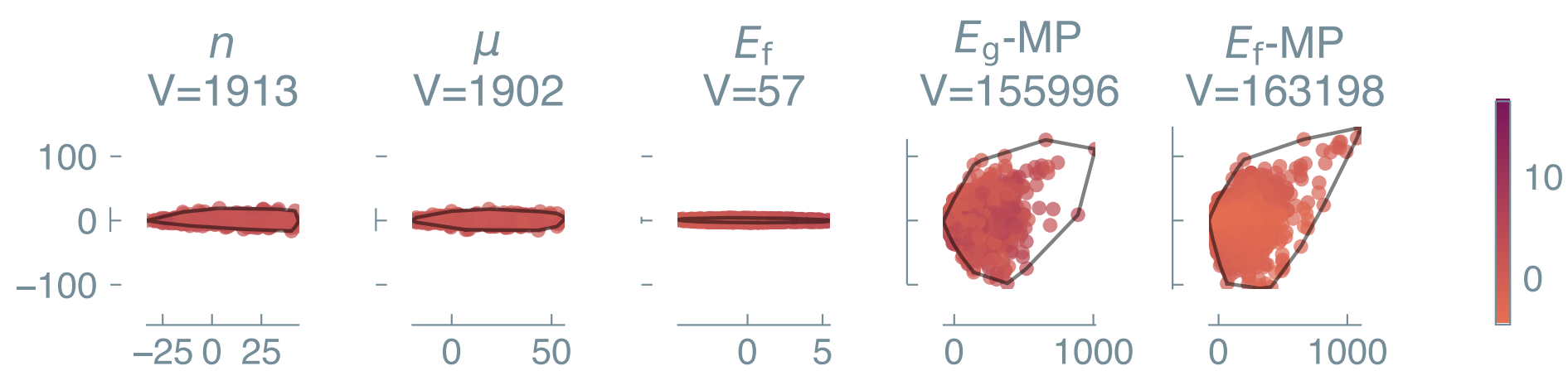

(b) Structural variations across different material property datasets. Diversity volume is quantified using the convex hull volume of the representation space. Higher volumes indicate greater structural diversity in the dataset, revealing how different representation approaches handle increasingly complex structural variations.

Supplementary Figure 6: CC-Cliff values versus structural diversity metrics across representations and datasets. Each panel shows CC-Cliff for a given representation against a diversity metric (geometric variance or convex-hull volume); rows correspond to material datasets and columns to diversity measures.

6.4 CC-Cliff across model architectures

To compare the CC-Cliff phenomenon beyond LLMs, we extend the analysis to include a graph neural network (`COGN`[63]) and a composition-featurized neural network (`MODNet`[64]). Both models are trained on the same synthetic dataset used for the language modelling experiments (section 2.1), across all seven mixing parameters $\alpha \in \{0, 0.2, 0.4, 0.5, 0.6, 0.8, 1.0\}$ and all using the same train/test splits.

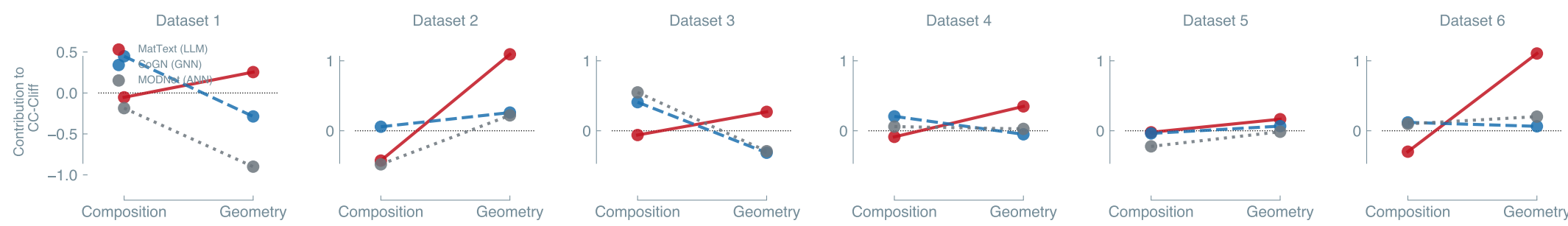

Supplementary Figure 7: Per-dataset coordinate and category contributions to the CC-Cliff for different architectures. Each subplot corresponds to one dataset. Dumbbells connect the category contribution (L_{category}, left endpoint) to the coordinate contribution ($L_{\text{coordinate}}$, right endpoint) for each model class.

The per-dataset CC-Cliff values for all three model classes are shown in Figure 2C (averaged) and Supplementary Figure 7 (per dataset). `COGN`, which explicitly encodes positions and inter distances in its graph representation, exhibits markedly reduced CC-Cliff values compared to LLMs across all datasets. `MODNet`, which relies on composition and structure-derived features with limited explicit geometric sensitivity, shows intermediate behaviour. These results indicate that the CC-Cliff is not an intrinsic property of the task, but reflects the degree to which a model's representation captures coordinate information.

We use the `COGN`[63] implementation from `kgcnn` v3.1.0 with the default architecture (`model_default`). Crystal structures are converted to graphs using a k-nearest neighbour preprocessor with $k = 24$ (`KNNAsymmetricUnitCell`). Models are trained for up to 800 epochs using the Adam optimizer with a polynomial learning rate decay schedule (initial rate 5×10^{-5}, final rate 10^{-5}), MAE loss, and gradient clipping (norm clip 0.5, value clip 0.1) to ensure stability. Early stopping with patience 50 is applied.

We use `MODNet`[64] v0.4.5 with composition and structure features computed via the `DeBreuck2020Featurizer` (`fast_oxid=True`). For each α-target, the top 100 features are selected by mutual information with the training targets. The model architecture comprises four fully connected layers with $[256, 128, 64, 32]$ neurons and ELU activations. Training uses Adam with learning rate 10^{-3}, batch size 64, and 200 epochs.

Supplementary Information References

1. Ruffolo, J. A. & Madani, A. Designing proteins with language models. *Nat. Biotechnol.* **42,** 200–202 (2024).
2. Rives, A. *et al.* Biological structure and function emerge from scaling unsupervised learning to 250 million protein sequences. *Proc. Natl. Acad. Sci.* **118** (2021).
3. Lin, Z. *et al.* Evolutionary-scale prediction of atomic-level protein structure with a language model. *Science* **379,** 1123–1130 (2023).
4. Elnaggar, A. *et al.* ProtTrans: Toward Understanding the Language of Life Through Self-Supervised Learning. *IEEE Trans. Pattern Anal. Mach. Intell.* **44,** 7112–7127 (2022).
5. Xu, M., Yuan, X., Miret, S. & Tang, J. Protst: Multi-modality learning of protein sequences and biomedical texts. *arXiv preprint arXiv:2301.12040* (2023).
6. Vig, J. *et al.* BERTology Meets Biology: Interpreting Attention in Protein Language Models. *arXiv preprint.* arXiv: `2006.15222 [cs.CL]` (2021).
7. Li, F.-Z., Amini, A. P., Yue, Y., Yang, K. K. & Lu, A. X. Feature Reuse and Scaling: Understanding Transfer Learning with Protein Language Models. *bioRxiv preprint* (2024).
8. Weininger, D. SMILES, a chemical language and information system. 1. Introduction to methodology and encoding rules. *J. Chem. Inf. Model* **28,** 31–36 (1988).
9. Krenn, M., Häse, F., Nigam, A., Friederich, P. & Aspuru-Guzik, A. Self-referencing embedded strings (SELFIES): A 100% robust molecular string representation. *Mach. Learn.: Sci. Technol.* **1,** 045024 (2020).
10. Cheng, A. H. *et al.* Group selfies: a robust fragment-based molecular string representation. *Digit. Discov.* **2,** 748–758 (2023).
11. Krenn, M. *et al.* SELFIES and the future of molecular string representations. *Patterns* **3,** 100588 (2022).
12. Bran, A. M. & Schwaller, P. Transformers and Large Language Models for Chemistry and Drug Discovery. *arXiv preprint arXiv:2310.06083.* arXiv: `2310.06083 [cs.LG]` (2023).
13. Cadeddu, A., Wylie, E. K., Jurczak, J., Wampler-Doty, M. & Grzybowski, B. A. Organic Chemistry as a Language and the Implications of Chemical Linguistics for Structural and Retrosynthetic Analyses. *Angew. Chem. Int. Ed.* **53,** 8108–8112 (2014).
14. Frey, N. C. *et al.* Neural scaling of deep chemical models. *Nat. Mach. Intell.* **5,** 1297–1305 (2023).
15. White, A. D. The future of chemistry is language. *Nat. Rev. Chem.* **7,** 457–458 (2023).
16. Noutahi, E., Gabellini, C., Craig, M., Lim, J. S. & Tossou, P. Gotta be SAFE: A New Framework for Molecular Design. *arXiv preprint arXiv:2310.10773* (2023).

17. Pesciullesi, G., Schwaller, P., Laino, T. & Reymond, J.-L. Transfer learning enables the molecular transformer to predict regio- and stereoselective reactions on carbohydrates. *Nat. Commun.* **11** (2020).

18. Schwaller, P. *et al.* Molecular Transformer: A Model for Uncertainty-Calibrated Chemical Reaction Prediction. *ACS Cent. Sci.* **5,** 1572–1583 (2019).

19. Chithrananda, S., Grand, G. & Ramsundar, B. ChemBERTa: Large-Scale Self-Supervised Pretraining for Molecular Property Prediction. *arXiv preprint arXiv:2010.09885.* arXiv: 2010.09885 [cs.LG] (2020).

20. Wang, S., Guo, Y., Wang, Y., Sun, H. & Huang, J. *SMILES-BERT: Large Scale Unsupervised Pre-Training for Molecular Property Prediction* in *Proceedings of the 10th ACM International Conference on Bioinformatics, Computational Biology and Health Informatics* (ACM, 2019).

21. Ahmad, W., Simon, E., Chithrananda, S., Grand, G. & Ramsundar, B. ChemBERTa-2: Towards Chemical Foundation Models. *arXiv preprint arXiv:2209.01712.* arXiv: 2209.01712 [cs.LG] (2022).

22. Balaji, S., Magar, R., Jadhav, Y. & Farimani, A. B. GPT-MolBERTa: GPT Molecular Features Language Model for molecular property prediction. *arXiv preprint arXiv:2310.03030.* arXiv: 2310.03030 [physics.chem-ph] (2023).

23. Born, J. & Manica, M. Regression Transformer enables concurrent sequence regression and generation for molecular language modelling. *Nat. Mach. Intell.* **5,** 432–444 (2023).

24. Bagal, V., Aggarwal, R., Vinod, P. K. & Priyakumar, U. D. MolGPT: Molecular Generation Using a Transformer-Decoder Model. *J. Chem. Inf. Model.* **62,** 2064–2076 (2021).

25. Grisoni, F. Chemical language models for de novo drug design: Challenges and opportunities. *Curr. Opin. Struct. Biol.* **79,** 102527 (2023).

26. Ghugare, R., Miret, S., Hugessen, A., Phielipp, M. & Berseth, G. Searching for High-Value Molecules Using Reinforcement Learning and Transformers. *arXiv preprint arXiv:2310.02902* (2023).

27. Hoffmann, R. How Chemistry and Physics Meet in the Solid State. *Angew. Chem., Int. Ed. Engl.* **26,** 846–878 (1987).

28. Ganose, A. M. & Jain, A. Robocrystallographer: automated crystal structure text descriptions and analysis. *MRS Commun.* **9,** 874–881 (2019).

29. Qu, J. *et al.* Leveraging language representation for materials exploration and discovery. *npj Comput. Mater.* **10** (2024).

30. Sayeed, H. M., Baird, S. G. & Sparks, T. D. Structure feature vectors derived from Robocrystallographer text descriptions of crystal structures using word embeddings. *ChemRxiv preprint* (2023).

31. Korolev, V. & Protsenko, P. Toward accurate interpretable predictions of materials properties within transformer language models. *arXiv preprint arXiv:2303.12188* (2023).

32. Bucior, B. J. *et al.* Identification Schemes for Metal–Organic Frameworks To Enable Rapid Search and Cheminformatics Analysis. *Cryst. Growth Des.* **19,** 6682–6697 (2019).

33. Cao, Z., Magar, R., Wang, Y. & Barati Farimani, A. MOFormer: Self-Supervised Transformer Model for Metal–Organic Framework Property Prediction. *J. Am. Chem. Soc.* **145,** 2958–2967 (2023).

34. Goodall, R. E., Parackal, A. S., Faber, F. A., Armiento, R. & Lee, A. A. Rapid discovery of stable materials by coordinate-free coarse graining. *Science advances* **8,** eabn4117 (2022).

35. De Breuck, P.-P., Piracha, H. A., Rignanese, G.-M. & Marques, M. A. L. A generative material transformer using Wyckoff representation. *npj Computational Materials* **12,** 60 (2026).

36. Musil, F. *et al.* Physics-Inspired Structural Representations for Molecules and Materials. *Chem. Rev.* **121,** 9759–9815 (2021).

37. Prodan, E. & Kohn, W. Nearsightedness of electronic matter. *Proc. Natl. Acad. Sci.* **102,** 11635–11638 (2005).

38. Sommer, J., Hetzel, L., Lüdke, D., Theis, F. & Günnemann, S. The power of motifs as inductive bias for learning molecular distributions. *arXiv preprint arXiv:2306.17246.* arXiv: `2306.17246 [cs.LG]` (2023).

39. Cheng, A. H. *et al.* Group SELFIES: a robust fragment-based molecular string representation. *Digit. Discov.* **2,** 748–758 (2023).

40. Langer, M. F., Goeßmann, A. & Rupp, M. Representations of molecules and materials for interpolation of quantum-mechanical simulations via machine learning. *npj Comput. Mater.* **8** (2022).

41. Batatia, I., Kovacs, D. P., Simm, G., Ortner, C. & Csányi, G. MACE: Higher order equivariant message passing neural networks for fast and accurate force fields. *Adv. Neur. In.* **35,** 11423–11436 (2022).

42. Satorras, V. G., Hoogeboom, E. & Welling, M. *E(n) equivariant graph neural networks* in *International conference on machine learning* (2021), 9323–9332.

43. Batzner, S. *et al.* E(3)-equivariant graph neural networks for data-efficient and accurate interatomic potentials. *Nat. Commun.* **13** (2022).

44. Thomas, N. *et al.* Tensor field networks: Rotation- and translation-equivariant neural networks for 3D point clouds. *arXiv preprint arXiv:1802.08219.* arXiv: `1802.08219 [cs.LG]` (2018).

45. Duval, A. *et al.* A hitchhiker's guide to geometric gnns for 3d atomic systems. *arXiv preprint arXiv:2312.07511* (2023).

46. Tian, S. I. P., Walsh, A., Ren, Z., Li, Q. & Buonassisi, T. What Information is Necessary and Sufficient to Predict Materials Properties using Machine Learning? *arXiv preprint arXiv:2206.04968.* arXiv: `2206.04968 [cond-mat.mtrl-sci]` (2022).

47. Jha, D. *et al.* ElemNet: Deep Learning the Chemistry of Materials From Only Elemental Composition. *Sci. Rep.* **8** (2018).

48. Wang, A. Y.-T., Kauwe, S. K., Murdock, R. J. & Sparks, T. D. Compositionally restricted attention-based network for materials property predictions. *npj Comput. Mater.* **7** (2021).

49. Hill, E. A. ON A SYSTEM OF INDEXING CHEMICAL LITERATURE; ADOPTED BY THE CLASSIFICATION DIVISION OF THE U. S. PATENT OFFICE.1. *J. Am. Chem. Soc.* **22,** 478–494 (1900).

50. Xiao, H. *et al.* An invertible, invariant crystal representation for inverse design of solid-state materials using generative deep learning. *Nature Communications* **14,** 7027 (2023).

51. Pauling, L. *The nature of the chemical bond* 3rd ed. (Cornell University Press, Ithaca, NY, 1960).

52. Waroquiers, D. *et al.* ChemEnv: a fast and robust coordination environment identification tool. *Acta Crystallogr. B Struct. Sci. Cryst. Eng. Mater.* **76,** 683–695 (2020).

53. Togo, A. & Tanaka, I. spglib: a software library for crystal symmetry search. *arXiv preprint arXiv:1808.01590* (2018).

54. O'Boyle, N. M. *et al.* Open Babel: An open chemical toolbox. *J. Cheminf.* **3** (2011).

55. Gruver, N. *et al. Fine-Tuned Language Models Generate Stable Inorganic Materials as Text* in *The Twelfth International Conference on Learning Representations, ICLR 2024, Vienna, Austria, May 7-11, 2024* (OpenReview.net, 2024).

56. Hall, S. R., Allen, F. H. & Brown, I. D. The crystallographic information file (CIF): a new standard archive file for crystallography. *Acta Cryst. A* **47,** 655–685 (1991).

57. Flam-Shepherd, D. & Aspuru-Guzik, A. Language models can generate molecules, materials, and protein binding sites directly in three dimensions as xyz, cif, and pdb files. *arXiv preprint arXiv:2305.05708* (2023).

58. Ding, Q., Miret, S. & Liu, B. *MatExpert: Decomposing Materials Discovery By Mimicking Human Experts* in *The Thirteenth International Conference on Learning Representations* (2025).

59. Antunes, L. M., Butler, K. T. & Grau-Crespo, R. Crystal structure generation with autoregressive large language modeling. *arXiv preprint arXiv:2307.04340* (2023).

60. Cramer, C. J. *Essentials of computational chemistry: theories and models* (John Wiley & Sons, 2013).

61. Singh, A. K. & Strouse, D. Tokenization counts: the impact of tokenization on arithmetic in frontier LLMs. *arXiv preprint:2402.14903.* arXiv: `2402.14903 [cs.CL]` (2024).

62. Zausinger, J. *et al.* Regress, Don't Guess - A Regression-like Loss on Number Tokens for Language Models. *arXiv preprint arXiv: 2411.02083* (2024).

63. Ruff, R., Reiser, P., Stühmer, J. & Friederich, P. Connectivity optimized nested line graph networks for crystal structures. *Digital Discovery* **3,** 594–601 (2024).

64. De Breuck, P.-P., Evans, M. L. & Rignanese, G.-M. Robust model benchmarking and bias-imbalance in data-driven materials science: a case study on MODNet. *Journal of Physics: Condensed Matter* **33,** 404002 (2021).

65. Deng, B. *et al.* CHGNet as a pretrained universal neural network potential for charge-informed atomistic modelling. *Nature Machine Intelligence* **5,** 1031–1041 (2023).
