# Supplementary material for: Less can be more for predicting properties with large language models
Source: Nat Comput Sci. 2026 Oct 12;6(10):1124–32. doi: 10.1038/s43588-026-01045-2 (PMC13686937; doi:10.1038/s43588-026-01045-2)
Supplement: Supplementary file 2 [file 43588_2026_1045_MOESM2_ESM.pdf]

nature portfolio

Peer Review File

Less can be more for predicting properties with large language models

Corresponding Author: Dr Kevin Maik Jablonka

Version 0:

Reviewer comments:

Reviewer #1

(Remarks to the Author)
Co-reviewed with Reviewer #4

(Remarks on code availability)

Reviewer #2

(Remarks to the Author)
Co-reviewed with Reviewer #3

This paper addresses the important and timely topic of the use of LLMs in scientific machine learning, focusing on the impact of coordinate data on the prediction performance. The authors present a comprehensive analysis by designing an original approach to assess the sensitivity of an LLM to coordinate-categorical data. Their publicly available and extensive benchmarking framework is a notable step in a more responsible use of LLMs in materials science and further demonstrates that LLMs are ill-suited for tasks primarily governed by structural properties. The manuscript is well-structured, with clear methodology and thorough discussion. It provides meaningful insights into the use of LLMs in materials science. While the study is robust, revisions are needed to further strengthen and clarify the paper. Once our comments (see below) are addressed, we will most probably be able to recommend this manuscript for publication in Nature Computational Science.
- Either we got it completely wrong or the ticks on the x-axis of Figure 2B are inverted, especially given that the caption explicitly states "Lcoord > Lcat." Please verify and correct this if necessary. On the contrary, please clarify.
- It would be helpful for the reader if the authors could schematize and provide an interpretation of the different cases for the CC-cliff, such as CC-cliff = 0 (e.g., distinguishing a straight horizontal line from a V- or U-shape), as well as negative values.
- Regarding the equation E=Ecoord+Ecat for different values of α, it seems important to ensure that E does not vary too much with respect to α. Without this, if Ecoord and Ecat values are on very different orders of magnitude, increasing the weight of Ecoord in E could trivially lead to a higher E even if Ecat is reduced, potentially resulting in a larger error compared to a balanced 50-50 case. A normalization step might be necessary to make the reasoning robust, unless this has already been implemented or taken into account in some way (in which case a few words should be added in the text).
- The methodology for computing the CC-cliff should ideally also be applied using one GNN and one other intermediate model (typically MODNet) to further investigate the shape of the cliff, particularly in light of Figure 7 (in order to have the CC-cliff for at least one blue, one red, and one grey circles). This would help differentiate the advantages and limitations of GNNs, LLMs, ANNs, and other models more clearly.
- In Section 2.2, our understanding of Figure 3 is that, for datasets 1 and 2, including coordinate data does not significantly impact the results, as the two solid lines are very close. In contrast, dataset 3 exhibits the behavior that we expected, where the importance of coordinate data becomes more pronounced as the number of bins increases, as evidenced by the high CC-cliff for category-only data. The authors do not discuss these differences (suggesting a potential explanation). Additional description and interpretation of these observations would definitely strengthen this section. Additionally, it should be clarified that the inputs of the LLMs are not affected by the binning process.
- The meaning of the dashed lines in Figure 6 is not specified in the caption. Please clarify this.
- Though they are material-specific, it might be appropriate to mention approaches in which the coordinates are translated into text using Wyckoff positions (as in Wren [Goodall et al., Sci. Adv. 8, eabn4117 (2022)] or Matra-Genoa [https://arxiv.org/abs/2501.16051]). Indeed, such approaches might alleviate some of the challenges associated with handling coordinates by categorizing them. Has there been any investigation into such material-text translations in the context of MatText or the present paper?
Minor points:

- For clarity, it would be beneficial to maintain consistency in terminology throughout the manuscript. For example, avoid using "train/trained/training" when referring to "fine-tune/fine-tuned/fine-tuning," as this could introduce ambiguity regarding different training stages.
- The term "labels" is occasionally used to refer to the "scalar target values" which could be confusing since the categorical data is also sometimes referred to as categorical labels. Clarifying or standardizing this terminology would improve readability.
- There are minor formatting issues to address: a missing parenthesis in the caption of Figure 2 (Lcat,α), and a missing final period at the end of the caption of Figure 7.
- On page 10, the statement "there is no effect of either scaling dataset size or model parameter count" seems inconsistent with Figure 6, which shows improvements in RMSE of up to 10% and even 50%. This sentence should be clarified, possibly by quantifying the observed effects.

Victor Trinquet and Gian-Marco Rignanese

(Remarks on code availability)

Reviewer #3

(Remarks to the Author)
Co-reviewed with Reviewer #2

This paper addresses the important and timely topic of the use of LLMs in scientific machine learning, focusing on the impact of coordinate data on the prediction performance. The authors present a comprehensive analysis by designing an original approach to assess the sensitivity of an LLM to coordinate-categorical data. Their publicly available and extensive benchmarking framework is a notable step in a more responsible use of LLMs in materials science and further demonstrates that LLMs are ill-suited for tasks primarily governed by structural properties. The manuscript is well-structured, with clear methodology and thorough discussion. It provides meaningful insights into the use of LLMs in materials science. While the study is robust, revisions are needed to further strengthen and clarify the paper. Once our comments (see below) are addressed, we will most probably be able to recommend this manuscript for publication in Nature Computational Science.

- Either we got it completely wrong or the ticks on the x-axis of Figure 2B are inverted, especially given that the caption explicitly states "Lcoord > Lcat." Please verify and correct this if necessary. On the contrary, please clarify.

- It would be helpful for the reader if the authors could schematize and provide an interpretation of the different cases for the CC-cliff, such as CC-cliff = 0 (e.g., distinguishing a straight horizontal line from a V- or U-shape), as well as negative values.

- Regarding the equation E=Ecoord+Ecat for different values of α, it seems important to ensure that E does not vary too much with respect to α. Without this, if Ecoord and Ecat values are on very different orders of magnitude, increasing the weight of Ecoord in E could trivially lead to a higher E even if Ecat is reduced, potentially resulting in a larger error compared to a balanced 50-50 case. A normalization step might be necessary to make the reasoning robust, unless this has already been implemented or taken into account in some way (in which case a few words should be added in the text).

- The methodology for computing the CC-cliff should ideally also be applied using one GNN and one other intermediate model (typically MODNet) to further investigate the shape of the cliff, particularly in light of Figure 7 (in order to have the CC-cliff for at least one blue, one red, and one grey circles). This would help differentiate the advantages and limitations of GNNs, LLMs, ANNs, and other models more clearly.

- In Section 2.2, our understanding of Figure 3 is that, for datasets 1 and 2, including coordinate data does not significantly impact the results, as the two solid lines are very close. In contrast, dataset 3 exhibits the behavior that we expected, where the importance of coordinate data becomes more pronounced as the number of bins increases, as evidenced by the high CC-cliff for category-only data. The authors do not discuss these differences (suggesting a potential explanation). Additional description and interpretation of these observations would definitely strengthen this section. Additionally, it should be clarified that the inputs of the LLMs are not affected by the binning process.

- The meaning of the dashed lines in Figure 6 is not specified in the caption. Please clarify this.

- Though they are material-specific, it might be appropriate to mention approaches in which the coordinates are translated into text using Wyckoff positions (as in Wren [Goodall et al., Sci. Adv. 8, eabn4117 (2022)] or Matra-Genoa [https://arxiv.org/abs/2501.16051]). Indeed, such approaches might alleviate some of the challenges associated with handling coordinates by categorizing them. Has there been any investigation into such material-text translations in the context of MatText or the present paper?

Minor points:

- For clarity, it would be beneficial to maintain consistency in terminology throughout the manuscript. For example, avoid using "train/trained/training" when referring to "fine-tune/fine-tuned/fine-tuning," as this could introduce ambiguity regarding different training stages.

- The term "labels" is occasionally used to refer to the "scalar target values" which could be confusing since the categorical data is also sometimes referred to as categorical labels. Clarifying or standardizing this terminology would improve readability.

- There are minor formatting issues to address: a missing parenthesis in the caption of Figure 2 (Lcat,α), and a missing final period at the end of the caption of Figure 7.

- On page 10, the statement "there is no effect of either scaling dataset size or model parameter count" seems inconsistent with Figure 6, which shows improvements in RMSE of up to 10% and even 50%. This sentence should be clarified, possibly by quantifying the observed effects.

Victor Trinquet and Gian-Marco Rignanese

(Remarks on code availability)
I visited the githup repository of MatText, which has a nice but short README with information on the installation and the basic capabilities. More importantly, the documentation on the related webpage provides a great deal of information and example scripts in a clean and efficient format. I have not run the code, but I expect it to be a relatively smooth experience thanks to these resources.

Reviewer #4

(Remarks to the Author)
Co-reviewed with Reviewer #1

This work studies the limitations and strengths of using text/string-based representations with LLMs for materials property prediction. Through extensive and carefully designed experiments, the authors demonstrate that, regardless of model size, dataset size, or text representation strategy, LLMs fail to capture coordinate-level information while excelling at learning categorical patterns. Consequently, this study suggests using GNN–based approaches for tasks dominated by structural effects, and LLMs for tasks where categorical or compositional information is sufficient. Furthermore, the authors introduce MatText, a Python package that takes a CIF file as input and outputs multiple text representations of materials suitable for training LLMs or other text-based methods. MatText can also serve as a framework for end-to-end language modeling of materials, including data processing, model training, and results analysis. The paper is well written, structured, and easy to follow.

Overall, this is a solid piece of work. It addresses critical challenges in LLM- and string-based materials property prediction. The experiments are thorough, and the results are informative. I particularly appreciate the effort the authors have put into the experimental design and results analysis. It will certainly be of interest to the computational materials science community.

I have few questions for the authors:
1. In section 4.3, authors wrote that "There has been no tool to systematically derive text representations for materials.", I found this to be a strong/unfair claim given other efforts that had been made prior to this study, such as Robocrystallographer and MOFid tools. Please clarify.

2. What does the synthetic data you made in Section 2.1 look like? It would be useful to include one or two examples in the manuscript.

3. In section 4.4, authors mentioned that they have created pretraining datasets with varying datasize? How were these datasets created? I found little to no details on how they were created. Including these details would be very useful for reproducibility purposes.

4. I believe that MatText also supports Robocrystallographer crystal text descriptions, why were the results with this input representation not compared with other text representations, for example in Figure 5 and 6?

5. In Figure 7, which model and input representation does MatText correspond to? Please clarify.

6. In section 2.3, the authors mentioned that “Remarkably, we find that for all representations and prediction targets we investigated, there is no effect of either scaling dataset size or model parameter count (we provide more extensive details in section 5.1)”. I believe this contradicts with Figure 6. Where there is a clear trend on Mu and K properties which shows that as you increase the datasize the performance improves. And for Ef, a significant performance improvement is also observed with model scaling. Please clarify as I did not find any details in section 5.1 as mentioned in the paper.

Minor:
1. Citations 4 and 23 seem to be duplicates, also there is an updated peer-reviewed version of this work (https://www.nature.com/articles/s41524-025-01536-2), please check and update other citations accordingly.

2. "...in our calculations we maps chemical..." in section 4.1.1, is this a possible typo?

3. "...(e.g., CIF files) similar to that used for the LLMs)..." in section 4.2.2, possible extra parenthesis?

(Remarks on code availability)
1. When trying out MatText, I got the following package error despite following the proposed installation guidelines and running the "pip install e." with no error. I could not find it in packages supported by PyPI or conda. Including how to install this package would be very useful.

```
ModuleNotFoundError Traceback (most recent call last)
Cell In[1], line 1
----> 1 from mattext.representations import TextRep
2 from pymatgen.core.structure import Structure

File ~/mattext/src/mattext/representations/__init__.py:1
----> 1 from xtal2txt.core import TextRep
3 __all__ = ["TextRep"]

File ~/anaconda3/envs/mattext_env/lib/python3.9/site-packages/xtal2txt/core.py:7
4 from pathlib import Path
5 from typing import List, Union, Tuple, Optional
----> 7 from invcryrep.invcryrep import InvCryRep
8 from pymatgen.core import Structure
9 from pymatgen.core.structure import Molecule

ModuleNotFoundError: No module named 'invcryrep'
```

2. Also while you mentioned in "https://github.com/lamalab-org/MatText/blob/main/pyproject.toml" that MatText supports Python 3.12, I could not install it successfully until I downgraded the Python version to Python 3.9. Please double check this and confirm if it can be truly installed with Python 3.12 with no errors. The error log is added below.

```
Obtaining file:///content/mattext
Installing build dependencies ... done
Checking if build backend supports build_editable ... done
Getting requirements to build editable ... done
Preparing editable metadata (pyproject.toml) ... done
Collecting matbench@ git+https://github.com/materialsproject/matbench.git (from mattext==0.0.1)
Cloning https://github.com/materialsproject/matbench.git to /tmp/pip-install-
mkai5t8k/matbench_ca2ebde51c0642c5b5d2489c8b8354f2
Running command git clone --filter=blob:none --quiet https://github.com/materialsproject/matbench.git /tmp/pip-install-
mkai5t8k/matbench_ca2ebde51c0642c5b5d2489c8b8354f2
Resolved https://github.com/materialsproject/matbench.git to commit 936176db18ca4cd7b38cbd957c017a5bac770c6b
Installing build dependencies ... done
Getting requirements to build wheel ... done
Preparing metadata (pyproject.toml) ... done
Collecting xtal2txt (from mattext==0.0.1)
Downloading xtal2txt-0.1.0-py3-none-any.whl.metadata (7.5 kB)
Requirement already satisfied: transformers in /usr/local/lib/python3.12/dist-packages (from mattext==0.0.1) (4.57.3)
Requirement already satisfied: tokenizers in /usr/local/lib/python3.12/dist-packages (from mattext==0.0.1) (0.22.1)
Requirement already satisfied: datasets in /usr/local/lib/python3.12/dist-packages (from mattext==0.0.1) (4.0.0)
Collecting hydra-core (from mattext==0.0.1)
Downloading hydra_core-1.3.2-py3-none-any.whl.metadata (5.5 kB)
Requirement already satisfied: torch in /usr/local/lib/python3.12/dist-packages (from mattext==0.0.1) (2.9.0+cpu)
Requirement already satisfied: accelerate in /usr/local/lib/python3.12/dist-packages (from mattext==0.0.1) (1.12.0)
Collecting hydra-submitit-launcher (from mattext==0.0.1)
Downloading hydra_submitit_launcher-1.2.0-py3-none-any.whl.metadata (1.1 kB)
Requirement already satisfied: python-dotenv in /usr/local/lib/python3.12/dist-packages (from mattext==0.0.1) (1.2.1)
Collecting pystow (from mattext==0.0.1)
Downloading pystow-0.7.13-py3-none-any.whl.metadata (17 kB)
Requirement already satisfied: wandb in /usr/local/lib/python3.12/dist-packages (from mattext==0.0.1) (0.23.1)
Collecting fire (from mattext==0.0.1)
Downloading fire-0.7.1-py3-none-any.whl.metadata (5.8 kB)
Requirement already satisfied: peft in /usr/local/lib/python3.12/dist-packages (from mattext==0.0.1) (0.18.0)
Collecting trl (from mattext==0.0.1)
Downloading trl-0.26.2-py3-none-any.whl.metadata (11 kB)
Collecting bitsandbytes (from mattext==0.0.1)
Downloading bitsandbytes-0.49.0-py3-none-manylinux_2_24_x86_64.whl.metadata (10 kB)
Collecting loguru (from mattext==0.0.1)
```

Downloading loguru-0.7.3-py3-none-any.whl.metadata (22 kB)
Requirement already satisfied: numpy>=1.17 in /usr/local/lib/python3.12/dist-packages (from accelerate->mattext==0.0.1) (2.0.2)
Requirement already satisfied: packaging>=20.0 in /usr/local/lib/python3.12/dist-packages (from accelerate->mattext==0.0.1) (25.0)
Requirement already satisfied: psutil in /usr/local/lib/python3.12/dist-packages (from accelerate->mattext==0.0.1) (5.9.5)
Requirement already satisfied: pyyaml in /usr/local/lib/python3.12/dist-packages (from accelerate->mattext==0.0.1) (6.0.3)
Requirement already satisfied: huggingface_hub>=0.21.0 in /usr/local/lib/python3.12/dist-packages (from accelerate->mattext==0.0.1) (0.36.0)
Requirement already satisfied: safetensors>=0.4.3 in /usr/local/lib/python3.12/dist-packages (from accelerate->mattext==0.0.1) (0.7.0)
Requirement already satisfied: filelock in /usr/local/lib/python3.12/dist-packages (from torch->mattext==0.0.1) (3.20.0)
Requirement already satisfied: typing-extensions>=4.10.0 in /usr/local/lib/python3.12/dist-packages (from torch->mattext==0.0.1) (4.15.0)
Requirement already satisfied: setuptools in /usr/local/lib/python3.12/dist-packages (from torch->mattext==0.0.1) (75.2.0)
Requirement already satisfied: sympy>=1.13.3 in /usr/local/lib/python3.12/dist-packages (from torch->mattext==0.0.1) (1.14.0)
Requirement already satisfied: networkx>=2.5.1 in /usr/local/lib/python3.12/dist-packages (from torch->mattext==0.0.1) (3.6.1)
Requirement already satisfied: jinja2 in /usr/local/lib/python3.12/dist-packages (from torch->mattext==0.0.1) (3.1.6)
Requirement already satisfied: fsspec>=0.8.5 in /usr/local/lib/python3.12/dist-packages (from torch->mattext==0.0.1) (2025.3.0)
Requirement already satisfied: pyarrow>=15.0.0 in /usr/local/lib/python3.12/dist-packages (from datasets->mattext==0.0.1) (18.1.0)
Requirement already satisfied: dill<0.3.9,>=0.3.0 in /usr/local/lib/python3.12/dist-packages (from datasets->mattext==0.0.1) (0.3.8)
Requirement already satisfied: pandas in /usr/local/lib/python3.12/dist-packages (from datasets->mattext==0.0.1) (2.2.2)
Requirement already satisfied: requests>=2.32.2 in /usr/local/lib/python3.12/dist-packages (from datasets->mattext==0.0.1) (2.32.4)
Requirement already satisfied: tqdm>=4.66.3 in /usr/local/lib/python3.12/dist-packages (from datasets->mattext==0.0.1) (4.67.1)
Requirement already satisfied: xxhash in /usr/local/lib/python3.12/dist-packages (from datasets->mattext==0.0.1) (3.6.0)
Requirement already satisfied: multiprocess<0.70.17 in /usr/local/lib/python3.12/dist-packages (from datasets->mattext==0.0.1) (0.70.16)
Requirement already satisfied: termcolor in /usr/local/lib/python3.12/dist-packages (from fire->mattext==0.0.1) (3.2.0)
Requirement already satisfied: omegaconf<2.4,>=2.2 in /usr/local/lib/python3.12/dist-packages (from hydra-core->mattext==0.0.1) (2.3.0)
Requirement already satisfied: antlr4-python3-runtime==4.9.* in /usr/local/lib/python3.12/dist-packages (from hydra-core->mattext==0.0.1) (4.9.3)
Collecting hydra-core (from mattext==0.0.1)
Downloading hydra_core-1.4.0.dev1-py3-none-any.whl.metadata (5.4 kB)
Collecting submitit>=1.3.3 (from hydra-submitit-launcher->mattext==0.0.1)
Downloading submitit-1.5.4-py3-none-any.whl.metadata (7.4 kB)
Collecting matminer>=0.7.4 (from matbench@ git+https://github.com/materialsproject/matbench.git->mattext==0.0.1)
Downloading matminer-0.9.3-py3-none-any.whl.metadata (4.9 kB)
Requirement already satisfied: scipy>=1.9.0 in /usr/local/lib/python3.12/dist-packages (from matbench@ git+https://github.com/materialsproject/matbench.git->mattext==0.0.1) (1.16.3)
Collecting monty>=2022.4.26 (from matbench@ git+https://github.com/materialsproject/matbench.git->mattext==0.0.1)
Downloading monty-2025.3.3-py3-none-any.whl.metadata (3.6 kB)
Requirement already satisfied: scikit-learn>=1.0.1 in /usr/local/lib/python3.12/dist-packages (from matbench@ git+https://github.com/materialsproject/matbench.git->mattext==0.0.1) (1.6.1)
Requirement already satisfied: click in /usr/local/lib/python3.12/dist-packages (from pystow->mattext==0.0.1) (8.3.1)
Requirement already satisfied: regex!=2019.12.17 in /usr/local/lib/python3.12/dist-packages (from transformers->mattext==0.0.1) (2025.11.3)
Requirement already satisfied: gitpython!=3.1.29,>=1.0.0 in /usr/local/lib/python3.12/dist-packages (from wandb->mattext==0.0.1) (3.1.45)
Requirement already satisfied: platformdirs in /usr/local/lib/python3.12/dist-packages (from wandb->mattext==0.0.1) (4.5.1)
Requirement already satisfied: protobuf!=4.21.0,!=5.28.0,<7,>=3.19.0 in /usr/local/lib/python3.12/dist-packages (from wandb->mattext==0.0.1) (5.29.5)
Requirement already satisfied: pydantic<3 in /usr/local/lib/python3.12/dist-packages (from wandb->mattext==0.0.1) (2.12.3)
Requirement already satisfied: sentry-sdk>=2.0.0 in /usr/local/lib/python3.12/dist-packages (from wandb->mattext==0.0.1) (2.47.0)
Collecting slices (from xtal2txt->mattext==0.0.1)
Downloading slices-2.0.12-py3-none-any.whl.metadata (252 bytes)
Collecting robocrys (from xtal2txt->mattext==0.0.1)
Downloading robocrys-0.2.12-py3-none-any.whl.metadata (3.0 kB)
Collecting keras<3 (from xtal2txt->mattext==0.0.1)
Downloading keras-2.15.0-py3-none-any.whl.metadata (2.4 kB)
Requirement already satisfied: aiohttp!=4.0.0a0,!=4.0.0a1 in /usr/local/lib/python3.12/dist-packages (from fsspec[http]

```
<=2025.3.0,>=2023.1.0->datasets->mattext==0.0.1) (3.13.2)
Requirement already satisfied: gitdb<5,>=4.0.1 in /usr/local/lib/python3.12/dist-packages (from gitpython!=3.1.29,>=1.0.0-
>wandb->mattext==0.0.1) (4.0.12)
Requirement already satisfied: hf-xet<2.0.0,>=1.1.3 in /usr/local/lib/python3.12/dist-packages (from
huggingface_hub>=0.21.0->accelerate->mattext==0.0.1) (1.2.0)
Collecting pymongo~=4.5 (from matminer>=0.7.4->matbench@ git+https://github.com/materialsproject/matbench.git-
>mattext==0.0.1)
Downloading pymongo-4.15.5-cp312-cp312-
manylinux2014_x86_64.manylinux_2_17_x86_64.manylinux_2_28_x86_64.whl.metadata (22 kB)
Collecting pymatgen>=2023 (from matminer>=0.7.4->matbench@ git+https://github.com/materialsproject/matbench.git-
>mattext==0.0.1)
Downloading pymatgen-2025.10.7-cp312-cp312-
manylinux2014_x86_64.manylinux_2_17_x86_64.manylinux_2_28_x86_64.whl.metadata (13 kB)
Collecting ruamel.yaml (from monty>=2022.4.26->matbench@ git+https://github.com/materialsproject/matbench.git-
>mattext==0.0.1)
Downloading ruamel_yaml-0.18.17-py3-none-any.whl.metadata (27 kB)
Requirement already satisfied: python-dateutil>=2.8.2 in /usr/local/lib/python3.12/dist-packages (from pandas->datasets-
>mattext==0.0.1) (2.9.0.post0)
Requirement already satisfied: pytz>=2020.1 in /usr/local/lib/python3.12/dist-packages (from pandas->datasets-
>mattext==0.0.1) (2025.2)
Requirement already satisfied: tzdata>=2022.7 in /usr/local/lib/python3.12/dist-packages (from pandas->datasets-
>mattext==0.0.1) (2025.3)
Requirement already satisfied: annotated-types>=0.6.0 in /usr/local/lib/python3.12/dist-packages (from pydantic<3->wandb-
>mattext==0.0.1) (0.7.0)
Requirement already satisfied: pydantic-core==2.41.4 in /usr/local/lib/python3.12/dist-packages (from pydantic<3->wandb-
>mattext==0.0.1) (2.41.4)
Requirement already satisfied: typing-inspection>=0.4.2 in /usr/local/lib/python3.12/dist-packages (from pydantic<3->wandb-
>mattext==0.0.1) (0.4.2)
Requirement already satisfied: charset_normalizer<4,>=2 in /usr/local/lib/python3.12/dist-packages (from requests>=2.32.2-
>datasets->mattext==0.0.1) (3.4.4)
Requirement already satisfied: idna<4,>=2.5 in /usr/local/lib/python3.12/dist-packages (from requests>=2.32.2->datasets-
>mattext==0.0.1) (3.11)
Requirement already satisfied: urllib3<3,>=1.21.1 in /usr/local/lib/python3.12/dist-packages (from requests>=2.32.2-
>datasets->mattext==0.0.1) (2.5.0)
Requirement already satisfied: certifi>=2017.4.17 in /usr/local/lib/python3.12/dist-packages (from requests>=2.32.2-
>datasets->mattext==0.0.1) (2025.11.12)
Requirement already satisfied: joblib>=1.2.0 in /usr/local/lib/python3.12/dist-packages (from scikit-learn>=1.0.1-
>matbench@ git+https://github.com/materialsproject/matbench.git->mattext==0.0.1) (1.5.3)
Requirement already satisfied: threadpoolctl>=3.1.0 in /usr/local/lib/python3.12/dist-packages (from scikit-learn>=1.0.1-
>matbench@ git+https://github.com/materialsproject/matbench.git->mattext==0.0.1) (3.6.0)
Requirement already satisfied: cloudpickle>=1.2.1 in /usr/local/lib/python3.12/dist-packages (from submitit>=1.3.3->hydra-
submitit-launcher->mattext==0.0.1) (3.1.2)
Requirement already satisfied: mpmath<1.4,>=1.1.0 in /usr/local/lib/python3.12/dist-packages (from sympy>=1.13.3->torch-
>mattext==0.0.1) (1.3.0)
Requirement already satisfied: MarkupSafe>=2.0 in /usr/local/lib/python3.12/dist-packages (from jinja2->torch-
>mattext==0.0.1) (3.0.3)
Collecting spglib>=2.5.0 (from robocrys->xtal2txt->mattext==0.0.1)
Downloading spglib-2.7.0-cp312-cp312-manylinux_2_27_x86_64.manylinux_2_28_x86_64.whl.metadata (4.2 kB)
Requirement already satisfied: inflect in /usr/local/lib/python3.12/dist-packages (from robocrys->xtal2txt->mattext==0.0.1)
(7.5.0)
Collecting pubchempy (from robocrys->xtal2txt->mattext==0.0.1)
Downloading pubchempy-1.0.5-py3-none-any.whl.metadata (4.3 kB)
Collecting pybtex (from robocrys->xtal2txt->mattext==0.0.1)
Downloading pybtex-0.25.1-py2.py3-none-any.whl.metadata (2.2 kB)
Collecting m3gnet==0.2.4 (from slices->xtal2txt->mattext==0.0.1)
Downloading m3gnet-0.2.4-py3-none-any.whl.metadata (23 kB)
Collecting smact==2.5.5 (from slices->xtal2txt->mattext==0.0.1)
Downloading SMACT-2.5.5-py3-none-any.whl.metadata (13 kB)
Requirement already satisfied: tensorflow in /usr/local/lib/python3.12/dist-packages (from m3gnet==0.2.4->slices->xtal2txt-
>mattext==0.0.1) (2.19.0)
Collecting ase (from m3gnet==0.2.4->slices->xtal2txt->mattext==0.0.1)
Downloading ase-3.27.0-py3-none-any.whl.metadata (4.3 kB)
Collecting pathos (from smact==2.5.5->slices->xtal2txt->mattext==0.0.1)
Downloading pathos-0.3.4-py3-none-any.whl.metadata (11 kB)
Requirement already satisfied: aiohappyeyeballs>=2.5.0 in /usr/local/lib/python3.12/dist-packages (from
aiohttp!=4.0.0a0,!=4.0.0a1->fsspec[http]<=2025.3.0,>=2023.1.0->datasets->mattext==0.0.1) (2.6.1)
Requirement already satisfied: aiosignal>=1.4.0 in /usr/local/lib/python3.12/dist-packages (from aiohttp!=4.0.0a0,!=4.0.0a1-
>fsspec[http]<=2025.3.0,>=2023.1.0->datasets->mattext==0.0.1) (1.4.0)
Requirement already satisfied: attrs>=17.3.0 in /usr/local/lib/python3.12/dist-packages (from aiohttp!=4.0.0a0,!=4.0.0a1-
```

```
>fsspec[http]<=2025.3.0,>=2023.1.0->datasets->mattext==0.0.1) (25.4.0)
Requirement already satisfied: frozenlist>=1.1.1 in /usr/local/lib/python3.12/dist-packages (from aiohttp!=4.0.0a0,!=4.0.0a1-
>fsspec[http]<=2025.3.0,>=2023.1.0->datasets->mattext==0.0.1) (1.8.0)
Requirement already satisfied: multidict<7.0,>=4.5 in /usr/local/lib/python3.12/dist-packages (from
aiohttp!=4.0.0a0,!=4.0.0a1->fsspec[http]<=2025.3.0,>=2023.1.0->datasets->mattext==0.0.1) (6.7.0)
Requirement already satisfied: propcache>=0.2.0 in /usr/local/lib/python3.12/dist-packages (from aiohttp!=4.0.0a0,!=4.0.0a1-
>fsspec[http]<=2025.3.0,>=2023.1.0->datasets->mattext==0.0.1) (0.4.1)
Requirement already satisfied: yarl<2.0,>=1.17.0 in /usr/local/lib/python3.12/dist-packages (from aiohttp!=4.0.0a0,!=4.0.0a1-
>fsspec[http]<=2025.3.0,>=2023.1.0->datasets->mattext==0.0.1) (1.22.0)
Requirement already satisfied: smmap<6,>=3.0.1 in /usr/local/lib/python3.12/dist-packages (from gitdb<5,>=4.0.1-
>gitpython!=3.1.29,>=1.0.0->wandb->mattext==0.0.1) (5.0.2)
Collecting bibtexparser>=1.4.0 (from pymatgen>=2023->matminer>=0.7.4->matbench@
git+https://github.com/materialsproject/matbench.git->mattext==0.0.1)
Downloading bibtexparser-1.4.3.tar.gz (55 kB)
━━━━━━━━━━━━━━━━━━━━━━━━━━━━━━━━━━━━━━━━ 55.6/55.6 kB 2.2 MB/s eta 0:00:00
Preparing metadata (setup.py) ... done
Requirement already satisfied: matplotlib>=3.8 in /usr/local/lib/python3.12/dist-packages (from pymatgen>=2023-
>matminer>=0.7.4->matbench@ git+https://github.com/materialsproject/matbench.git->mattext==0.0.1) (3.10.0)
Requirement already satisfied: orjson<4,>=3.10 in /usr/local/lib/python3.12/dist-packages (from pymatgen>=2023-
>matminer>=0.7.4->matbench@ git+https://github.com/materialsproject/matbench.git->mattext==0.0.1) (3.11.5)
Collecting palettable>=3.3.3 (from pymatgen>=2023->matminer>=0.7.4->matbench@
git+https://github.com/materialsproject/matbench.git->mattext==0.0.1)
Downloading palettable-3.3.3-py2.py3-none-any.whl.metadata (3.3 kB)
Requirement already satisfied: plotly>=5.0.0 in /usr/local/lib/python3.12/dist-packages (from pymatgen>=2023-
>matminer>=0.7.4->matbench@ git+https://github.com/materialsproject/matbench.git->mattext==0.0.1) (5.24.1)
Requirement already satisfied: tabulate>=0.9 in /usr/local/lib/python3.12/dist-packages (from pymatgen>=2023-
>matminer>=0.7.4->matbench@ git+https://github.com/materialsproject/matbench.git->mattext==0.0.1) (0.9.0)
Collecting uncertainties>=3.1.4 (from pymatgen>=2023->matminer>=0.7.4->matbench@
git+https://github.com/materialsproject/matbench.git->mattext==0.0.1)
Downloading uncertainties-3.2.3-py3-none-any.whl.metadata (7.0 kB)
Collecting dnspython<3.0.0,>=1.16.0 (from pymongo~=4.5->matminer>=0.7.4->matbench@
git+https://github.com/materialsproject/matbench.git->mattext==0.0.1)
Downloading dnspython-2.8.0-py3-none-any.whl.metadata (5.7 kB)
Requirement already satisfied: six>=1.5 in /usr/local/lib/python3.12/dist-packages (from python-dateutil>=2.8.2->pandas-
>datasets->mattext==0.0.1) (1.17.0)
Collecting ruamel.yaml.clib>=0.2.15 (from ruamel.yaml->monty>=2022.4.26->matbench@
git+https://github.com/materialsproject/matbench.git->mattext==0.0.1)
Downloading ruamel_yaml_clib-0.2.15-cp312-cp312-
manylinux2014_x86_64.manylinux_2_17_x86_64.manylinux_2_28_x86_64.whl.metadata (3.5 kB)
Requirement already satisfied: more_itertools>=8.5.0 in /usr/local/lib/python3.12/dist-packages (from inflect->robocrys-
>xtal2txt->mattext==0.0.1) (10.8.0)
Requirement already satisfied: typeguard>=4.0.1 in /usr/local/lib/python3.12/dist-packages (from inflect->robocrys->xtal2txt-
>mattext==0.0.1) (4.4.4)
Collecting latexcodec>=1.0.4 (from pybtex->robocrys->xtal2txt->mattext==0.0.1)
Downloading latexcodec-3.0.1-py3-none-any.whl.metadata (5.2 kB)
Requirement already satisfied: pyparsing>=2.0.3 in /usr/local/lib/python3.12/dist-packages (from bibtexparser>=1.4.0-
>pymatgen>=2023->matminer>=0.7.4->matbench@ git+https://github.com/materialsproject/matbench.git->mattext==0.0.1)
(3.2.5)
Requirement already satisfied: contourpy>=1.0.1 in /usr/local/lib/python3.12/dist-packages (from matplotlib>=3.8-
>pymatgen>=2023->matminer>=0.7.4->matbench@ git+https://github.com/materialsproject/matbench.git->mattext==0.0.1)
(1.3.3)
Requirement already satisfied: cycler>=0.10 in /usr/local/lib/python3.12/dist-packages (from matplotlib>=3.8-
>pymatgen>=2023->matminer>=0.7.4->matbench@ git+https://github.com/materialsproject/matbench.git->mattext==0.0.1)
(0.12.1)
Requirement already satisfied: fonttools>=4.22.0 in /usr/local/lib/python3.12/dist-packages (from matplotlib>=3.8-
>pymatgen>=2023->matminer>=0.7.4->matbench@ git+https://github.com/materialsproject/matbench.git->mattext==0.0.1)
(4.61.1)
Requirement already satisfied: kiwisolver>=1.3.1 in /usr/local/lib/python3.12/dist-packages (from matplotlib>=3.8-
>pymatgen>=2023->matminer>=0.7.4->matbench@ git+https://github.com/materialsproject/matbench.git->mattext==0.0.1)
(1.4.9)
Requirement already satisfied: pillow>=8 in /usr/local/lib/python3.12/dist-packages (from matplotlib>=3.8-
>pymatgen>=2023->matminer>=0.7.4->matbench@ git+https://github.com/materialsproject/matbench.git->mattext==0.0.1)
(11.3.0)
Requirement already satisfied: tenacity>=6.2.0 in /usr/local/lib/python3.12/dist-packages (from plotly>=5.0.0-
>pymatgen>=2023->matminer>=0.7.4->matbench@ git+https://github.com/materialsproject/matbench.git->mattext==0.0.1)
(9.1.2)
Collecting ppft>=1.7.7 (from pathos->smact==2.5.5->slices->xtal2txt->mattext==0.0.1)
Downloading ppft-1.7.7-py3-none-any.whl.metadata (12 kB)
INFO: pip is looking at multiple versions of pathos to determine which version is compatible with other requirements. This
```

could take a while.
Collecting pathos (from smact==2.5.5->slices->xtal2txt->mattext==0.0.1)
Downloading pathos-0.3.3-py3-none-any.whl.metadata (11 kB)
Downloading pathos-0.3.2-py3-none-any.whl.metadata (11 kB)
Collecting pox>=0.3.4 (from pathos->smact==2.5.5->slices->xtal2txt->mattext==0.0.1)
Downloading pox-0.3.6-py3-none-any.whl.metadata (8.0 kB)
Requirement already satisfied: absl-py>=1.0.0 in /usr/local/lib/python3.12/dist-packages (from tensorflow->m3gnet==0.2.4->slices->xtal2txt->mattext==0.0.1) (1.4.0)
Requirement already satisfied: astunparse>=1.6.0 in /usr/local/lib/python3.12/dist-packages (from tensorflow->m3gnet==0.2.4->slices->xtal2txt->mattext==0.0.1) (1.6.3)
Requirement already satisfied: flatbuffers>=24.3.25 in /usr/local/lib/python3.12/dist-packages (from tensorflow->m3gnet==0.2.4->slices->xtal2txt->mattext==0.0.1) (25.9.23)
Requirement already satisfied: gast!=0.5.0,!=0.5.1,!=0.5.2,>=0.2.1 in /usr/local/lib/python3.12/dist-packages (from tensorflow->m3gnet==0.2.4->slices->xtal2txt->mattext==0.0.1) (0.7.0)
Requirement already satisfied: google-pasta>=0.1.1 in /usr/local/lib/python3.12/dist-packages (from tensorflow->m3gnet==0.2.4->slices->xtal2txt->mattext==0.0.1) (0.2.0)
Requirement already satisfied: libclang>=13.0.0 in /usr/local/lib/python3.12/dist-packages (from tensorflow->m3gnet==0.2.4->slices->xtal2txt->mattext==0.0.1) (18.1.1)
Requirement already satisfied: opt-einsum>=2.3.2 in /usr/local/lib/python3.12/dist-packages (from tensorflow->m3gnet==0.2.4->slices->xtal2txt->mattext==0.0.1) (3.4.0)
Requirement already satisfied: wrapt>=1.11.0 in /usr/local/lib/python3.12/dist-packages (from tensorflow->m3gnet==0.2.4->slices->xtal2txt->mattext==0.0.1) (2.0.1)
Requirement already satisfied: grpcio<2.0,>=1.24.3 in /usr/local/lib/python3.12/dist-packages (from tensorflow->m3gnet==0.2.4->slices->xtal2txt->mattext==0.0.1) (1.76.0)
Requirement already satisfied: tensorboard~=2.19.0 in /usr/local/lib/python3.12/dist-packages (from tensorflow->m3gnet==0.2.4->slices->xtal2txt->mattext==0.0.1) (2.19.0)
INFO: pip is looking at multiple versions of tensorflow to determine which version is compatible with other requirements. This could take a while.
Collecting tensorflow (from m3gnet==0.2.4->slices->xtal2txt->mattext==0.0.1)
Downloading tensorflow-2.20.0-cp312-cp312-manylinux_2_17_x86_64.manylinux2014_x86_64.whl.metadata (4.5 kB)
Collecting tensorboard~=2.20.0 (from tensorflow->m3gnet==0.2.4->slices->xtal2txt->mattext==0.0.1)
Downloading tensorboard-2.20.0-py3-none-any.whl.metadata (1.8 kB)
Collecting tensorflow (from m3gnet==0.2.4->slices->xtal2txt->mattext==0.0.1)
Downloading tensorflow-2.19.1-cp312-cp312-manylinux_2_17_x86_64.manylinux2014_x86_64.whl.metadata (4.1 kB)
Downloading tensorflow-2.18.1-cp312-cp312-manylinux_2_17_x86_64.manylinux2014_x86_64.whl.metadata (4.1 kB)
Collecting tensorboard<2.19,>=2.18 (from tensorflow->m3gnet==0.2.4->slices->xtal2txt->mattext==0.0.1)
Downloading tensorboard-2.18.0-py3-none-any.whl.metadata (1.6 kB)
Collecting tensorflow (from m3gnet==0.2.4->slices->xtal2txt->mattext==0.0.1)
Downloading tensorflow-2.18.0-cp312-cp312-manylinux_2_17_x86_64.manylinux2014_x86_64.whl.metadata (4.1 kB)
Downloading tensorflow-2.17.1-cp312-cp312-manylinux_2_17_x86_64.manylinux2014_x86_64.whl.metadata (4.2 kB)
Requirement already satisfied: h5py>=3.10.0 in /usr/local/lib/python3.12/dist-packages (from tensorflow->m3gnet==0.2.4->slices->xtal2txt->mattext==0.0.1) (3.15.1)
Collecting ml-dtypes<0.5.0,>=0.3.1 (from tensorflow->m3gnet==0.2.4->slices->xtal2txt->mattext==0.0.1)
Downloading ml_dtypes-0.4.1-cp312-cp312-manylinux_2_17_x86_64.manylinux2014_x86_64.whl.metadata (20 kB)
Collecting protobuf!=4.21.0,!=5.28.0,<7,>=3.19.0 (from wandb->mattext==0.0.1)
Downloading protobuf-4.25.8-cp37-abi3-manylinux2014_x86_64.whl.metadata (541 bytes)
Collecting tensorboard<2.18,>=2.17 (from tensorflow->m3gnet==0.2.4->slices->xtal2txt->mattext==0.0.1)
Downloading tensorboard-2.17.1-py3-none-any.whl.metadata (1.6 kB)
Collecting tensorflow (from m3gnet==0.2.4->slices->xtal2txt->mattext==0.0.1)
Downloading tensorflow-2.17.0-cp312-cp312-manylinux_2_17_x86_64.manylinux2014_x86_64.whl.metadata (4.2 kB)
Downloading tensorflow-2.16.2-cp312-cp312-manylinux_2_17_x86_64.manylinux2014_x86_64.whl.metadata (4.2 kB)
Collecting ml-dtypes~=0.3.1 (from tensorflow->m3gnet==0.2.4->slices->xtal2txt->mattext==0.0.1)
Downloading ml_dtypes-0.3.2-cp312-cp312-manylinux_2_17_x86_64.manylinux2014_x86_64.whl.metadata (20 kB)
Collecting tensorboard<2.17,>=2.16 (from tensorflow->m3gnet==0.2.4->slices->xtal2txt->mattext==0.0.1)
Downloading tensorboard-2.16.2-py3-none-any.whl.metadata (1.6 kB)
INFO: pip is still looking at multiple versions of tensorflow to determine which version is compatible with other requirements. This could take a while.
Collecting tensorflow (from m3gnet==0.2.4->slices->xtal2txt->mattext==0.0.1)
Downloading tensorflow-2.16.1-cp312-cp312-manylinux_2_17_x86_64.manylinux2014_x86_64.whl.metadata (4.3 kB)
ERROR: Cannot install m3gnet, mattext and tensorflow==2.19.0 because these package versions have conflicting dependencies.

The conflict is caused by:
xtal2txt 0.1.0 depends on keras<3
tensorflow 2.19.0 depends on keras>=3.5.0
xtal2txt 0.1.0 depends on keras<3
tensorflow 2.20.0 depends on keras>=3.10.0
xtal2txt 0.1.0 depends on keras<3
tensorflow 2.19.1 depends on keras>=3.5.0

xtal2txt 0.1.0 depends on keras<3
tensorflow 2.18.1 depends on keras>=3.5.0
xtal2txt 0.1.0 depends on keras<3
tensorflow 2.18.0 depends on keras>=3.5.0
xtal2txt 0.1.0 depends on keras<3
tensorflow 2.17.1 depends on keras>=3.2.0
xtal2txt 0.1.0 depends on keras<3
tensorflow 2.17.0 depends on keras>=3.2.0
xtal2txt 0.1.0 depends on keras<3
tensorflow 2.16.2 depends on keras>=3.0.0
xtal2txt 0.1.0 depends on keras<3
tensorflow 2.16.1 depends on keras>=3.0.0

To fix this you could try to:
1. loosen the range of package versions you've specified
2. remove package versions to allow pip to attempt to solve the dependency conflict

ERROR: ResolutionImpossible: for help visit https://pip.pypa.io/en/latest/topics/dependency-resolution/#dealing-with-dependency-conflicts

Version 1:

Reviewer comments:

Reviewer #2

(Remarks to the Author)
The authors have addressed all our comments, and only minor typographical errors remain, which do not require further review:

- Figure R1 (or Fig. 8) caption: "(Right) A monotonically increasing curve yields a negative CC-Cliff, indicating that the models struggle more with coordinate information than categorical information." → "coordinate" and "categorical" should be inverted.
- "indicaing" → "indicating"
- "would indicate a models inability" → "a model's inability"
- Fig. 2: "(Lcat,α). Which" → ", which"
- "MODNet, which relies on composition-derived descriptors with limited geometric sensitivity" → This is not strictly composition-derived; structural features are likely involved as well.
- "Dataset 3, which is more structurally constrained dataset compared to 1 and 2" → "which is a more structurally constrained dataset..."
- "synthetic datasets described in are" → Missing context: "in what?"
- The Matra Genoa citation requires volume and page numbers.
- A period is missing at the end of the caption of Fig. 1.
- "e.e." → "e.g." (p. 2)
- Commas are missing after "i.e." or "e.g." in multiple instances (e.g., p. 3).
- The label (A) is missing in the caption of Fig. 2.
- In Fig. 2, there is an extra space after a parenthesis in subfigure A.
- "that dependent" → "that depends" or "that are dependent."
- "Our findings... further reveals" → "reveal"
- Fig. 4 caption: "the framework also supportS" → "supports"
- "Appendix appendix" → "Appendix" (redundant)
- Fig. 7 caption: The period is incorrectly spaced far from the caption text.

(Remarks on code availability)

Reviewer #3

(Remarks to the Author)
The authors have addressed all our comments, and only minor typographical errors remain, which do not require further review:

- Figure R1 (or Fig. 8) caption: "(Right) A monotonically increasing curve yields a negative CC-Cliff, indicating that the models struggle more with coordinate information than categorical information." → "coordinate" and "categorical" should be inverted.
- "indicaing" → "indicating"
- "would indicate a models inability" → "a model's inability"
- Fig. 2: "(Lcat,α). Which" → ", which"
- "MODNet, which relies on composition-derived descriptors with limited geometric sensitivity" → This is not strictly

composition-derived; structural features are likely involved as well.
- "Dataset 3, which is more structurally constrained dataset compared to 1 and 2" → "which is a more structurally constrained dataset..."
- "synthetic datasets described in are" → Missing context: "in what?"
- The Matra Genoa citation requires volume and page numbers.
- A period is missing at the end of the caption of Fig. 1.
- "e.e." → "e.g." (p. 2)
- Commas are missing after "i.e." or "e.g." in multiple instances (e.g., p. 3).
- The label (A) is missing in the caption of Fig. 2.
- In Fig. 2, there is an extra space after a parenthesis in subfigure A.
- "that dependent" → "that depends" or "that are dependent."
- "Our findings... further reveals" → "reveal"
- Fig. 4 caption: "the framework also supportS" → "supports"
- "Appendix appendix" → "Appendix" (redundant)
- Fig. 7 caption: The period is incorrectly spaced far from the caption text.

(Remarks on code availability)

Reviewer #4

(Remarks to the Author)
We thank the authors for taking time to address our comments. We have no further questions. We are happy to recommend the current manuscript as it is.

(Remarks on code availability)
I have checked the code. It is now running with no issue.

Open Access This Peer Review File is licensed under a Creative Commons Attribution 4.0 International License, which permits use, sharing, adaptation, distribution and reproduction in any medium or format, as long as you give appropriate credit to the original author(s) and the source, provide a link to the Creative Commons license, and indicate if changes were made.
In cases where reviewers are anonymous, credit should be given to 'Anonymous Referee' and the source.
The images or other third party material in this Peer Review File are included in the article’s Creative Commons license, unless indicated otherwise in a credit line to the material. If material is not included in the article’s Creative Commons license and your intended use is not permitted by statutory regulation or exceeds the permitted use, you will need to obtain permission directly from the copyright holder.
To view a copy of this license, visit https://creativecommons.org/licenses/by/4.0/

RESPONSE TO THE REVIEWERS

REVIEWERS 1 AND 4

Reviewer Point P 1.1 — This paper addresses the important and timely topic of the use of LLMs in scientific machine learning, focusing on the impact of coordinate data on the prediction performance. The authors present a comprehensive analysis by designing an original approach to assess the sensitivity of an LLM to coordinate-categorical data. Their publicly available and extensive benchmarking framework is a notable step in a more responsible use of LLMs in materials science and further demonstrates that LLMs are ill-suited for tasks primarily governed by structural properties. The manuscript is well-structured, with clear methodology and thorough discussion. It provides meaningful insights into the use of LLMs in materials science. While the study is robust, revisions are needed to further strengthen and clarify the paper.

Reply: *We sincerely thank the reviewer for the feedback that strenghtend our work and the positive assessment of our manuscript. We have addressed all points raised in the review. The major changes in the revised manuscript are:*

- *We extended the CC-Cliff analysis to include* `COGN`[1] *and* `MODNet`,[2] *providing a direct comparison of how language models, graph neural networks, and feature-based models differ in their sensitivity to coordinate versus category information (new fig. R2C).*
- *We added Robocrystallographer as an additional representation across all our scaling experiments, including dataset size scaling and model size scaling (updated fig. R4 and fig. R5).*
- *We clarified the claims and several typographic and citation issues raised by the reviewer.*

Reviewer Point P 1.2 — Either we got it completely wrong or the ticks on the x-axis of Figure 2B are inverted, especially given that the caption explicitly states "Lcoord > Lcat." Please verify and correct this if necessary. On the contrary, please clarify.

Reply: *The reviewer is correct. The L_{coord} and L_{cat} axes were inadvertently swapped in Figure 2B. We have corrected this in the revised manuscript.*

Reviewer Point P 1.3 — It would be helpful for the reader if the authors could schematize and provide an interpretation of the different cases for the CC-cliff, such as CC-cliff = 0 (e.g., distinguishing a straight horizontal line from a V- or U-shape), as well as negative values.

Reply: *We have added a dedicated appendix section with an accompanying schematic figure (see Figure R1) that systematically illustrates the four qualitatively distinct cases.*

The newly added sections now reads:

> ***Interpretation of CC-Cliff Values***
>
> *The CC-Cliff metric is defined as* $\text{CC-Cliff} = L_{\text{coord},\alpha} - L_{\text{cat},\alpha}$*, where both* $L_{\text{coord},\alpha}$ *and* $L_{\text{cat},\alpha}$ *measure how much the model's error deviates from the balanced (*$\alpha = 0.5$*) baseline in the coordinate-dominated and category-dominated regimes, respectively. Different values of the CC-Cliff correspond to qualitatively distinct learning behaviors, illustrated schematically in Figure R1:*

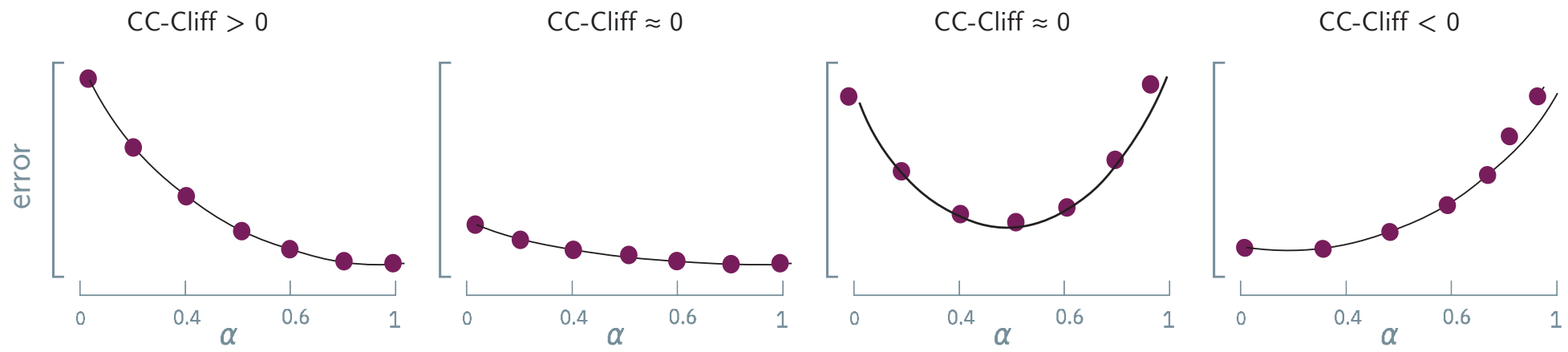

Figure R1: Schematic illustration of qualitatively distinct CC-Cliff profiles. Each panel shows an idealized loss curve as a function of the mixing parameter α, which interpolates between coordinate-dominated ($\alpha = 0$) and category-dominated ($\alpha = 1$) targets. (*Left*) A monotonically decreasing loss curve yields a large positive CC-Cliff, indicating the model struggles with coordinate information — the dominant pattern observed for LLMs. (*Centre-left*) A flat curve yields CC-Cliff ≈ 0 with both contributions near zero, indicating the model is insensitive to the nature of the input signal. (*Centre-right*) A U-shaped curve also yields CC-Cliff ≈ 0, but here both contributions are positive and symmetric, indicating the model benefits from a balance of both signal types. (*Right*) A monotonically increasing loss curve yields a negative CC-Cliff, indicating that the models struggle more with coordinate information than categorical information.

POSITIVE CC-CLIFF ($L_{coord,\alpha} \gg L_{cat,\alpha}$). *The model struggles substantially more with coordinate-dominated tasks than with category-dominated ones. The underlying loss decreases monotonically as α increases from 0 to 1. The model performs poorly when the target depends heavily on positional information and well when it depends on categorical information. This is the dominant pattern we observe for LLMs across all experiments.*

CC-CLIFF ≈ 0: FLAT LOSS CURVE. *Both $L_{coord,\alpha} \approx 0$ and $L_{cat,\alpha} \approx 0$. The model's error is approximately constant across the full range of α, indicaing that the model's error is independent on whether the ground truth depends mostly on coordinate or categorical information.*

CC-CLIFF ≈ 0: U- OR V-SHAPED LOSS CURVE. *This case is distinct from the flat curve above, despite yielding the same CC-Cliff value. Here, $L_{coord,\alpha} > 0$ and $L_{cat,\alpha} > 0$, but symmetrically, so their difference is near zero. The model performs best in the balanced regime ($\alpha = 0.5$) and degrades at both extremes, suggesting it benefits from having* both *types of information simultaneously, but struggles when either dominates exclusively. This case would indicate a models inability to operate well when the signal is one-sided.*

NEGATIVE CC-CLIFF ($L_{cat,\alpha} \gg L_{coord,\alpha}$). *The model struggles more with category-dominated tasks than coordinate-dominated ones. The loss curve increases monotonically with α, reflecting better performance when coordinates dominate. This pattern could arise in models with strong geometric inductive biases applied to tasks where categorical variation is high but geometric variation is constrained. We do not observe this regime for LLMs in our experiments. Our analysis with* `COGN`[1] *and* `MODNet`[2] *(see Section 2.1) shows that GNN-based models can exhibit reduced CC-Cliff values in some datasets.*

Reviewer Point P 1.4 — Regarding the equation E=Ecoord+Ecat for different values of α, it seems important to ensure that E does not vary too much with respect to α. Without this, if Ecoord and Ecat values are on very different orders of magnitude, increasing the weight of Ecoord in E could trivially lead to a higher E even if Ecat is reduced, potentially resulting in a larger error compared to a balanced 50-50 case. A

normalization step might be necessary to make the reasoning robust, unless this has already been implemented or taken into account in some way (in which case a few words should be added in the text).

Reply: *We confirm that normalization was indeed applied, both E_{cat} and E_{coord} are independently standardized to zero mean and unit variance before the α-weighted mixing. We have added a clarifying sentence to Section 4.1.1 of the revised manuscript which reads,*

> *Prior to mixing, both $E_{category}$ and $E_{coordinate}$ are independently standardized to zero mean and unit variance across the dataset, ensuring that α controls the relative information contribution rather than differences in raw energy scales.*

Reviewer Point P 1.5 — The methodology for computing the CC-cliff should ideally also be applied using one GNN and one other intermediate model (typically MODNet) to further investigate the shape of the cliff, particularly in light of Figure 7 (in order to have the CC-cliff for at least one blue, one red, and one grey circles). This would help differentiate the advantages and limitations of GNNs, LLMs, ANNs, and other models more clearly.

Reply:

We have extended the CC-cliff analysis to include `GNN`-based (`COGN`[1]), and `Feature`-based (`MODNet`[2] features), in addition to `Transformer`-based modelling approach. Figure R2C shows a single dumbbell per model type with contributions averaged across all six datasets. The updated figure with caption is below

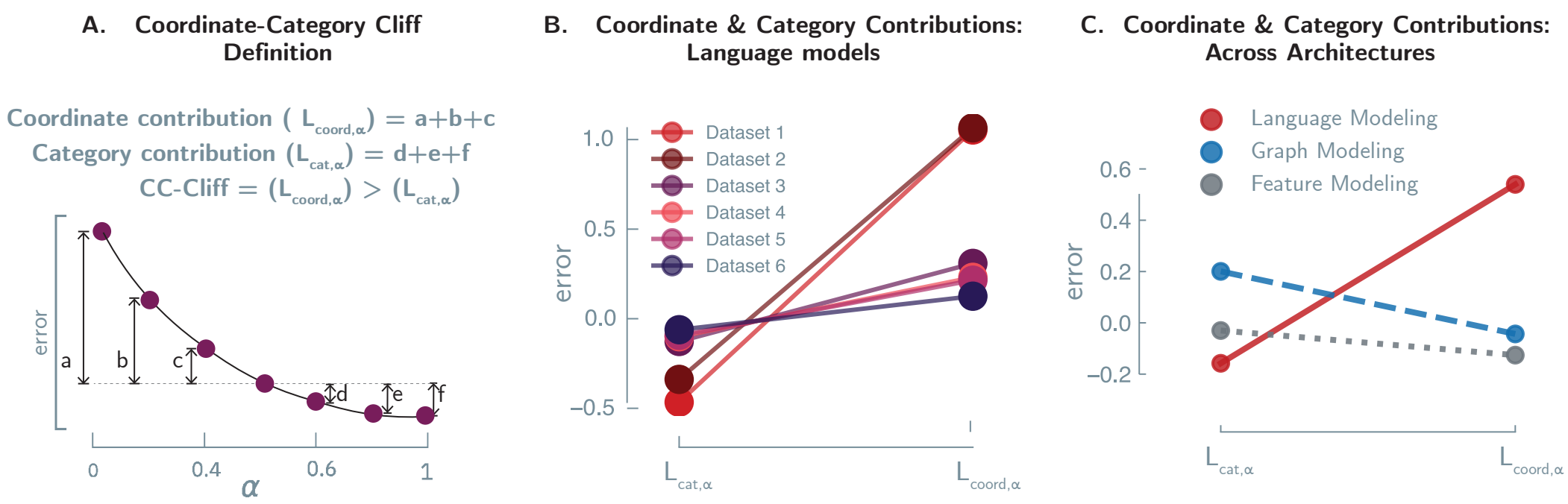

Figure R2: The ability to model categorical vs. coordinate data depends on the model architecture and language models have a preference for categorical data. The coordinate contribution and category contribution are defined as: $L_{coord,\alpha} = \sum_{\alpha\in\alpha_g} \text{loss}(\alpha) - 3 \times \text{loss}(0.5)$ and $L_{cat,\alpha} = \sum_{\alpha\in\alpha_c} \text{loss}(\alpha) - 3 \times \text{loss}(0.5)$, where $\alpha_g = \{0, 0.2, 0.4\}$ and $\alpha_c = \{0.6, 0.8, 1.0\}$. Accounting for the three α values included in each sum we subtract loss(0.5) three times (which effectively means we subtract the loss at α=0.5 from each individual loss contributing to the sum). **B** Coordinate and Category contributions for a language model. For all six different datasets, the language model shows a positive Coordinate-Category Cliff (CC-Cliff), i.e., coordinate contribution ($L_{coord,\alpha}$) > category contribution ($L_{cat,\alpha}$). Which is a gap in learning coordinate computations compared to category computations. There is also variation in the cliff magnitude across different datasets. **C** The $L_{coord,\alpha}$ and $L_{cat,\alpha}$ contributions averaged across all property datasets for `Transformer`-based , `GNN`-based (`COGN`[1]), and `Feature`-based (`MODNet`[2] features). A positive slope indicates a larger CC-cliff.

We find that `COGN`[1] and `MODNet`[2] exhibit markedly different CC-cliff profiles.
We furhter added the folowing text in Section 2.1 explaining panel c of this figure,

To contextualize these findings across model classes, Figure R2C shows the composition and geometry contributions averaged across all property datasets for three architectures. Language modeling approach exhibits a large positive slope — low composition contribution and high geometry contribution. Graph modeling approach based on `COGN`[1] shows the opposite pattern: a negative slope indicating that the GNN handles coordinate-dominated tasks comparatively easier than category-dominated ones, reflecting its geometric inductive bias. The descriptor-based model `MODNet`[2] lies near zero, exhibiting balanced sensitivity to both information types. This comparison directly illustrates that the CC-cliff is not an inherent property of the task, but a consequence of model architecture and inductive bias (see Appendix section 5.2 for more discussion).

corresponding appendix section is below,

CC-Cliff across model architectures

To compare the CC-Cliff phenomenon beyond LLMs, we extend the analysis to include a graph neural network (`COGN`[1]) and a composition-featurized neural network (`MODNet`[2]). Both models are trained on the same synthetic dataset used for the language modelling experiments (see Section 2.1), across all seven mixing parameters $\alpha \in 0, 0.2, 0.4, 0.5, 0.6, 0.8, 1.0$ and all using the same train/test splits.

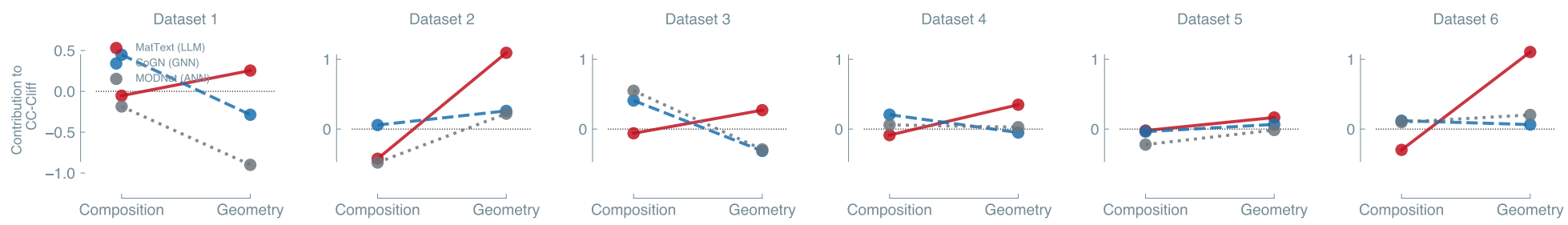

Figure R3: Per-dataset composition and geometry contributions to the CC-Cliff for different architectures. Each subplot corresponds to one dataset. Dumbbells connect the composition contribution ($L_{category}$, left endpoint) to the geometry contribution ($L_{coordinate}$, right endpoint) for each model class.

The per-dataset CC-Cliff values for all three model classes are shown in Figure R2C (averaged) and Figure R3 (per dataset). `COGN`, which explicitly encodes positions and inter distances in its graph representation, exhibits markedly reduced CC-Cliff values compared to LLMs across all datasets. `MODNet`, which relies on composition-derived descriptors with limited geometric sensitivity, shows intermediate behaviour. These results indicate that the CC-Cliff is not an intrinsic property of the task, but reflects the degree to which a model's representation captures coordinate information.

We use the `COGN`[1] implementation from `kgcnn` v3.1.0 with the default architecture (`model_default`). Crystal structures are converted to graphs using a k-nearest neighbour preprocessor with $k = 24$ (`KNNAsymmetricUnitCell`). Models are trained for up to 800 epochs using the Adam optimizer with a polynomial learning rate decay schedule (initial rate 5×10^{-5}, final rate 10^{-5}), MAE loss, and gradient clipping (norm clip 0.5, value clip 0.1) to ensure stability. Early stopping with patience 50 is applied.

We use `MODNet`[2] v0.4.5 with composition and structure features computed via the `DeBreuck2020Featurizer` (`fast_oxid=True`). For each α-target, the top 100 features are selected by mutual information with the training targets. The model architecture comprises four fully connected layers with $[256, 128, 64, 32]$ neurons and ELU activations. Training uses Adam with learning rate 10^{-3}, batch size 64, and 200 epochs.

We thank the reviewer for this suggestion, with this addition we think our findings are better conveyed.

Reviewer Point P 1.6 — In Section 2.2, our understanding of Figure 3 is that, for datasets 1 and 2, including coordinate data does not significantly impact the results, as the two solid lines are very close. In contrast, dataset 3 exhibits the behavior that we expected, where the importance of coordinate data becomes more pronounced as the number of bins increases, as evidenced by the high CC-cliff for category-only data. The authors do not discuss these differences (suggesting a potential explanation). Additional description and interpretation of these observations would definitely strengthen this section. Additionally, it should be clarified that the inputs of the LLMs are not affected by the binning process.

Reply: *We have added explanation int the main text Section 2.2 addressing both points. The paragraph reads,*

> *For datasets 1 and 2, the two solid lines remain close across all bin resolutions, indicating that the LLM effectively ignores coordinate information regardless of its presence in the input. Dataset 3, which is more structurally constrained dataset compared to 1 and 2, shows the expected behavior: at low bin resolution, composition alone approximates the coordinate-derived target, but as bins increase and finer geometric resolution is required, this approximation breaks down and the CC-cliff rises for the category-only model.*

we have also added a line clarifying about the inputs to the LLM

> *We also note that the binning process affects only the target labels; the text inputs to the LLMs remain unchanged across all bin resolutions.*

Reviewer Point P 1.7 — The meaning of the dashed lines in Figure 6 is not specified in the caption. Please clarify this.

Reply: *We have added a clarifying sentence to the caption of Figure 6, which reads,*

> *Within each color group (compositional, local, geometric), different linestyles (solid, dotted) distinguish individual representations, robocrys is highligthed with grey colour since apart from being local representation it is a representation based on natural language explanation. The dotted black line is a reference to no change.*

Reviewer Point P 1.8 — Though they are material-specific, it might be appropriate to mention approaches in which the coordinates are translated into text using Wyckoff positions (as in Wren [Goodall et al., Sci. Adv. 8, eabn4117 (2022)] or Matra-Genoa [https://arxiv.org/abs/2501.16051]). Indeed, such approaches might alleviate some of the challenges associated with handling coordinates by categorizing them. Has there been any investigation into such material-text translations in the context of MatText or the present paper?

Reply:
We have expanded our related work section, which now includes

> *An alternative strategy for handling coordinate information is to convert it into categorical descriptors via crystallographic symmetry, as in Goodall et al.[3], which encodes structures using Wyckoff positions, and De Breuck et al.[4], which adopts a similar approach. Such representations effectively discretize geometric information, potentially making it more accessible to language models—a hypothesis supported by the competitive performance of our Local-Env representation, which also employs Wyckoff labels (see Appendix section 5.1).*

The Local-Env representation in MatText already employs Wyckoff labels: each local environment is prefixed with the space group symbol followed by the Wyckoff label for each site (e.g., `R3m / Ta (1a) ...`*). Hence we did not look into similar representations which would have been equally important but should have similar charecteristics.*

The performance of Local-Env in our experiments is consistent with the reviewer's intuition: by categorizing coordinates through Wyckoff labels rather than encoding raw fractional positions, the representation converts geometric information into a form more accessible to LLMs. Local-Env performs competitively and in several cases better than representations that include explicit coordinate information (e.g., CIF P_1), supporting the broader thesis of this work.

Reviewer Point P 1.9 — For clarity, it would be beneficial to maintain consistency in terminology throughout the manuscript. For example, avoid using "train/-trained/training" when referring to "fine-tune/fine-tuned/fine-tuning," as this could introduce ambiguity regarding different training stages.

Reply: *We have reviewed the manuscript and standardized the terminology, replacing "train/trained/training" with "fine-tune/fine-tuned/fine-tuning" wherever it refers to the fine-tuning stage, to clearly distinguish it from the pretraining stage.*

Reviewer Point P 1.10 — The term "labels" is occasionally used to refer to the "scalar target values" which could be confusing since the categorical data is also sometimes referred to as categorical labels. Clarifying or standardizing this terminology would improve readability.

Reply: *We thank the reviewer for this observation. We have standardized the terminology throughout the manuscript, replacing "labels" with "target values" wherever it refers to scalar regression outputs (e.g., energies), while retaining "categorical labels" exclusively for discrete type information.*

Reviewer Point P 1.11 — There are minor formatting issues to address: a missing parenthesis in the caption of Figure 2 (Lcat,α), and a missing final period at the end of the caption of Figure 7.

Reply: *We have fixed these formatting issues in our revised version of the manuscript.*

Reviewer Point P 1.12 — On page 10, the statement "there is no effect of either scaling dataset size or model parameter count" seems inconsistent with Figure 6, which shows improvements in RMSE of up to 10% and even 50%. This sentence should be clarified, possibly by quantifying the observed effects.

Reply: *The original phrasing was too strong. We have revised the text on to discuss dataset and model scaling with quantification. The revised text now reads,*

> *We find that, for all representations and prediction targets we investigated, dataset scaling (Figure R5, top row) yields minimal changes in RMSE (less than 20%, e.g., an absolute shift of $<$0.04 for a baseline RMSE of 0.2). For model scaling (Figure R5, bottom row), changes are similarly modest for μ and K. For both scaling approaches E_f shows larger reductions around ~20% with Robocrystallographer representation in data scaling and up to ~50% for some geometric representations in model scaling, though these are not consistent across representations. Importantly, even accounting for these improvements, the absolute performance of LLMs remains far below GNN-based approaches (See Figure 7).*

Reviewer Point P 1.13 — 1. When trying out MatText, I got the following package error despite following the proposed installation guidelines and running the "pip

install e." with no error. I could not find it in packages supported by PyPI or conda. Including how to install this package would be very useful.

ModuleNotFoundError Traceback (most recent call last) Cell In[1], line 1
—-> 1 from mattext.representations import TextRep
2 from pymatgen.core.structure import Structure
......

2. Also while you mentioned in `https://github.com/lamalab-org/MatText/blob/main/pyproject.toml` that MatText supports Python 3.12, I could not install it successfully until I downgraded the Python version to Python 3.9. Please double check this and confirm if it can be truly installed with Python 3.12 with no errors. The error log is added below

Obtaining file:///content/mattext
Installing build dependencies ... done
Checking if build backend supports build_editable ... done
Getting requirements to build editable ... done
...

To fix this you could try to
1. loosen the range of package versions you've specified
2. remove package versions to allow pip to attempt to solve the dependency conflict

ERROR: ResolutionImpossible: for help visit `https://pip.pypa.io/en/latest/topics/dependency-resolution/#dealing-with-dependency-conflicts`

Reply:
We thank the reviewer for identifying these installation hurdles. We have addressed these issues as follows:

- ***Installation Fix:*** *We resolved the installation issue and have updated our package. As suggested, we loosened the dependency requirements to resolve the* `ResolutionImpossible` *errors. We still had to pin certain versions of* `tensorflow, numpy and keras` *due to our dependency on* ***slices*** *package*
- ***CI Testing:*** *We found that certain downstream dependencies do not yet fully support Python 3.12, causing the* `ResolutionImpossible` *error. We have updated our metadata to* ***officially support Python 3.10 and 3.11****, and we now test these versions via a new CI pipeline.*

REVIEWERS 2 AND 3

Reviewer Point P 2.1 — This work studies the limitations and strengths of using text/string-based representations with LLMs for materials property prediction. Through extensive and carefully designed experiments, the authors demonstrate that, regardless of model size, dataset size, or text representation strategy, LLMs fail to capture coordinate-level information while excelling at learning categorical patterns. Consequently, this study suggests using GNN–based approaches for tasks dominated by structural effects, and LLMs for tasks where categorical or compositional information is sufficient. Furthermore, the authors introduce MatText, a Python package that takes a CIF file as input and outputs multiple text representations of materials suitable for training LLMs or other text-based methods. MatText can also serve as a framework for end-to-end language modeling of materials, including data processing, model training, and results analysis. The paper is well written, structured, and easy to follow. Overall, this is a solid piece of work. It addresses critical challenges in LLM- and string-based materials property prediction. The experiments are thorough, and the results are informative. I particularly appreciate the effort the authors have put into the experimental design and results analysis. It will certainly be of interest to the computational materials science community.

Reply: *We sincerely thank the reviewer for their positive assessment and review of our manuscript.*

Reviewer Point P 2.2 — In section 4.3, authors wrote that "There has been no tool to systematically derive text representations for materials.", I found this to be a strong/unfair claim given other efforts that had been made prior to this study, such as Robocrystallographer and MOFid tools. Please clarify.

Reply: *We have revised the sentence to acknowledge prior efforts and not make this strong claim, this section now reads*

> *While tools such as Robocrystallographer[5] and MOFid[6] exist for specific purposes, no unified framework has been available to systematically derive and benchmark multiple text representations across a broad range of crystalline materials.*

Reviewer Point P 2.3 — 2. What does the synthetic data you made in Section 2.1 look like? It would be useful to include one or two examples in the manuscript.

Reply: *We have added examples of synthetic data points to a new Appendix section (Appendix section 5.4), showing the same data point in two representations. Which is quoted below,*

> ### Examples of synthetic data
>
> *The synthetic datasets described in are constructed from materials-inspired configurations with generic type labels replacing element symbols. Below we show the same data point in two different representations, along with its target value $E(\alpha)$ at three mixing ratios for that particular example.*

Example: Category-based & Category-Coordinate based representations

- `A B C C D D D D D D  6.14 6.14 6.14 47 47 47`
- ```
  6.1 6.1 6.1
  47 47 47
  A 0.00 0.00 0.00
  B 0.50 0.50 0.50
  C 0.76 0.76 0.76
  C 0.24 0.24 0.24
  D 0.73 0.49 0.05
  D 0.05 0.73 0.49
  D 0.51 0.95 0.27
  ...
  ```

The corresponding target values are $E(\alpha{=}0) = 0.06$, $E(\alpha{=}0.5) = 0.13$, *and* $E(\alpha{=}1) = 0.20$.

Reviewer Point P 2.4 — 3. In section 4.4, authors mentioned that they have created pretraining datasets with varying datasize? How were these datasets created? I found little to no details on how they were created. Including these details would be very useful for reproducibility purposes.

Reply: *We have added a paragraph to Section 4.4 describing the dataset construction, which now reads,*

> *The pretraining datasets are derived from the NOMAD[7] database, queried via scripts from MatSciML,[8] filtering for 3D crystal structures for which geometry optimization was performed using the PBE functional and the VASP code. Duplicate structures were removed based on CIF symmetry. From the resulting pool of ~2M unique structures, we randomly sampled subsets of 30K, 100K, 300K, and 2M structures. For each subset, all nine text representations were derived using the MatText framework, yielding 36 pretraining datasets in total.*

Reviewer Point P 2.5 — 4. I believe that MatText also supports Robocrystallographer crystal text descriptions, why were the results with this input representation not compared with other text representations, for example in Figure 5 and 6?

Reply: *We have now added Robocrystallographer as a representation across all experiments, including the 30K comparison (Figure R4) and both dataset and model scaling analyses (Figure R5). Robocrystallographer is grouped with the local-geometry representations (alongside SLICES and Local-Env), consistent with its inductive bias of encoding local coordination environments in natural language rather than explicit fractional coordinates.*

The updated figure with caption is below
  ```

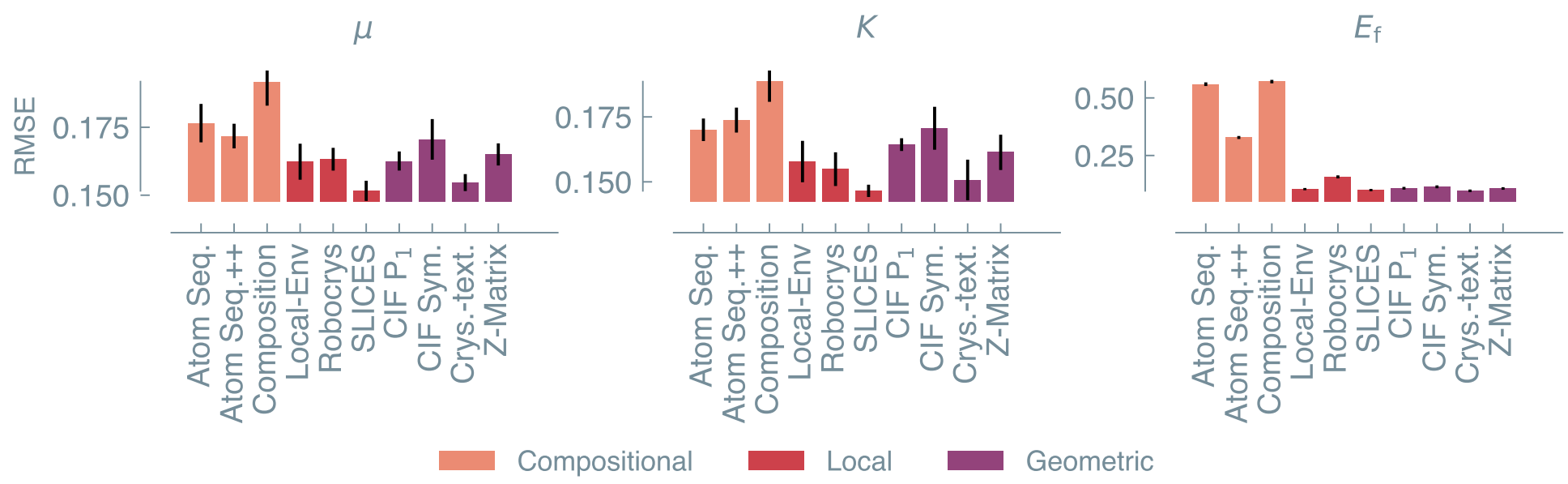

Figure R4: Predictive performance of large language models (LLMs) in materials property prediction tasks (shear modulus (μ), bulk modulus (K), and perovskite formation energy (E_f)) for different representations. Performance is measured by Root Mean Square Error (RMSE), with lower values indicating better performance. Representations are grouped by type: compositional-based (orange), local environment-based (red), and geometry-based (purple). Across all three properties, local environment based representations generally achieve the best performance, while explicit geometric representations show limited improvement or even degraded performance. Notably, the SLICES representation, which lacks explicit coordinate information, performs comparably to geometry-aware representations like Cif P_1, suggesting that current LLMs do not effectively leverage explicit coordinate information for materials property prediction. Robocrystallographer, which encodes local coordination environments in natural language rather than explicit coordinates, performs comparably to SLICES and Local-Env. The error bars indicate the standard deviation across five-fold cross-validation. A notable exception is the perovskites dataset, where there is a big difference between representation. This dataset has few unique chemical environments compared to the shear and bulk modulus datasets (see Figure 13 in appendix), wherefore most of the variance cannot be explained using composition information alone.

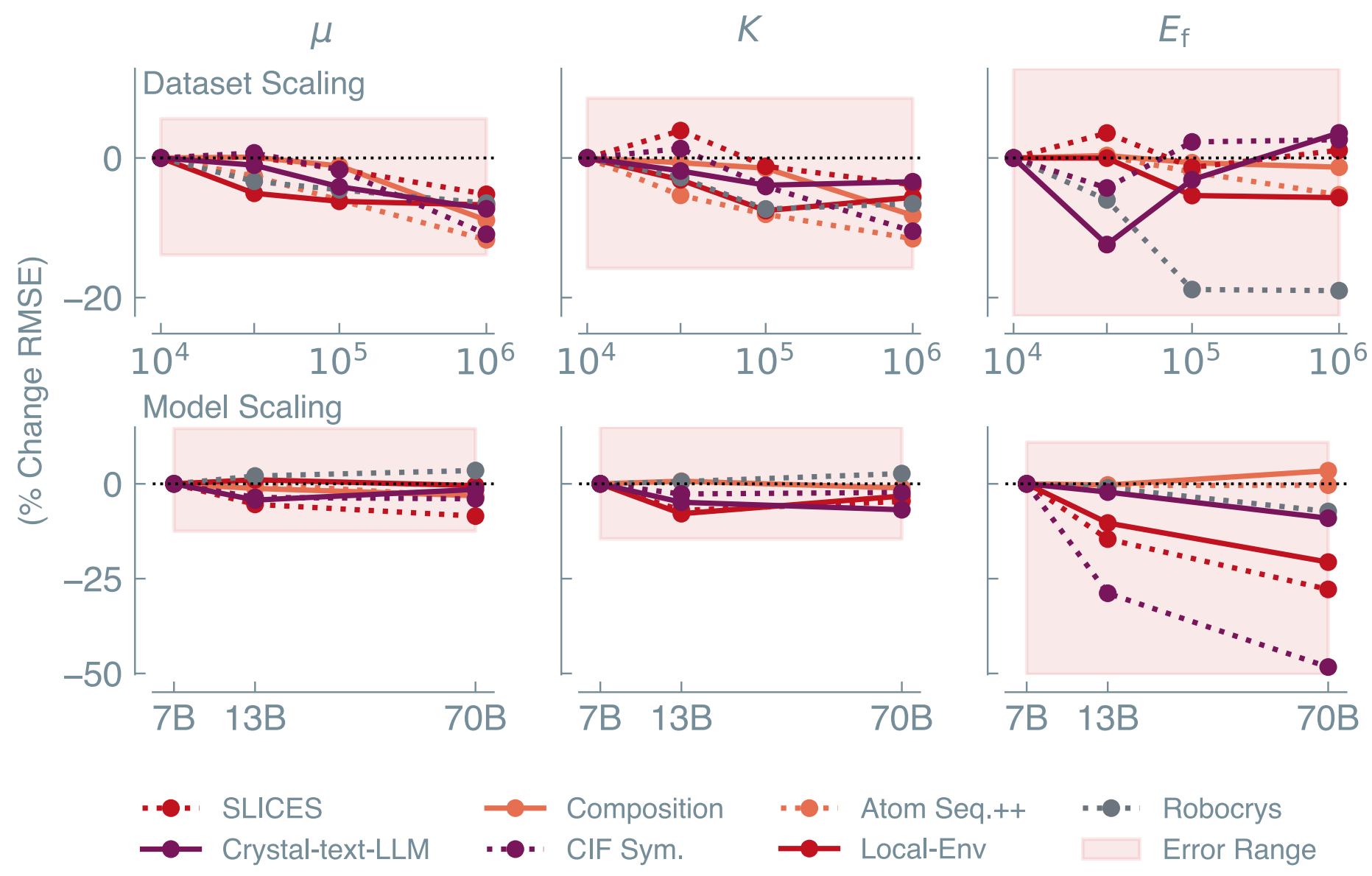

Figure R5: Impact of dataset and model scaling on predictive performance of material representations. This figure illustrates the percentage change in RMSE relative to a baseline (on the left side), for various material representations when scaling (top row) the pretraining dataset sizes for BERT[9] models and (bottom row) the language model parameter count (7B, 13B, 70B parameters) for instruction-tuned LLaMA[10] model. Each column corresponds to a different material property: shear modulus (μ), bulk modulus (K), and formation energy (E_f). Each colored line tracks the mean RMSE percentage change for a specific material representation as either dataset size or model size increases. The baseline for dataset scaling is the mean RMSE achieved with 30,000 structures dataset for that representation. For model scaling, the baseline is the mean RMSE of the 7 billion (7B) parameter model for that representation. Negative percentage changes indicate improved predictive performance (lower RMSE) compared to the respective baseline. The light red shaded region in each subplot defines the overall range of observed RMSE percentage changes across five folds. The upper and lower boundaries represent the global maximum and minimum percentage changes in RMSE, respectively, from all individual cross-validation fold results of all representations. This band thereby encapsulates the full spectrum of performance variation encountered for that property under the given scaling regime. Within each color group (compositional, local, geometric), different linestyles (solid, dotted) distinguish individual representations, robocrys is highligthed with grey colour since apart from being local representation it is a representation based on natural language explanation. The dotted black line is a reference to no change.

Reviewer Point P 2.6 — 5. In Figure 7, which model and input representation does MatText correspond to? Please clarify.

Reply: *We thank the reviewer and have clarified this in the caption of Figure 7. We have revised the caption to include*

> *MatText corresponds to the best-performing BERT-based model using the SLICES representation pretrained on 2M structures.*

Reviewer Point P 2.7 — 6. In section 2.3, the authors mentioned that "Remarkably, we find that for all representations and prediction targets we investigated, there is no effect of either scaling dataset size or model parameter count (we provide more

extensive details in section 5.1)". I believe this contradicts with Figure 6. Where there is a clear trend on Mu and K properties which shows that as you increase the datasize the performance improves. And for Ef, a significant performance improvement is also observed with model scaling. Please clarify as I did not find any details in section 5.1 as mentioned in the paper.

Reply: *The original phrasing was too strong. We have revised the text on to discuss dataset and model scaling with quantification. The revised text now reads,*

> *We find that, for all representations and prediction targets we investigated, dataset scaling (Figure R5, top row) yields minimal changes in RMSE (less than 20%, e.g., an absolute shift of <0.04 for a baseline RMSE of 0.2). For model scaling (Figure R5, bottom row), changes are similarly modest for μ and K. For both scaling approaches E_f shows larger reductions around ~20% with Robocrystallographer representation in data scaling and up to ~50% for some geometric representations in model scaling, though these are not consistent across representations. Importantly, even accounting for these improvements, the absolute performance of LLMs remains far below GNN-based approaches (See Figure 7).*

Second, the reference to Section 5.1 was incorrect. We have removed this cross-reference in the revised manuscript, as the scaling methodology is already described in Section 4.4

Reviewer Point P 2.8 — 1. Citations 4 and 23 seem to be duplicates, also there is an updated peer-reviewed version of this work (https://www.nature.com/articles/s41524-025-01536-2), please check and update other citations accordingly.

Reply: *We thank the reviewer for pointing this out. We have removed the duplicate citation and updated the reference to the published peer-reviewed version.*

Reviewer Point P 2.9 — 2. "...in our calculations we maps chemical..." in section 4.1.1, is this a possible typo?

Reply: *Yes, this was a typo. We have corrected "maps" to "map" in the revised manuscript.*

Reviewer Point P 2.10 — 3. "...(e.g., CIF files) similar to that used for the LLMs)..." in section 4.2.2, possible extra parenthesis?

Reply: *Yes, there was an extra closing parenthesis. We have corrected this in the revised manuscript.*

Reviewer Point P 2.11 — I visited the githup repository of MatText, which has a nice but short README with information on the installation and the basic capabilities. More importantly, the documentation on the related webpage provides a great deal of information and example scripts in a clean and efficient format. I have not run the code, but I expect it to be a relatively smooth experience thanks to these resources.

Reply: *We thank the reviewer for taking the time to explore the MatText repository and for the positive feedback on the documentation.*

REFERENCES

(1) Ruff, R.; Reiser, P.; Stühmer, J.; Friederich, P. Connectivity optimized nested line graph networks for crystal structures. *Digital Discovery* **2024**, *3*, 594–601.

(2) De Breuck, P.-P.; Evans, M. L.; Rignanese, G.-M. Robust model benchmarking and bias-imbalance in data-driven materials science: a case study on MODNet. *Journal of Physics: Condensed Matter* **2021**, *33*, 404002.

(3) Goodall, R. E.; Parackal, A. S.; Faber, F. A.; Armiento, R.; Lee, A. A. Rapid discovery of stable materials by coordinate-free coarse graining. *Science advances* **2022**, *8*, eabn4117.

(4) De Breuck, P.-P.; Piracha, H. A.; Rignanese, G.-M.; Marques, M. A. A generative material transformer using Wyckoff representation. *npj Computational Materials* **2026**.

(5) Ganose, A. M.; Jain, A. Robocrystallographer: automated crystal structure text descriptions and analysis. *MRS Commun.* **2019**, *9*, 874–881.

(6) Bucior, B. J.; Rosen, A. S.; Haranczyk, M.; Yao, Z.; Ziebel, M. E.; Farha, O. K.; Hupp, J. T.; Siepmann, J. I.; Aspuru-Guzik, A.; Snurr, R. Q. Identification Schemes for Metal–Organic Frameworks To Enable Rapid Search and Cheminformatics Analysis. *Cryst. Growth Des.* **2019**, *19*, 6682–6697, DOI: `10.1021/acs.cgd.9b01050`.

(7) Scheidgen, M.; Himanen, L.; Ladines, A. N.; Sikter, D.; Nakhaee, M.; Fekete, Á.; Chang, T.; Golparvar, A.; Márquez, J. A.; Brockhauser, S., et al. NOMAD: A distributed web-based platform for managing materials science research data. *Journal of Open Source Software* **2023**, *8*, 5388.

(8) Lee, K. L. K.; Gonzales, C.; Nassar, M.; Spellings, M.; Galkin, M.; Miret, S. Matsciml: A broad, multi-task benchmark for solid-state materials modeling. *arXiv preprint arXiv:2309.05934* **2023**.

(9) Devlin, J.; Chang, M.-W.; Lee, K.; Toutanova, K. Bert: Pre-training of deep bidirectional transformers for language understanding. *arXiv preprint arXiv:1810.04805* **2018**.

(10) Touvron, H.; Martin, L.; Stone, K.; Albert, P.; Almahairi, A.; Babaei, Y.; Bashlykov, N.; Batra, S.; Bhargava, P.; Bhosale, S., et al. Llama 2: Open foundation and fine-tuned chat models. *arXiv preprint arXiv:2307.09288* **2023**.

RESPONSE TO THE REVIEWERS

REVIEWERS 2 AND 3

Reviewer Point P 1.1 — Figure R1 (or Fig. 8) caption: "(Right) A monotonically increasing curve yields a negative CC-Cliff, indicating that the models struggle more with coordinate information than categorical information." → "coordinate" and "categorical" should be inverted.

Reply: *We thank the reviewer for catching this. A negative CC-Cliff means* $L_{cat,\alpha} \gg L_{coord,\alpha}$*, so the model struggles more with* categorical *information than coordinate information. The caption has been corrected accordingly.*

Reviewer Point P 1.2 — "indicaing" → "indicating"

Reply: *Corrected.*

Reviewer Point P 1.3 — "would indicate a models inability" → "a model's inability"

Reply: *Corrected.*

Reviewer Point P 1.4 — Fig. 2: "($L_{cat,\alpha}$). Which" → ", which"

Reply: *Corrected.*

Reviewer Point P 1.5 — "MODNet, which relies on composition-derived descriptors with limited geometric sensitivity" → This is not strictly composition-derived; structural features are likely involved as well.

Reply: *We have updated the description to "MODNet, which relies on composition and structure-derived features with limited explicit geometric sensitivity"*

Reviewer Point P 1.6 — "Dataset 3, which is more structurally constrained dataset compared to 1 and 2" → "which is *a* more structurally constrained dataset..."

Reply: *Corrected.*

Reviewer Point P 1.7 — "synthetic datasets described in are" → Missing context: "in what?"

Reply: *Corrected to "synthetic datasets used for the coordinate-category experiments described in the main manuscript are constructed from...".*

Reviewer Point P 1.8 — The Matra-Genoa citation requires volume and page numbers.

Reply: *Updated.*

Reviewer Point P 1.9 — A period is missing at the end of the caption of Fig. 1.

Reply: *fixed*

Reviewer Point P 1.10 — "e.e." → "e.g." (p. 2)

Reply: *Corrected; the phrase now reads "for example,".*

Reviewer Point P 1.11 — Commas are missing after "i.e." or "e.g." in multiple instances (e.g., p. 3).

Reply: *Corrected throughout the manuscript.*

Reviewer Point P 1.12 — The label (A) is missing in the caption of Fig. 2.

Reply: *The label **A** is present in the Figure Legends caption of the revised manuscript.*

Reviewer Point P 1.13 — In Fig. 2, there is an extra space after a parenthesis in subfigure A.

Reply: *Corrected; trailing spaces removed from subfigure captions.*

Reviewer Point P 1.14 — "that dependent" → "that depends" or "that are dependent."

Reply: *Corrected to "properties that depend on positional information".*

Reviewer Point P 1.15 — "Our findings... further reveals" → "reveal"

Reply: *Corrected.*

Reviewer Point P 1.16 — Fig. 4 caption: "the framework also supportS" → "supports"

Reply: *Corrected.*

Reviewer Point P 1.17 — "Appendix appendix" → "Appendix" (redundant)

Reply: *Corrected.*

Reviewer Point P 1.18 — Fig. 7 caption: The period is incorrectly spaced far from the caption text.

Reply: *Fixed*
